# The behavioural and neuropathologic sexual dimorphism and absence of MIP-3α in tau P301S mouse model of Alzheimer’s disease

**DOI:** 10.1186/s12974-020-01749-w

**Published:** 2020-02-24

**Authors:** Yao Sun, Yongqing Guo, Xuejian Feng, Meng Jia, Ning Ai, Yue Dong, Yayuan Zheng, Lu Fu, Bin Yu, Haihong Zhang, Jiaxin Wu, Xianghui Yu, Hui Wu, Wei Kong

**Affiliations:** 1 National Engineering Laboratory for AIDS Vaccine, School of Life Sciences, Jilin University, Changchun, 130012 China; 2 State Key Laboratory of Molecular Developmental Biology, Institute of Genetics and Developmental Biology, Chinese Academy of Sciences, Beijing, 100101 China; 3 Department of Pathology, The Second Hospital of Jilin University, Changchun, 130041 Jilin China; 4 Laboratory of Pathogenic Microbiology and Immunology, College of life science, Jilin Agricultural University, Changchun, 130012 China; 5 Key Laboratory for Molecular Enzymology and Engineering, the Ministry of Education, School of Life Sciences, Jilin University, Changchun, 130012 China

**Keywords:** Alzheimer’s disease, Tauopathies, Sexual dimorphism, Tau P301S transgenic mice, Behavior, Tau hyper-phosphorylation, Plasma biomarker

## Abstract

**Background:**

Tau hyper-phosphorylation has been considered a major contributor to neurodegeneration in Alzheimer’s disease (AD) and related tauopathies, and has gained prominence in therapeutic development for AD. To elucidate the pathogenic mechanisms underlying AD and evaluate therapeutic approaches targeting tau, numerous transgenic mouse models that recapitulate critical AD-like pathology have been developed. Tau P301S transgenic mice is one of the most widely used mouse models in AD research. Extensive studies have demonstrated that sex significantly influences AD pathology, behavioral status, and therapeutic outcomes, suggesting that studies using mouse models of AD must consider sex- and age-related differences in neuropathology, behavior, and plasma content.

**Method:**

We systematically investigated differences in tau P301S transgenic mice (PS19 line) and wildtype littermates of different sex behavioral performance, tau neuropathology, and biomarkers in plasma and brain.

**Results:**

Male P301S transgenic mice exhibited significant changes in weight loss, survival rate, clasping, kyphosis, composite phenotype assessment, nest building performance, tau phosphorylation at Ser202/Thr205, and astrocyte activation compared to that of wild-type littermates. In contrast, female P301S transgenic mice were only sensitive in the Morris water maze and open field test. In addition, we characterized the absence of macrophage-inflammatory protein (MIP-3α) and the upregulation of interferon (IFN)-γ, interleukin (IL)-5, and IL-6 in the plasma of P301S transgenic mice, which can be served as potential plasma biomarkers in P301S Tg mice. Male P301S transgenic mice expressed more monokine induced by IFN-γ (MIG), tumor necrosis factor-α (TNF-α), IL-10, and IL-13 than those of female P301S mice.

**Conclusion:**

Our findings highlight sexual dimorphism in the behavior, neuropathology, and plasma proteins in tau P301S transgenic AD mice, indicating that the use of male P301S transgenic mice may be more suitable for assessing anti-phosphorylated tau therapeutic strategies for AD and related tauopathies, and the MIP-3α may be a new potential plasma biomarker.

## Background

Alzheimer’s disease (AD) is a progressive neurodegenerative disorder and becomes the most common form of dementia among the elderly in contemporary society [1]. The neuropathological features of AD include extracellular senile plaques formed by amyloid-β (Aβ), intraneuronal neurofibrillary tangles (NFTs) accumulated by hyperphosphorylated Tau protein, and widespread neuronal loss [1]. Hyperphosphorylated tau protein is the main component of NFTs in neurons, which eventually leads to the neuronal death [2]. Tau protein is a major microtubule-associated protein (encoded by *MAPT* gene) in the mammalian nervous system that regulates the assembly and stability of microtubules and axonal transport under physiological conditions [3]. However, under pathophysiological conditions, abnormal hyperphosphorylation of tau at numerous toxic epitopes has been extensively reported in the context of AD and related tauopathies, including corticobasal degeneration (CBD), progressive supranuclear palsy (PSP), Pick’s disease (PD), and frontotemporal lobar degeneration (FTLD) [4, 5]. In familial tauopathy patients, genetic mutations including G272V, P301L, P301S, V337M, and R406W have been identified, which could promote the aggregation of tau to form paired helical filaments (PHFs) and neurofibrillary tangles (NFTs) [6, 7]. After that, a variety of tau transgenic (Tg) mice have been generated and become essential tools for exploring the mechanism of tau dysfunction and developing the therapeutics for neurodegenerative diseases. Tg mice expressing human MAPT (1N4R isoform) bearing the P301S missense mutation, termed PS19 (P301S Tg) mice, have become an indispensable tool in research on AD and related tauopathies [8]. Tau filaments develop in P301S Tg mice at 6 months of age and are progressively enriched in parallel with prominent neuronal death and brain atrophy by 9–12 months of age.

Sex affects the etiology, pathological symptoms, and therapeutic outcomes of several neurologic diseases. Initial studies suggested that AD prevalence is higher in women than in men, and the median age of death among women with advanced dementia is higher than that among men, but females live longer than males because of fewer comorbidities [9–11]. Sex also influences the effect of AD risk factors. As the most important genetic risk factor for late-onset AD, APOE4 has been demonstrated to be a greater AD risk factor for women than for men. Furthermore, sex steroid hormones in the plasma of AD patients are also sexually divergent, leading to different detection and treatment of AD patients [12]. These findings indicated that men and women exhibit striking differences in the development and progression of AD, highlighting the importance of understanding the influence of sex differences on the ethology, pathobiology, and serology of AD model mice. In P301S Tg mice, which is widely used in the preclinical AD drug development, the levels of carbonyls in mitochondria, citrate synthase, manganese superoxide dismutase (MnSOD), cytochrome C, and cytochrome C oxidase exhibited incongruous convert were sexually dimorphic [13]. However, there is no thorough and precise characterization about the alternation of development, progression, and plasma protein of AD in P301S Tg mice considering both sex and age factor.

In the current study, we systematically investigated the discrepancies in behavior, tau pathology, and proteins expressed in plasma and brain between tau P301S transgenic mice of different sexes and ages and examined the correlations between these factors during tauopathy progression. Additionally, we investigated differentially expressed protein in plasma and brain in P301S Tg mice and littermates. Our findings demonstrated remarkably sexual dimorphism in the behavior, neuropathology, and biomarkers in tau P301S transgenic mice and indicated that the use of male P301S transgenic mice may be more suitable for assessing anti-phosphorylated tau therapeutic strategies for AD and related tauopathies.

## Materials and methods

### Mice

Heterozygous transgenic mice (Tg(Prnp-MAPT*P301s)Ps19Vle/JNju) were purchased from the Model Animal Research Center of Nanjing University (Nanjing, China). The transgenes were driven by the murine prion protein (Prnp) promoter on a B6C3 background and expressed the P301S human 1N4R Tau isoform on the Chromosome 3 of mice [14]. Genetic background and age-matched non-transgenic littermates were used as wild-type (WT) control mice. Four cohorts were employed in this monitoring experiment which included P301S male mice, P301S female mice, and sex-matched WT mice in each group, and 5–10 mice of each genotype were sacrificed at the age of 3, 6, 9, and 12 months old, respectively, for histological analysis and Western blot of brain homogenate (Additional file 2: Table S1). Blood samples from all animals were collected into EDTA tubes from the submandibular vein plexus every month, followed by centrifugation at 1150 *g* for 30 min. The plasma was separated to detect the concentration of total human Tau, chemokines, and inflammatory cytokines. One group of mice was used to monitor the behavioral changes every month throughout 3 to 12 months of age, including body weight, grip force test, accelerating rotarod test, stride length, ledge test, hindlimb clasping, gait grade, and kyphosis grade. Nest building tests were implemented at the age of 3, 6, 9, and 12 months old. Four groups of animals were tested in the Morris water maze (MWM) at the age of 3, 6, 9, and 12 months old, respectively. After the MWM test, the mice were deeply anesthetized with pentobarbital (100 mg/kg) and perfused with phosphate-buffered saline (PBS). Organs were harvested for pathological examination. The heart, liver, spleen, lung, and kidney were subjected to hematoxylin and eosin staining (H&E). Half of the brain was homogenized for Western blot detection, and the other hemisphere was submerged in 4% paraformaldehyde in PBS for immunohistochemical (IHC) detection.

All animals were housed in individually ventilated cages (IVC) in Animal Experimental Platform, Core Facilities for Life Science, Jilin University. Animals were housed two or three per cage and maintained at 20–22 °C on a 12-h dark/light cycle in 40–60% humidity.

### Morris water maze test

MWM test was performed on P301S transgenic mice at age of 3, 6, 9, and 12 months old with age- and sex-matched WT littermate mice to evaluate the capability of spatial learning, which strongly correlated with hippocampal synaptic plasticity [15]. The diameter of the stainless-steel water pool was 120 cm, a circular platform with a diameter of 12 cm was placed at the center of the fourth quadrant (southeast quadrant, SE quadrant) and submerged 1 cm below the water surface. Before the training test, water was uniformly dispersed with edible titanium dioxide pigments. Temperature was adjusted to 20 ± 1 °C. Each mouse was carefully released into the water facing the tank wall, and allowed to search for the hidden platform for 60 s. Mice were then guided to climb up the platform and stayed there for 30 s to remember the surrounding objects. Then the mice were placed in an adjacent quadrant, and the training trials were repeated for the other two quadrants. The training test lasted for five consecutive days. A water maze video analysis system (ZS Dichaung, Beijing, China) was used to record the latency to search for the platform. The probe trial was implemented on the sixth day. The hidden platform was removed and each mouse was released into the diagonal quadrant (northwest quadrant, NW quadrant) of the target platform area. The movement trajectories and number of times each mouse traversed the original platform area were recorded over a 60-s exploration time.

### Accelerating rotarod

ZS-YSL-4C rotary fatigue apparatus (ZS Dichaung, Beijing, China) was used to gauge the balance, equilibrium, and motor coordination of mice. Before the first test at the age of 3-month-old, all mice were trained to maintain their balance on a rotating shaft for 5 min with increasing maximum speed (from 10 to 40 rpm over three consecutive days, three times per day). The accelerating rotarod test proceeded every month. Mice were placed on the rotating shaft and the machine was initiated with an increased speed from 5 to 40 rpm. Time taken to fall down during the 10-min session was recorded as fall latency.

### Grip strength test

ZS-YLS-13A grip force apparatus (ZS Dichaung, Beijing, China) was used to evaluate the muscle injury and nerve damage of mice. The mice lay on a grasping board and gripped sticks. An experimenter pulled the tail back horizontally along the grasping board, and maximum grip force was recorded. All mice were tested three times every month, and the average was calculated as the grip force at that time point.

### Stride length

Mice were inked on their right rear foot and released on one end of a 40-cm track under light, which had a width of 4.5 cm and a dark box at the opposite end. The length between two adjacent footprints was measured, and the average length was calculated.

### Composite phenotype scoring system

Cerebellar ataxia appraisal of mouse models can be measured using a composite phenotype scoring system, including hind limb clasping, ledge test, gait, and kyphosis [16]. Each measured item is scored on a scale of 0 to 3 which represents the best to worst phenotype, with a combined total of 0 to 12 for all four measures. Three to four trained experimenters blinded to groups graded all mice independently every month. The average score of each mouse at each time point was calculated.

### Nest building test

The nest building test is one of the method used to evaluate murine cognition. The nest building test started at evenfall. All mice was allocated to a single cage containing new aseptic sawdust and two pieces of 5-cm square cotton in the center of the cage, then turned off the light. Three trained experimenters blinded to groups graded all the mice independently after 16 h and 24 h. The scoring rule has been described in our previous research [17]. Briefly, score 1 indicates there was no noticeable touching, score 2 indicates the cotton was partially torn up, score 3 indicates that the nest was partially complete but lower than the mouse’s head, and score 4 indicates that the nest was nearly perfect or perfect. All mice were tested at the age of 3, 6, 9, and 12 months old.

### Open field test

The open field test was used to measure anxiety and locomotor behavior. Mice naturally prefer to wander around a protective wall rather than central of open field, but a competing instinct for foraging will drive mice to explore open spaces. In the first 5 min after placing mice in the open field box, most demonstrate the instinct of eluding danger, after which they adapt to the new circumstance and exhibit exploratory responses [18]. To measure the behavioral differences among four groups of mice, we tracked movement trajectories for 10 min in a square white box (50 cm × 50 cm × 50 cm). A video analysis system (Super Maze, ZS Dichaung, Beijing, China) was used to record the trajectory of mice and motions such as rearing (vertical activity), grooming, and defecation. Movement parameters such as distance moved, time spent moving, and location were recorded, as well as the frequency and duration of behavior (such as rearing, and grooming), and the autonomic nervous system measures were also analyzed. The open field box was divided into 25 quadrants, comprising nine central squares, 14 fringe squares, and four corner squares.

### Western blot

Right brain hemisphere of each mouse were deeply frozen in liquid nitrogen immediately after collection and transferred to − 80 °C before homogenization for Western blot detection. Brain samples were weighed and homogenized in 15 μL RAB buffer (100 mM MES, 1 mM EDTA, 2 mM dithiothreitol, 0.75 M NaCl, 0.5 mM MgSO_4_, pH 6.8) with protease and phosphatase inhibitor MIX (1 mM PMSF, 50 mM sodium fluoride, 1 mM sodium pyrophosphate, 1 mM sodium orthovanadate) per milligram in Bead Mill Homogenizer (Bead Ruptor 24 Elite, OMNI International, Inc., 935-C Cobb Place Boulevard Kennesaw, GA 30144) at a speed of 5.65 m/s for 45 s with a 30-s pause between two cycles. Then 1 mL brain homogenate suspension was transferred to a 1.5 mL microfuge tube (357448, Beckman Coulter, Inc., 250 S Kreamer Bivd Brea, CA 92821) and centrifuged at 50,000 *g* for 20 min at 4 °C (Optima Max-XP, Beckman Coulter, Inc., CA). The supernatant was saved as RAB-soluble fraction (aqueous fraction). The pellet was resuspended with 1 mL RIPA buffer (150 mM NaCl, 50 mM Tris, 25 mM EDTA, 1% Triton X-100, 0.5% deoxycholic and 0.5% (w/v) SDS, pH 8.0) with protease and phosphatase inhibitor MIX and centrifuged at 50,000 *g* for 20 min at 4 °C to collect the supernatant as RIPA-soluble fraction (detergent soluble fraction). The pellets were further disposed by 0.5 mL urea buffer (8 M urea and 5% (w/v) SDS, pH 7.2) at 2–8 °C overnight and centrifuged to separate the supernatant as urea-soluble fraction (insoluble fraction). The three fraction samples were subpackaged in an EP tube followed by cryopreservation at − 80 °C before detection. Freeze/thaw cycles were avoided. Aliquot samples of each group were mixed together before analysis. Homogenate samples were separated by 12% SDS-PAGE and transferred onto 0.45 μm nitrocellulose membranes (Whatman, Kent, UK) for RAB and RIPA fraction, or 0.45 μm PVDF membranes (GE healthcare) for urea fraction. Blotting membranes with 10% skimmed milk powder (dissolved in PBS) at room temperature for 20 min, then incubated with primary antibodies at 2–8 °C overnight followed by horseradish peroxidase (HRP)-conjugated secondary antibody at room temperature for 1 h. Blots were incubated with ECL solutions (MA0186, Dalian Meilun Biotechnology Co., LTD, China) and exposed under a chemiluminescent imaging system (Tanon 6100, Tanon Science & Technology Co., Ltd., Shanghai, China).

### Enzyme-linked immunosorbent assay

For monitoring total human Tau in the plasma of mice at different ages, an enzyme-linked immunosorbent assay (ELISA) kit (KHB0041, Invitrogen, CA) was employed for quantification of plasma which comprised aliquoted mixtures of individuals in each group before detection. Triplicate samples were performed. Concentration was calculated according to the standard curve.

### Immunohistochemistry

Left brain hemisphere of each mouse was fixed with 4% paraformaldehyde for 2 days, followed by graded dehydration with alcohol from a concentration of 70% to 100%. The brain was hyalinized with dimethylbenzene twice for a total time of 5 h, followed by wax immersion and paraffin embedding. Ten consecutive 5-μm-thick sagittal sections cut at approximately 600–700 μm from the sagittal suture were used to examine the expression of human tau, phosphorylated tau, astrocytes, microglia, and Aβ plaques. Briefly, paraffin sections were subjected to antigen retrieval in citrate buffer (pH 6.0) using a pressure cooker for 3 min and blocked with 3% hydrogen peroxide and 10% goat serum sequentially. Primary antibody was added onto brain sections and incubated at 2–8 °C overnight. Slides were washed with TBST (pH 7.2–7.4), then Primary Antibody Amplifier (UltraVision Quanto Detection System HRP DAB, thermo, CA) was applied, and sections were incubated for 30 min at room temperature, followed by HRP Polymer for another 30 min. Coloration with DAB chromogen for 1 min and hematoxylin staining was performed after washing in water, then sections were sealed with neutral gum. Brain sections were imaged using Aperio Digital Pathology (Aperio CS2, Leica biosystem imaging, Inc., German) and analyzed by Aperio ImageScope (Leica Biosystems, Buffalo, Grove, Ill). The number of positive pixels in the hippocampus or cortex per square micrometers was quantifies and recorded as IOD/area.

### Hematoxylin and eosin staining

Heart, liver, spleen, lung, and kidney tissue of all mice were submerged in paraformaldehyde after removal, followed by dehydration, wax immersion, and paraffin embedding as described in Materials and methods, “Immunohistochemistry” section. Sections were cut at a thickness of 5 μm, followed by dewaxing, staining with hematoxylin for 5 min, and eosin for 3 min sequentially, then sealed the sections and histopathological analysis.

### Flow cytometry

Plasma and RAB fraction of brain homogenates were applied for cytokine and chemokine detection with BioLegend LEGENDplex™ multi-analyte flow assay kit (740,029 & 740,007, BioLegend, USA). Duplicate samples were used to calculate the concentration of factors at each check point.

### Antibodies

Human total Tau mAb (HT7, MN1000, Thermo Fisher Scientific, IL, USA) was used at a ratio of 1:2000 for Western blot. GAPDH mAb (60004-1-lg, Proteintech, Chicago, IL, USA) was used at a ratio of 1:10,000 for Western blot. Anti-FOX3 (NeuN) mAb (1B7, 834501, Biolegend, USA) was used at a ratio of 1:2000 in Western blot. Phospho-Tau (Ser404) Polyclonal Antibody (44-758G, Thermo Fisher Scientific) was used at a ratio of 1:300 and 1:1000 for IHC and Western blot, respectively. Phospho-Tau (Ser202/Thr205) mAb (AT8, MN1020, Thermo Fisher Scientific) was used at a ratio of 1:50 and 1:2000 for IHC and Western blot, respectively. Phospho-Tau (Ser396) mAb (PHF-13, 829,001, BioLegend, USA) was used at a ratio of 1:1000 and 1:3000 for IHC and Western blot, respectively. Anti-GFAP mAb (2E1.E9, 644,702, Biolegend, USA) was used at a ratio of 1:1000 and 1:3000 for IHC and Western blot, respectively. Anti-Iba1 rabbit monoclonal antibody (EPR16588, ab178846, Abcam, USA) was used at a ratio of 1:1000 and 1:3000 for IHC and Western blot, respectively. Peroxidase-conjugated AffiniPure goat anti-rabbit IgG (H + L) antibody (111-035-144, Jackson ImmunoResearch, USA) was used at a ratio of 1:100,000 for Western blot. Peroxidase-conjugated AffiniPure goat anti-mouse IgG (H + L) antibody (115-035-003, Jackson ImmunoResearch, USA) was used at a ratio of 1:100,000 for Western blot.

### Statistical analysis

Statistical analysis was performed using SPSS software (version 20.0, IL, Chicago, USA) and GraphPad Prism 8.0 (GraphPad Software, Inc.) with a probability level set at 95%. Results are described as mean ± S.E.M. The relations among the measured data of behavioral or pathological monitoring at consecutive time points were analyzed using Mauchly’s test of sphericity. Multivariate ANOVA was employed when *p* ≤ 0.05. Bonferroni-corrected *t* test was otherwise applied.

## Results

### P301S Tg mice exhibited sex-specific differences in behavior

To determine the applicability of behavioral tests for male and female P301S Tg mice, we analyzed sex- and age-related changes in behavior, including body weight, grip force test, accelerating rotarod test, stride length, ledge test, hind limb clasping, gait grade, kyphosis grade, nesting, and Morris water maze.

### Weight change and survival

**Fig. 1:**
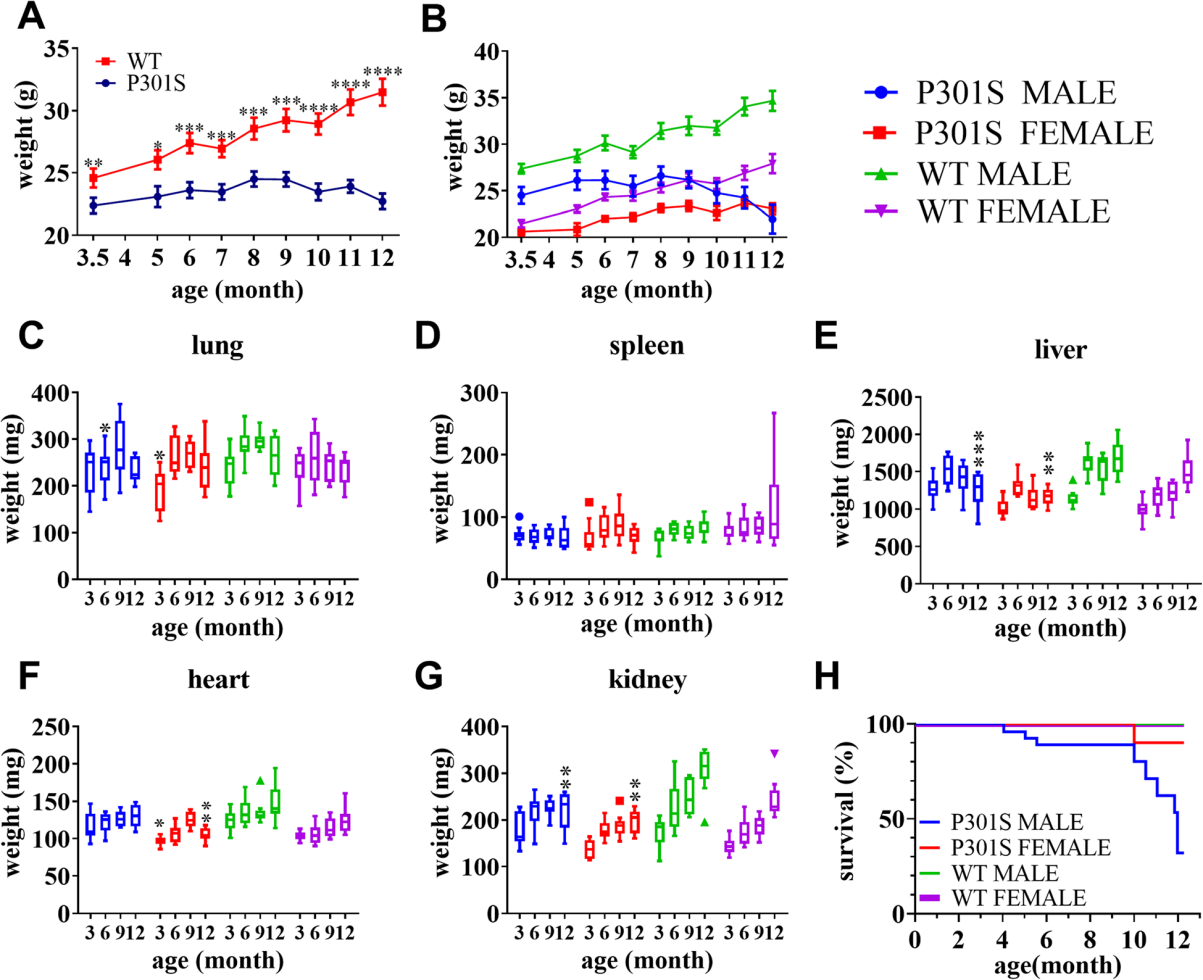
Weight change, organ weight changes, and mouse survival. **a** Shows the weights of P301S transgenic mice (P301S) and wild-type mice (WT) when combining homotypic male and female mice into a single group, respectively. Significant effect was observed between P301S and WTs. Data are presented as mean ± S.E.M. *p < 0.05, **p < 0.01, ***p < 0.001, ****p < 0.0001. **b** Shows the weight changes of the four groups of mice. **c** to **g** shows the weight changes of lung, spleen, liver, heart, and kidney in each subgroup in the observation period, respectively. Significant effects were observed between sex-and age- matched P301S and WTs. Data are presented as mean ± S.E.M. *p < 0.05, **p < 0.01. **h** Shows the survival of the four groups during 1-year period. The line in (**b** to **h**): blue: P301S MALE, red: P301S FEMALE, green: WT MALE, purple: WT female

We first assessed body weights in P301S Tg mice and WT littermates at different ages and sexes. Two cohorts of Tg mice with age and sex-matched WT littermates were used for long-term behavioral testing at 3.5-, 5-, 6-, 7-, 8-, 9-, 10, 11-, and 12-month-old time points. There was a significant difference in weight between P301S mice and WT mice across all ages (Fig. 1a; Additional file 1: Figure S1A; Additional file 2: Table S2). P301S Tg mice reached maximum weight at 8 months old, while WT mice continued to gain weight with age. Both male and female WT mice exhibited a similar weight growth curve from the age of 3.5 months to 12 months. However, the weights of male WT mice were significantly higher than those of female WT mice at all disease stages (Fig. 1b; Additional file 2: Table S3). We also observed that P301S Tg mice exhibited sex-specific differences in weight changes. Male Tg mice gained weight slowly, similar to female Tg mice before 8 months of age, but this declined sharply thereafter (Fig. 1b; Additional file 1: Figure S1B). Conversely, the weights of female P301S Tg mice showed small changes from the age of 8 months to 12 months. Taken together, P301S Tg mice exhibited sex-specific differences in weight changes, male Tg mice showed a more noticeable decrease in weight than that of female mice at the late stage of disease progression.

Considering the significant differences in weight changes between P301S mice and WT littermates, we also assessed weight changes of different organs including the heart, liver, lung, kidney, and spleen. The weights of the lung and spleen in P301S mice showed a similar change tendency to that of WT mice (Fig. 1c, d), without abnormal pathology in HE slides (Additional file 1: Figure S2, Figure S3). Consistent with the changes in body weight, liver weights in tau Tg mice reached maximum at the age of 6 months, and subsequently dropped dramatically until 12 months of age, revealing impaired liver function in P301S Tg mice due to the development of tau pathology (Fig. 1e). However, we did not observe any significant pathological changes in both Tg and WT mice by HE staining, except slight nucleolar enlargement in the livers of 6- to 12-month-old Tg mice (Additional file 1: Figure S4). Weights of the heart and kidney in both male and female P301S mice changed minimally with age, while those in WT mice increased in an age-dependent manner (Fig. 1f, g). HE staining of heart tissue revealed myoneme atrophy and widened gaps between myofilaments in Tg mice across all periods, but normal histology was observed in WT littermates (Additional file 1: Figure S5). No obvious pathological changes were observed in the kidney (Additional file 1: Figure S6). These changes in the organs of P301S Tg mice suggested metabolic abnormalities in Tg mice.

We also analyzed the survival rate of different mouse cohorts. As shown in the survival analysis in Fig. 1h, all WT mice remained alive for more than 1 year. For P301S Tg mice, female Tg mice begin to die at 10 months old, and 90% of animals were still alive after 1-year observation. In contrast, male P301S Tg mice started to die at 4 months of age and final survival rate dropped to 32% at 12 months of age. Thus, female P301S Tg mice have a significant survival advantage over male Tg mice, probably due to fewer comorbidities.

### P301S mice exhibited sex differences in coordination score system

P301S mice present with muscular atrophy and motor dysfunction as the disease develops [19]. We therefore applied tests for grip strength, accelerating rotarod, stride length, and coordination score system to determine the impact of sex differences on motor dysfunction in P301S Tg mice.

**Fig. 2:**
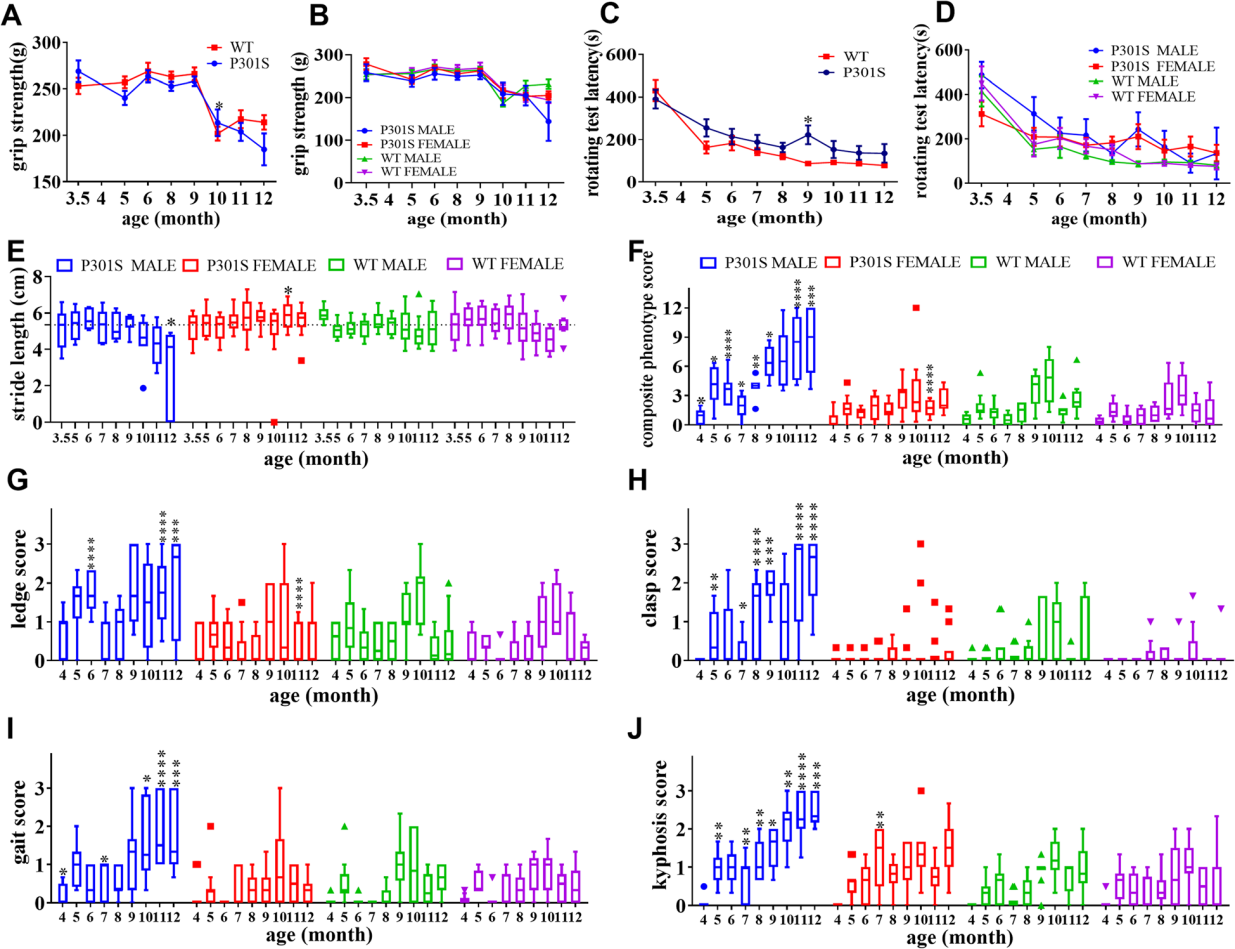
Behavioral tests for limb strength and coordination. Grip strength change. **a** Shows grip strength changes of homotypic mice. **b** Shows grip strength changes of the four groups of mice. Data are presented as mean ± S.E.M. Latency of accelerating rotarod tests. **c** shows rotarod test latency changes of homotypic mice. **d** shows the rotarod test latency changes of the four groups of mice. Data are presented as mean ± S.E.M. Stride length tests change of mice. Data shown in (**e**) is presented as a boxplot with a median line in the box, a 75th percentile line on the top of box (Q3), a 25th percentile line at the bottom of the box (Q1), a maximum value bar calculated by Q3 + 1.5IQR (interquartile distance), a minimum value bar calculated by Q1–1.5IQR, and some abnormal values distant to the box. Dashed line denotes the median of male P301S mice stride length at the age of 3 months old. Composite phenotype scoring system test. **f**–**j** Shows the changes of composite phenotype total score, ledge test, clasp test, gait test, and kyphosis test, respectively, in all four groups. Statistical analysis was performed between sex-and age-matched P301S and WTs. *p < 0.05, **p < 0.01, ***p < 0.001, ****p < 0.0001

In the grip strength test, the strength of P301S Tg mice was significantly reduced at the late disease stage compared to that of WT mice (Fig. 2a; Additional file 2: Table S2). Male but not female Tg mice exhibited decreased grip strength from the age of 10 to 12 months when compared with sex-matched WT littermates (Fig. 2b; Additional file 2: Table S3). Hence, the difference in grip strength between WT and P301S Tg mice was mainly due to sex.

We employed the accelerating rotarod test to evaluate motor performance and balance. Compared with the WT group, P301S Tg mice demonstrated 10% prolonged latency (Fig. 2c; Additional file 2: Table S2). There was no significant latency difference between male and female Tg mice (Fig. 2d; Additional file 2: Table S3).

We performed the stride length test to compare muscular function of hind limbs between P301S Tg and WT mice. There were no significant differences between these two groups (Additional file 2: Table S2). However, we observed that male Tg mice showed a gradually shortening stride length with age. In contrast, female P301S Tg mice maintained a steady curve of stride length with aging (Fig. 2e; Additional file 2: Table S3). Thus, male P301S Tg mice showed more serious damage in motor ability, and male Tg mice were more sensitive than female mice in the stride length test.

The coordination score system consisted of four experiments including ledge test, gait, hind limb clasping, and kyphosis, which individually or in combination characterized equilibrium and muscular atrophy of mice. When we first assessed P301S Tg and WT mice in the ledge test, both groups exhibited no significant differences in coordination (Additional file 1: Figure S7B; Additional file 2: Table S2). Similarly, P301S Tg and WT mice performed similarly in the gait study (Additional file 1: Figure S7D). However, when comparing the behavior of P301S Tg and WT mice in the hind limb clasping and kyphosis test, Tg mice presented with a state of tauopathy and performed worse than WT mice did (Additional file 1: Figure S7C, Figure S7E). Furthermore, when taking sex into account, we observed that female Tg mice displayed similar results to those of female WT mice in all four measurements (Fig. 2g–j; Additional file 2: Table S3). In contrast, male P301S Tg mice exhibited a progressive tauopathy phenotype that was significantly different from that of WT male littermates beginning at 5 months, indicating a more notable impairment of coordination and muscle function in male P301S Tg mice but not in female mice.

When we combined the ledge walking, gait, clasping, and kyphosis assessments into a composite phenotype score for each individual mouse, we observed that male P301S Tg mice exhibited an increasing composite phenotype score that was significantly different from male WT littermates beginning at 4 months, consistent with the progressive nature of AD (Fig. 2f). Nevertheless, female P301S Tg mice showed a similar composite phenotype score as female WT mice did, suggesting that female Tg mice were not sensitive in this composite phenotype assessment (Additional file 2: Table S3).

### Male and female P301S mice differed in cognitive impairments

**Fig. 3:**
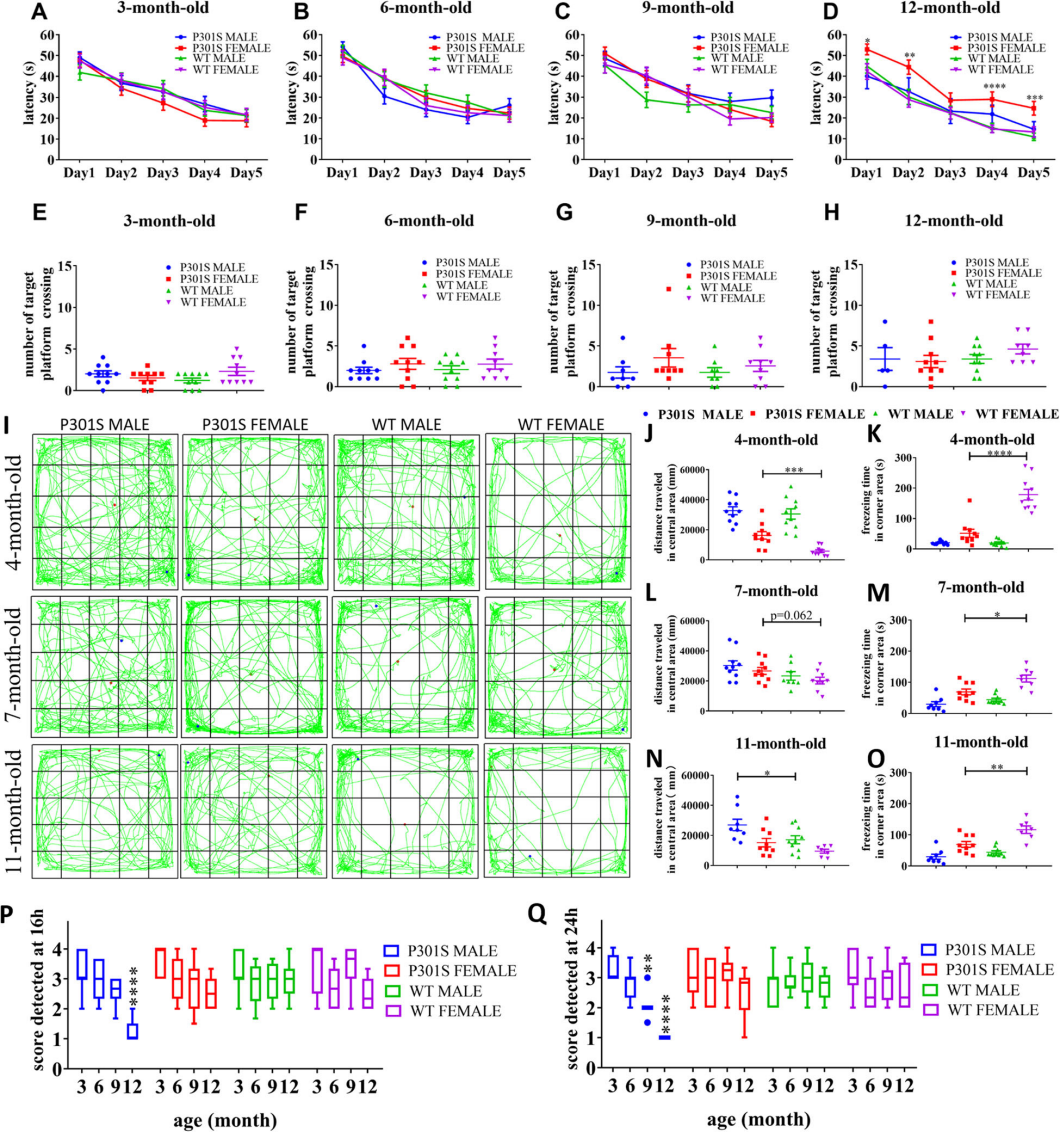
Behavioral tests of memory and anxiety. Morris water maze (MWM)**. a**–**d** Shows the latency of four groups during 5-days training test at 3, 6, 9, and 12 months old, respectively. **e**–**h** Shows the number of target platform crossings of four groups of mice in the probe test at 3, 6, 9, and 12 months old, respectively. Data are presented as mean ± S.E.M. Statistical significance was calculated by ANOVA between the sex-and age- matched P301S and WTs. *p < 0.05, **p < 0.01, ***p < 0.001, ****p < 0.0001. Open field test. All mice were transferred to the laboratory 1 h before testing. **i** Shows the trajectory of mice running during 10 min in the OFT box recorded by a tracking system. Red point in the figure represents the start position of mice, and the blue point indicates the end position. **j**–**n** Shows the distance traveled in the nine squares of the box (central area) at the age of 4, 7, and 11 months, respectively. **k**–**o** Shows the freezing time in the four corner squares of the box (corner area) at the age of 4, 7, and 11 months, respectively. Data are presented as mean ± S.E.M. and scatter. *p < 0.05, **p < 0.01, ***p < 0.001, ****p < 0.0001. Nest building test. Nest building test scored at the 16th and 24th hour just after equal amounts of cotton were placed in each cage and lights were turned off. **p**, **q** Show the scores at the 16th and 24th time point, respectively. Statistical analysis was performed between the sex-and age-matched P301S and WTs. **p < 0.01, ***p < 0.001, ****p < 0.0001

To determine if there was sex-dependent memory and cognitive impairments in P301S Tg mice, we assessed spatial learning and memory capacity of Tg mice and WT control mice at different ages using the MWM. Four cohorts of Tg mice and their littermates were utilized for MWM at the age of 3, 6, 9, and 12 months old. Before 9 months of age, Tg mice did not differ from WTs in latency to find the hidden platform during the acquisition phase (Fig. 3a–c; Additional file 1: Figure S9; Additional file 2: Table S4, Table S5), number of crossings over the target platform, or time spent in the target quadrant in the probe trial (Fig. 3e–g; Additional file 1:Figure S8A-L; Additional file 2: Table S4, Table S5), indicating that cognitive capacity was not impaired in 9-month-old Tau Tg mice. We noticed that 12-month-old female P301S Tg mice showed striking longer escape latencies than those of age-matched WT mice during acquisition and slightly decreased number of crossings over the target platform on the probe trial, although these differences were not significant between Tg mice and WTs (Fig. 3d, h). In contrast, no significant differences in performance in the MWM were observed between male Tg mice and controls, potentially due to rapid dyskinesia in male tau P301S Tg mice. This highlighted later memory decline of female Tg mice which began from 12 months old, and a sex-specific response to memory tests among Tau P301S Tg mice.

We also used the open field test (OFT) to analyze the influence of sex on exploratory behavior and general activity of P301S Tg mice. Our results demonstrated an age-dependent decrease in exploratory activity in both P301S mice and non-Tg mice, and Tg mice typically exhibited longer travel distances and higher movement velocity than that of WTs. This diversity was amplified in females (Fig. 3i; Additional file 1: Figure S10A-B). Compared to female WT mice, female P301S mice displayed enhanced levels of locomotor activity, significantly increased freezing time (Additional file 1: Figure S10E), and more frequent appearances in the central area despite moving (Fig. 3j, l, n) or being in a silent state (Additional file 1: Figure S10F). This was especially evident for freezing time in the corner area (Fig. 3k, m, o). However, we also observed that the differences between female Tg mice and sex-matched WTs decreased over time. In addition, similar hyperactivity state made it difficult to distinguish male P301S mice and WT littermates. Rearing and grooming behavior (Additional file 1: Figure S10G-I) as well as autonomic nervous system (Additional file 1: Figure S10J) activity did not differ among Tg and WT mice. Thus, female P301S Tg mice were more suitable for this measurement at younger ages.

Non-maternal nest-building performance is sensitive to hippocampal damage and is used in murine models of psychiatric disorders [20–23]. We therefore assessed the nest-building capacity of P301S Tg mice of different sexes and ages. Compared to WT littermates, P301S Tg mice showed significantly decreased nest-building capacity throughout disease progression (Additional file 1: Figure S11; Additional file 2: Table S2). Furthermore, male P301S Tg mice exhibited a gradual decrease in nest-building ability from 6 to 12 months old, which was substantially different from the steady building capacity of male WT control mice (Fig. 3p–q; Additional file 2: Table S3). In contrast, female Tg mice performed similarly to WT mice in the nest-building test throughout disease progression. Hence, nest-building performance is a valid behavior measurement for TauP301S Tg mice, and male but not female Tg mice are applicable for this assessment. Further, the 24 h-detection time point is most sensitive.

### Pathological tau development in P301S transgenic mice of different ages and sex

**Fig. 4:**
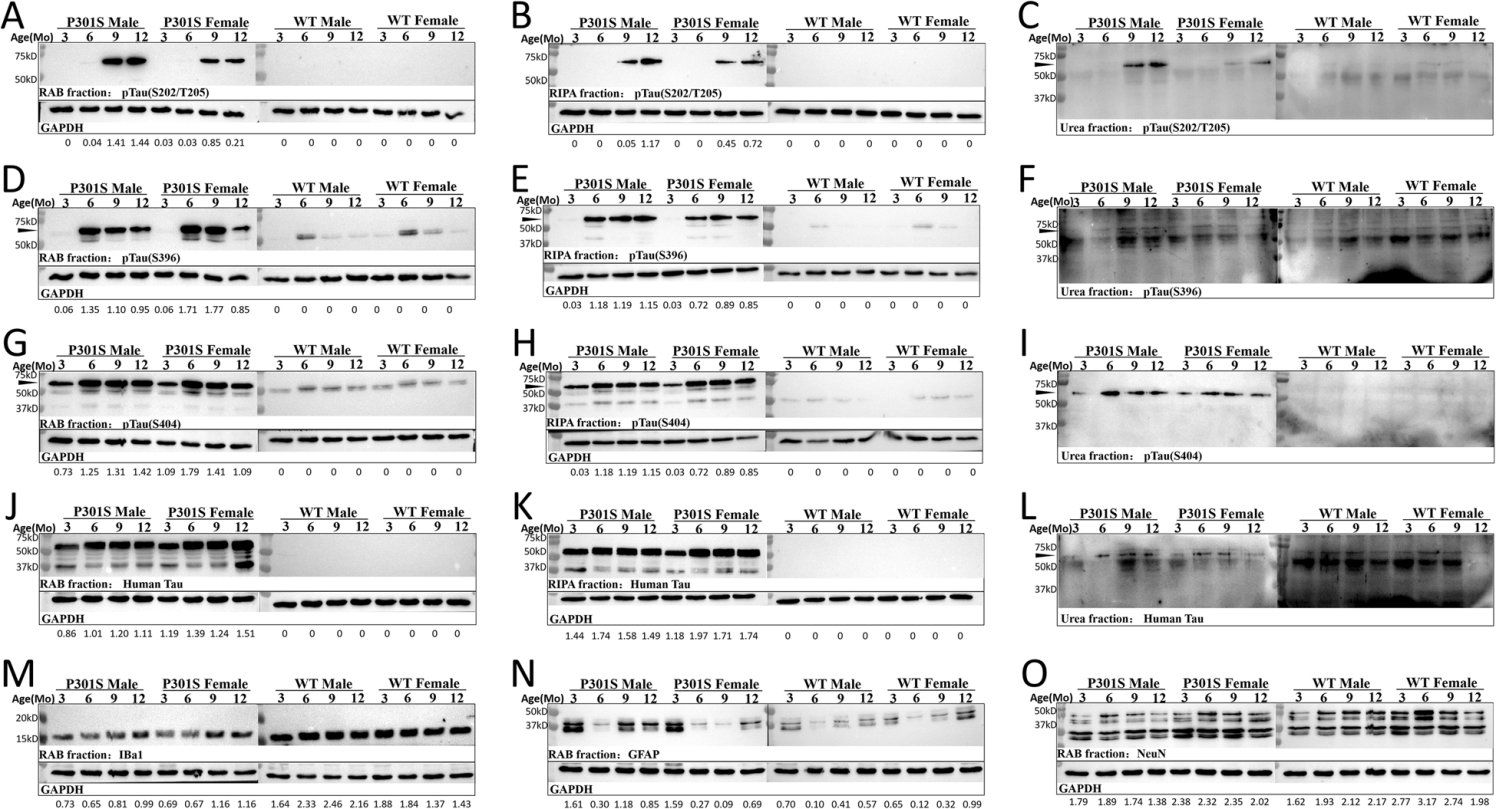
Western blot of mouse brain homogenates. **a**–**c** Show the immunoreactivity of RAB fraction, RIPA fraction, and urea fraction stained by anti-pTau(S202/T205) (AT8) primary antibody, respectively. **d**–**f** Show the immunoreactivity of RAB fraction, RIPA fraction, and urea fraction stained by anti-pTau(S396) (PHF13) primary antibody, respectively. **g**–**i** Shows the immunoreactivity of RAB fraction, RIPA fraction, and urea fraction stained by anti-pTau(S404) polyclonal antibody, respectively. **j**–**l** Showed the immunoreactivity of RAB fraction, RIPA fraction, and urea fraction stained by anti-human Tau(HT7) primary antibody, respectively. **m** Shows the immunoreactivity of RAB fraction stained by Iba1 (EPR16588) (recognize active microglia) primary antibody. **n** Shows the immunoreactivity of RAB fraction stained by anti-GFAP (2E1.E9) (recognize astrocyte) primary antibody. **o** Shows the immunoreactivity of RAB fraction stained by anti-FOX3 (NeuN, 1B7) primary antibody. The relative quantification of the target lane to the lane of GAPDH by ImageJ is marked under each lane. The black triangles indicate the positive bands in transfer film

**Fig. 5:**
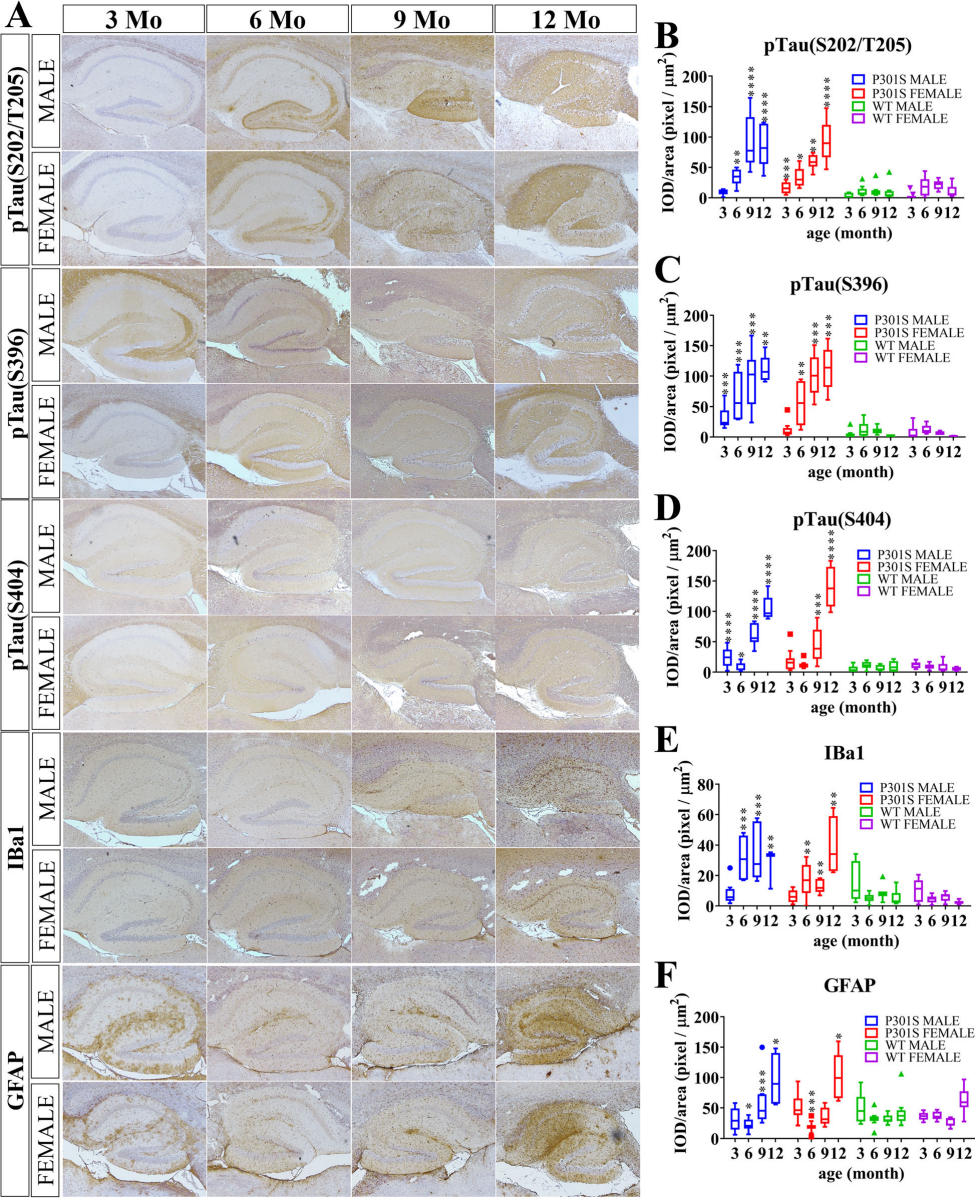
Quantification and statistical analysis of IHC positive mouse brain sections Mouse brain slides were stained via IHC with primary antibodies of anti-pTau(S202/T205) (AT8), anti-pTau(S396) (PHF 13), anti-pTau(S404), Iba1 (EPR16588) (to detect active microglia), and anti-GFAP (2E1.E9) (to detect astrocytes) are showed in (**a**). Positive areas in the hippocampus were quantified using Aperio ImageScope and are shown in **b**–**f**, respectively. Statistical analysis was performed between the sex- and age-matched P301S and WTs. *p < 0.05, **p < 0.01, ***p < 0.001, ****p < 0.0001

NFTs accumulated by hyperphosphorylated Tau proteins are the vital features of AD and related tauopathy. We measured the levels of NFT deposition and tau phosphorylation in the hippocampus by immunohistochemistry (IHC) and total brain homogenates by Western blot (WB) in P301S Tg mice and non-Tg littermates at different ages and sexes, respectively. Human tau in P301S Tg mice began to accumulate at 3 months old, and kept increasing until 12 months of age. Female Tg mice exhibited stronger expression than that of males in both soluble and insoluble fractions in total brain (Fig. 4j–l, Additional file 3). Immunoreactivity with AT8 antibody raised against tau phosphorylated at serine 202 and threonine 205 (pTauS202/T205) was only observed in the hippocampus of P301S mice at 6 months old in both sexes but not in WT controls (Fig. 5b). In male P301S Tg mice, tau phosphorylation levels at S202/T205 were enriched at 9 months of age and showed a slight decline at 12 months in the aqueous fraction. Instead, we detected a decline in 12-month-old mice in soluble RAB fraction (Fig. 4a), and an increase in both RIPA fraction (Fig. 4b) and insoluble urea fraction (Fig. 4c), indicating that tau proteins containing phosphorylation at Ser202/Thr205 formed insoluble NFTs. We noticed that the enrichment time of pTau(S202/T205) in IHC was earlier than that in WB, suggesting that this form of phosphorylated tau protein may develop earlier in the hippocampus than in other brain regions. We also noticed that the concentration of pTau(S202/T205) in male P301S mice was higher than that in female Tg mice, indicating the more severe dementia in male P301S Tg mice (Fig. 4a–c). Similar changes were found in tau phosphorylation levels at the S396 (pTauS396) and S404 (pTauS404). IHC staining results showed an age-dependent increase at the S396 and S404 phosphorylated points across the observation period (Fig. 5c, d) and more substantial increase in female Tg mice than in males. Western blot with the PHF13 antibody and polyclonal pTau(S404) antibody revealed that the level of phosphorylated tau at S396 and S404 increased to nearly the highest concentration in both RAB and RIPA soluble fraction of brain homogenates at the age of 6 months and 3 months, respectively (Fig. 4d–f, g–i). Furthermore, female Tg mice showed a faster decrease than that of males in both the level of pTau(S396) and pTau(S404) in brain homogenates just after 6 months in aqueous fractions, suggesting that more hyperphosphorylated tau proteins with pTau(S396) and pTau(S404) transferred into insoluble fractions in female P301S Tg mice (Fig. 4d–f, g–i). We also observed that the enrichment time of pTau(S404) in WB was earlier than that in IHC, indicating that this form of phosphorylated Tau protein may be extensively expressed in various brain regions. Consistent with the variations in phosphorylated tau protein in Tg mice of different sexes, the amount of neurons exhibited a significant decline from the age of 6 months in male P301S mice, but only a small decrease in females was observed until 12 months old, demonstrating a more toxic effect of pTau(S202/T205) on neurons (Fig. 4o). In sum, phosphorylated tau protein showed different degrees of abundance in male and female P301S Tg mice. pTau(S202/T205) may play a key role in NFT formation in male mice, while pTau(S404) is likely to exert a dominant function in NFT formation in female mice.

Microglia and astrocytes maintain homeostasis in the central nervous system by engulfment and degradation of extracellular material via phagocytosis [24]. We assessed the activation of microglia and astrocytes in the brains of P301S mice and WT littermates at different ages and sex. In the hippocampus of both male and female P301S mice, the amount of microglia kept increasing from the age of 3 months to 12 months but was maintained at a low level in WT littermates (Fig. 5e). Nevertheless, active microglia in the brain homogenates of WT mice appeared higher than that in P301S mice, which only showed a slight increase with aging (Fig. 4m). This implied that the microglia in the whole brain of P301S mice were in a quiescent or inhibited condition before typical tau pathology, and accumulated and converted to an active state in the hippocampal region induced by tau hyper-phosphorylation.

In addition, both IHC and WB for GFAP, a marker for astrocytes, revealed that GFAP was already activated in the hippocampus of 3-month-old P301S mice of both sexes (Figs. 5f and 4n). However, the astrocyte burden sharply declined at the age of 6 months, and then gradually increased, suggesting a significant induction of astrogliosis in P301S mice after tau phosphorylation. We noticed that the increase in astrocyte burden in the brains of male P301S mice was significantly higher than that in female Tg mice, possibly due to the higher level of hyper-phosphorylated tau in the brains of male Tg mice.

### Absence of MIP-3α and reduced sex-specific cytokines in P301S mouse plasma

**Fig. 6:**
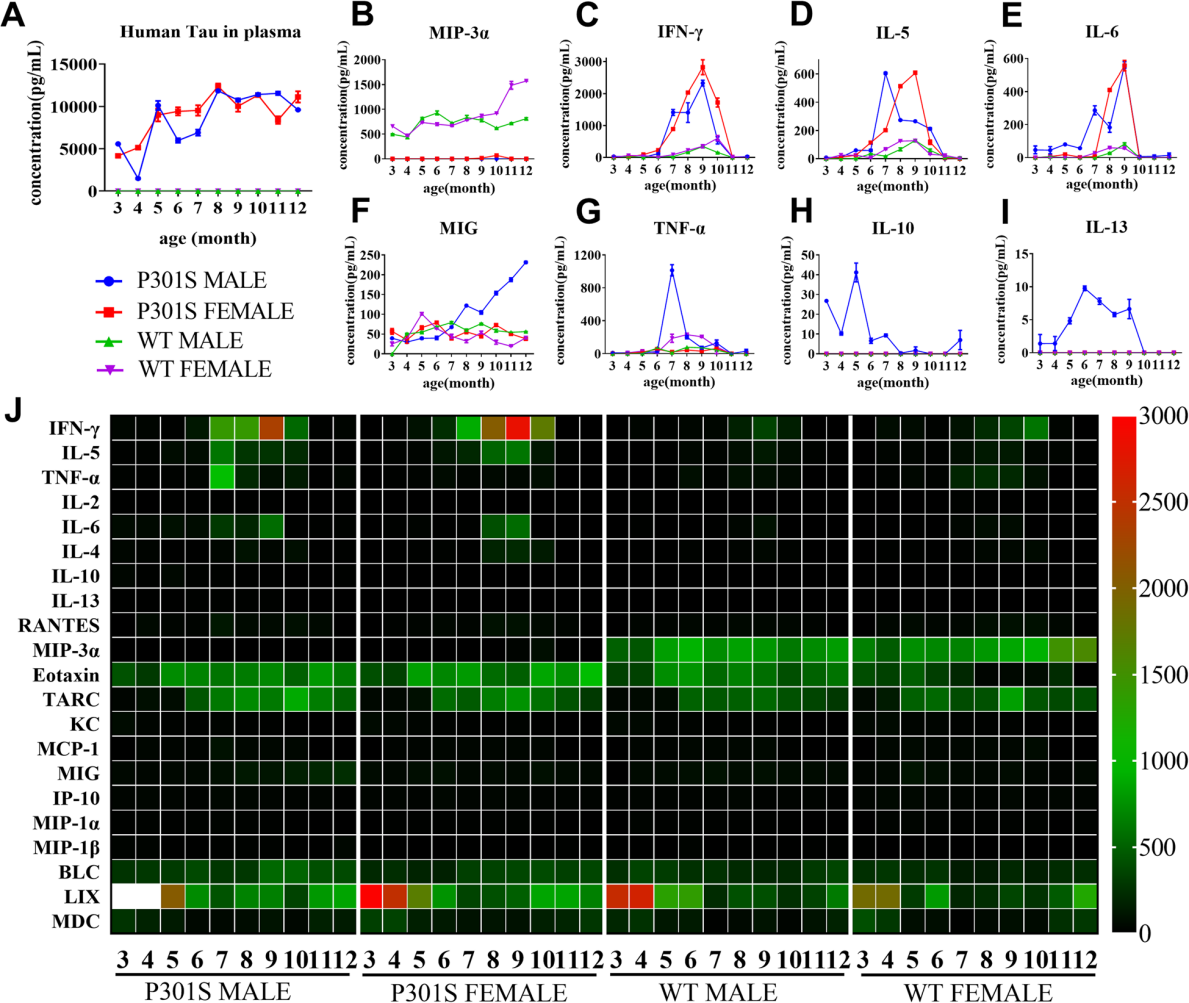
Changes in human total tau, inflammatory cytokines, and chemokines in mouse plasma. **a** Shows the concentration changes in human total Tau in plasma of four groups of mice between 3 and 12 months old. Line graph shows in mean ± S.E.M. **b**–**i** Show the concentration changes of MIP-3α, IFN-γ, IL-5, IL-6, MIG, TNF-α, IL-10, and IL-13 in RAB fraction of brain homogenates in the four groups. Data are presented as mean ± S.E.M. **j** Shows the concentration changes in plasma of the 21 factors listed in the left column of the heat map with increasing age of the four groups of mice. The unit of the legend shown on the right of the heat map is pg/mL. The two white squares show the concentration of LIX in the 3 and 4 months old P301S male mice exceeding 3000 pg/mL. The average concentrations in 3 and 4 months old of LIX were 3987.78 pg/mL and 3782.48 pg/mL, respectively

We examined whether sex differences influenced specific plasma and brain proteins in P301S Tg mice. We first checked the level of human tau in the plasma of different cohorts of mice. No human tau protein was detected in the plasma of WT mice. In the plasma of male and female P301S mice, plasma tau exhibited a tendency to increase between the age of 3 months and 8 months and was maintained at a stable and high level after 9 months, indicating early cerebrovascular lesions before pathologic changes of Tau in the brain, and potential leakage of anomalous tau protein may have engaged changes in the peripheral nervous system and immune system (Fig. 6a).

We then analyzed the concentration of inflammatory cytokines and chemokines in the plasma and soluble fractions of brain homogenates of P301S mice and WT littermates at different ages and sexes. As shown in the heat map Fig. 6j, no MIP-3α (also termed CCL20) was detected in the plasma of both male and female P301S mice entire life, while this protein showed an increasing tendency in WT mice across all ages (Fig. 6b). However, there was no significant difference in the concentration of brain homogenate MIP-3α between Tg and WTs (Additional file 1: Figure S12). The concentration of plasma IFN-γ, IL-5, and IL-6 in P301S mice were higher than that in sex- or age-matched WT littermates during the ages of 6 months to 10 months (Fig. 6c–e), which showed a synchronous accumulation with hyper-phosphorylated tau protein in the brain. Hence, systematic inflammation in the peripheral circulation such as the increase in IFN-γ, IL-5, and IL-6 may be a signal of abnormal phosphorylation of tau protein in P301S mice, and may be a subsequent event during pathological progression of tau. In addition, the continuous increase in the concentration of MIG (monokine induced by IFN-γ, also termed CXCL9) only occurred in male P301S male mice, whereas the concentration of MIG in female P301S mice maintained a low and steady tendency similar to that of WT mice (Fig. 6f). We also detected a higher level of TNF-α in the plasma of 7-month-old male P301S mice (Fig. 6g). Analogous abnormal levels were also observed with IL-10 and IL-13, which were only expressed in male P301S mice (Fig. 6h, i). In the soluble brain homogenates of these mice cohorts, no obvious differences in the concentration of these factors were found except IL-6, which exhibited a lower level in P301S mice and implied astrocytic malfunction (Additional file 1: Figure S12). Concentration of other inflammatory cytokines and chemokines in mouse plasma are showed in Additional file 1: Figure S13.

### Correlation analysis among pathological, ethological, and inflammatory factors

**Fig. 7:**
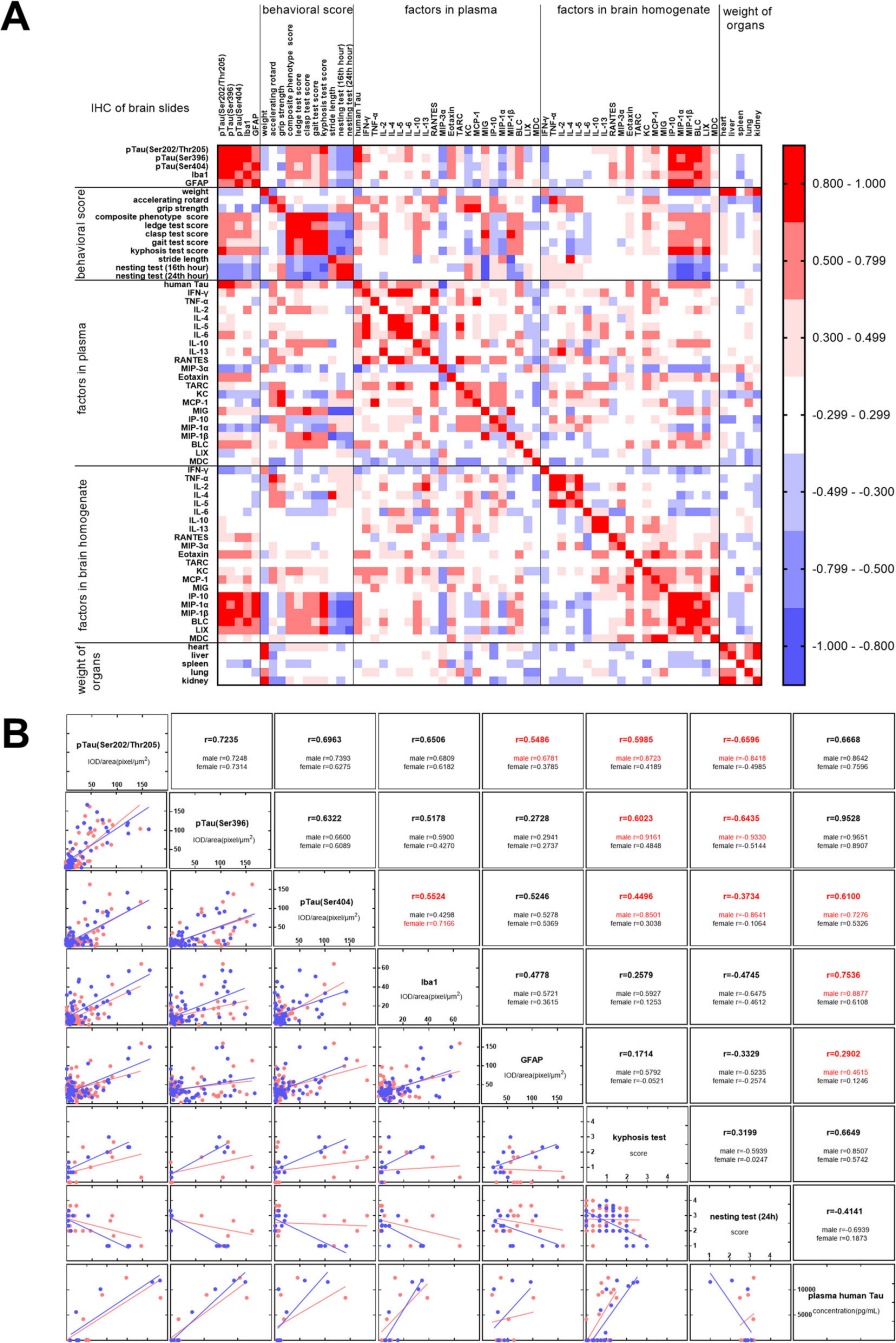
Correlation analysis among pathological, ethological, and inflammatory factors. **a** Shows the coefficient of Pearson correlation analysis between all detected factors. The average of factors in P301S male, P301S female, WT male, and WT female at each time points were applied for calculation. Both *r* > 0.8 or *r* < − 0.8 were taken as high relativity. **b** Shows the results of Pearson correlation coefficient calculated individually. In the scatter diagram, the blue point represents males and red represents females. Because the concentration of plasma human total tau was detected using a mixed sample, we still calculated the correlation coefficients with average data of all parameters compared to other factors

Based on the above findings, we aimed figure to elucidate a potential ethological index and specific disease-related plasma inflammatory factors to track disease progression during preclinical treatment of P301S mice. We included the average of all behavioral, pathological, and serological data of each subgroup of mice with the same sex and genotype during the observation period, and then calculated the Pearson correlations of all parameters (Fig. 7a). Some factors of all the detected mice were selected to calculate the Pearson correlation one-on-one (Fig. 7b).

Levels of tau phosphorylation are widely used as the golden standard for the identification of tau pathological progression, so we first analyzed the correlations between tau phosphorylation and other factors. The load of various phosphorylated tau protein in the hippocampus showed high correlations with each other, with Pearson correlation coefficients (*r*) of 0.938, 0.875, and 0.828 among pTau(S202/T205)-pTau(S396), pTau(S202/T205)-pTau(S404), and pTau(S404)-pTau(S396), respectively. Furthermore, the relevance of these phosphorylated tau proteins were not different between males and females. The level of activated microglia (detected by Iba1 antibody) in the hippocampus was more relevant to pTau(S202/T205) (*r* = 0.828) and pTau(S396) (*r* = 0.831) and less relevant to pTau(S404) (*r* = 0.728), but the level of astrocytes (detected by anti-GFAP antibody) in the hippocampus were more relevant to pTau(S404) (*r* = 0.844) than to pTau(S202/T205) (*r* = 0.651) and pTau(S396) (*r* = 0.517). When we calculated the Pearson correlation individually, a weaker relationship between microglia and pTau(S404), or astrocytes and pTau(S202/T205) was observed, possibly due to sex-induced differences.

The level of human tau proteins in plasma is widely use as biomarker to reflect the progression of tau pathology. In our experiment, it indeed showed a high correlation with phosphorylated Tau in the hippocampus detected by AT8 (*r* = 0.843) and PHF13 (*r* = 0.931), but had a low correlation with pTau(S404) (*r* = 0.614), which also varied with sex. A similar tendency was observed between human tau proteins in plasma and the level of microglia (*r* = 0.77) or astrocytes (*r* = 0.304) in the hippocampus, and male mice showed better responses to these factors.

The level of pTau(S202/T205) and pTau(S396) was also related to kyphosis score (*r* = 0.824 and *r* = 0.784, respectively) and score of nesting test (*r* = − 0.656 and *r* = − 0.522, respectively). They both exhibited a strong relationship only in male mice with all detected phosphorylation points of tau protein. However, for other behavioral experiments including grip strength test, stride length test, or accelerating rotarod test, they all exhibited low correlations with tau hyper-phosphorylation, which may be the reason for which those behavioral tests are not applicable for the assessment of tau P301S transgenic mice. In addition, the concentration of pTau(S202/T205), pTau(S396), and pTau(S404) were also strongly related to the level of brain IP-10 (*r* = 0.918, *r* = 0.824, and *r* = 0.945, respectively), MIP-1α (*r* = 0.802, *r* = 0.772 and *r* = 0.760, respectively), MIP-1β (*r* = 0.887, *r* = 0.829 and *r* = 0.879, respectively), and BLC (*r* = 0.894, *r* = 0.852 and *r* = 0.751, respectively), suggesting that IP-10, MIP-1α, MIP-1β, and LIX may participate in pathological progression of tau and impair neuronal function. Furthermore, we observed that the concentration of brain IP-10, MIP-1α, MIP-1β, and LIX were highly relevant to kyphosis score (*r* = 0.806, *r* = 0.920, *r* = 0.928 and *r* = 0.913, respectively), score of nesting test adjusted at the 24th hour (*r* = − 0.663, *r* = − 0.846, *r* = − 0.835, and *r* = − 0.801, respectively), and astrocytes (*r* = 0.843, *r* = 0.803, *r* = 0.802 and 0.705).

## Discussion

Accumulating studies have demonstrated that sex significantly influences AD pathology, behavioral status, plasma factor content, and therapeutic outcomes, suggesting more attention should be paid to the impact of sex differences on AD mice. In the present work, we have systematically investigated discrepancies in different sexes and age cohorts of tau P301S Tg mice in behavioral performance, tau pathology, and biomarkers in plasma and brain during AD progression, and also examined the correlations among pathological, ethological, and inflammatory factors.

In our study, male P301S mice showed an earlier and faster weight loss than that of female Tg mice, reflecting worse lesions in the physical function of male Tg mice. Similarly, male Tg mice exhibited a dramatically shortened survival rate than that of female mice, dependent to a large extent on greater weight loss and more comorbidities. In addition, our studies indicated that only male P301S mice but not female Tg mice exhibited decreased grip strength and shortened stride length at the late stage of the disease compared to WT littermates. However, the accelerating rotarod test can neither differentiate P301S Tg mice from WT mice, nor male and female Tg mice. Furthermore, the hind limb clasping and kyphosis test could effectively discriminate between tau P301S Tg mice and WT littermates.

Male P301S mice also exhibited an increased composite phenotype score that was significantly different from that of WT littermates. Thus, our studies demonstrated that only male P301S Tg mice were sensitive in weight change, survival rate, grip strength, stride length, clasping, kyphosis, and composite phenotype assessment.

Till now, there are no reports on sex- and age-related changes in memory and cognition in P301S Tg mice. Our studies showed that there was a significant difference in latency between female P301S mice and littermates by the age of 12 months, when some of the male Tg mice were unable to move in the MWM, demonstrating more severe dyskinesia in male P301S mice than in females, and a late memory deficit in this of tau mouse model. Due to the microtubule-related functions of tau, mutant tau-related transgenes mainly induce muscular atrophy and motor dysfunction but cause minor effects on memory ability. Consistently, we found that tau P301S Tg mice displayed sex differences in the OFT, and only female P301S mice displayed enhanced levels of locomotor activity in comparison to that of WT littermates. Furthermore, we observed that nest-building performance was a valid behavior measurement for TauP301S Tg mice. Compared to non-Tg littermates, male P301S Tg mice exhibited a gradually attenuated nest-building ability, while female P301S mice showed a steady nest-building capacity throughout disease progression. Although female P301S mice showed poorer memory dysfunction in the MWM, they did not perform more poorly in the nest-building test, and even exhibited more activity than male Tg mice in the OFT, suggesting that sex hormones have a complex role in progression of tauopathies.

As for typical pathological characteristics of P301S mice, tau proteins are hyperphosphorylated in the brain. We further analyzed the level of phosphorylated tau protein in total brain via WB, and also in regions relevant to cognition, such as the hippocampus, by IHC. We observed that the pTau(S404) first accumulated in the brains of P301S mice at 3 months of age, followed by Tau hyperphosphorylation on Ser396 at 6 months, followed by Ser202/Thr205 at 9 months. Soluble phosphorylated tau protein transferred into undissolved fractions which may constitute NFTs to different degrees, demonstrating that dysfunctions in memory, cognition, or anxiety in P301S mice are triggered by different states of phosphorylated tau protein. However, the various forms of phosphorylated tau in the hippocampus continuously increased from the age of 3 months in both male and female mice. Consistent with the upregulation of Tau phosphorylation, the number of neurons gradually declined with aging, demonstrating a toxic effect of tau phosphorylation on neurons and differences in pathology in male and female mice.

Next, we examined the sex- and age-related changes in microglia and astrocytes in P301S transgenic mice. In the hippocampus of P301S mice, active microglia and astrocytes accumulated as tau pathology was aggravated, while WT mice contained a low level of microglia in the hippocampus. Unexpectedly, we detected a significantly higher mount of microglia in brain homogenates in WTs and an abnormally lower level of microglia in P301S mice, although the amount of microglia in Tg mice brain typically increases with age. In contrast, unlike microglia, a high burden of astrocytes was detected in the hippocampus of P301S mice as early as 3 months with a sharp decline at 6 months followed by an increase. Several factors may stimulate the hyperproliferation of astrocytes in P301S mice, and the anomalous expression of angiopoietin-like 4 (ANGLP4) (an inhibitor of lipoprotein-lipase), chemokine CCL2, and abnormal iron transport in astrocytes may influence myelin debris clearance by microglia, as well as activation and function of microglia [25, 26]. Stimulating factors may be traced to very early periods just after birth. When mice reach 6 months old, the surge in phosphorylated tau cause microglial activation and decreased astrocytes, together with a nearly threefold increase in IL-6 in brain homogenates which reached the same levels as those of 6-month-old WTs, which then declined. The level of astrocytes reverted at the age of 9 months in P301S mice, especially in males, consistent with the decrease in IL-6 in brain homogenates in Tg male mice. Furthermore, the level of astrocytes in Tg female mice reverted at approximately the same time as that of age-matched female WTs, and active microglia increased faster than that in male Tg mice even though this was lower than that in WTs.

We also assessed the levels of specific plasma and brain factors in P301S Tg mice and their sex-related differences. Unexpectedly, MIP-3α was absent in the plasmas of P301S mice, while showed a gradual increase in WTs across all ages. MIP-3α is a chemokine for immature dendritic cells. The NF-κB pathway mediates the transcription of MIP-3α [27]. It has been reported that AD patients’ plasma can suppress the generation of CCL20 in SHSY-5Y cell lines [28]. This suggests that there factors may influence the NF-κB pathway and induce immunologic abnormalities in the peripheral system in the development of tau pathology. In addition, the increase in IFN-γ, IL-5, and IL-6 in the peripheral circulation only occurred in P301S Tg mice compared to that in WT littermates, which can be served as potential plasma biomarkers in P301S Tg mice. Consistent with the sex differences in behavior and neuropathology, several plasma factors including MIG, TNF-α, IL-10, and IL-13 exhibited specific changes in male P301S Tg mice compared to that in female Tg mice. Thus, the particular changes of specific inflammatory cytokines in P301S Tg mice revealed an abnormal immune environment in the peripheral circulatory system, which may impact brain homeostasis in tau Tg mice. These specific plasma proteins could also be potential biomarker for evaluation of tau-targeting therapeutic approaches.

Although we have confirmed the sexual dimorphism in the behavior, neuropathology, and biomarkers in tau P301S transgenic AD mice, we clearly realized that these results could not be simply expanded to other mutant tau Tg mice models. Obviously, the type of mutant tau expressed, the site on which the transgene is inserted together with the genetic background all have influences on the consequence of the behavioral analysis. Thus, when it comes to other tau transgenic mouse models including P301L, THY-Tau22, TauV337M, and TauR406W mouse models, the sex influence on the behavior, neuropathology, and biomarkers should be determined separately [29–32]. Recently, it has been reported that the P301S human 1N4R Tau isoform transgene is inserted into the Chromosome 3 of P301S Tg mice, excluding the influence of sex inheritance to our results [14]. In addition, the type of promoter used might also have an impact on the outcome of behavioral and neuropathological tests. Different promoter may possess different ability to turn on the gene expression in mice of different sex. For instance, for the 3×Tg-AD mice in which the APPswe and TauP301L gene are controlled by murine Thy1.2 promoter, the female mice displayed more neurofibrillary tangles than male, as well as amyloid plaque and neuroinflammation [30]. However, there are no obvious differences in a series of motor function and balance test between male and female 3×Tg-AD mice. Similarly, for the APP/PS1 mice in which the APP and PS1 mutant gene are drove by Prnp promoter, the female mice showed more Aβ plaque and phosphorylated tau protein burdens, more severe cerebral amyloid angiopathy, neuroinflammation, and gliosis [33]. Thus, the further investigation of sexual dimorphism in Tau 58/4 mice line might be necessary and interesting, in which the mutant tau transgene was expressed under the control of the murine Thy1.2 promoter [34].

## Conclusions

Our current systematic investigation revealed for the first time sex differences in the behavior, neuropathology, and plasma biomarkers in tau P301S Tg mice. Our results demonstrated that compared to female Tg mice, male P301S mice exhibited greater changes in weight loss, survival rate, grip strength test, stride length test, clasping, kyphosis, composite phenotype assessment, nest-building performance, tau phosphorylation at S202/T205, and astrocyte activation, which can be served as a more sensitive platform to assess tau-targeting therapeutic strategies for AD and related tauopathies. Our analysis also revealed that MIP-3α, IFN-γ, IL-5, and IL-6 can be served as potential plasma biomarkers for P301S Tg mice.

## Supplementary information

**Additional file 1: Figure S1.** Weight changes of mice. **Figure S2.** HE staining of lung. **Figure S3.** HE staining of spleen. **Figure S4.** HE staining of liver. **Figure S5.** HE staining of heart. **Figure S6.** HE staining of kidney. **Figure S7.** Composite phenotype scoring system test. **Figure S8.** Percent time in each quadrant in the MWM over 5 days. **Figure S9.** Latency and number of target platform crossings of four age groups of mice. **Figure S10.** Open field test. **Figure S11.** Nest building test. **Figure S12.** Concentrations of inflammatory cytokines and chemokines in RAB fraction of mouse brain homogenates. **Figure S13.** Concentration of inflammatory cytokines and chemokines in mouse plasma.

**Additional file 2:Table S1.** The mount of living mice and the events in each time point. **Table S2.** The data of P301S mice and WT mice in behavioral tests. **Table S3.** Behavioral tests for male and female P301S Tg mice and sex-matched WT littermates. **Table S4.** The latency and number of target platform crossings of P301S mice and WT mice in the MWM test. **Table S5.** The latency and number of target platform crossings of male and female P301S Tg mice and sex-matched WT littermates in MWM test.

**Additional file 3.** The original western blot figures of Fig. 4.

## Data Availability

The datasets used and/or analysis during the current study are available from the corresponding author on reasonable request.

## Abbreviations

AD: Alzheimer’s disease
Aβ: Amyloid-β
CBD: Corticobasal degeneration
ELISA: Enzyme-linked immunosorbent assay
FTLD: Frontotemporal lobar degeneration
H&E: Hematoxylin and eosin staining
IFN-γ: Interferon–γ
IHC: Immunohistochemistry
IL: Interleukin
IVC: Individually Ventilated Cages
MAPT: Microtubule-associated protein tau
MIP-3α: Macrophage inflammatory protein -3α
MWM: Morris Water Maze
NFTs: Neurofibrillary tangles
OFT: Open field test
PD: Pick’s disease
PHFs: Paired helical filaments
PSP: Progressive supranuclear palsy
RAB: Reassembly buffer
RIPA: Radio immunoprecipitation assay
Tg: Transgene or transgenic
WB: Western blot
WT: Wild type
