# Supplementary figures and images for: The behavioural and neuropathologic sexual dimorphism and absence of MIP-3α in tau P301S mouse model of Alzheimer’s disease
Source: J Neuroinflammation. 2020 Feb 24;17:72. doi: 10.1186/s12974-020-01749-w (PMC7041244; doi:10.1186/s12974-020-01749-w)

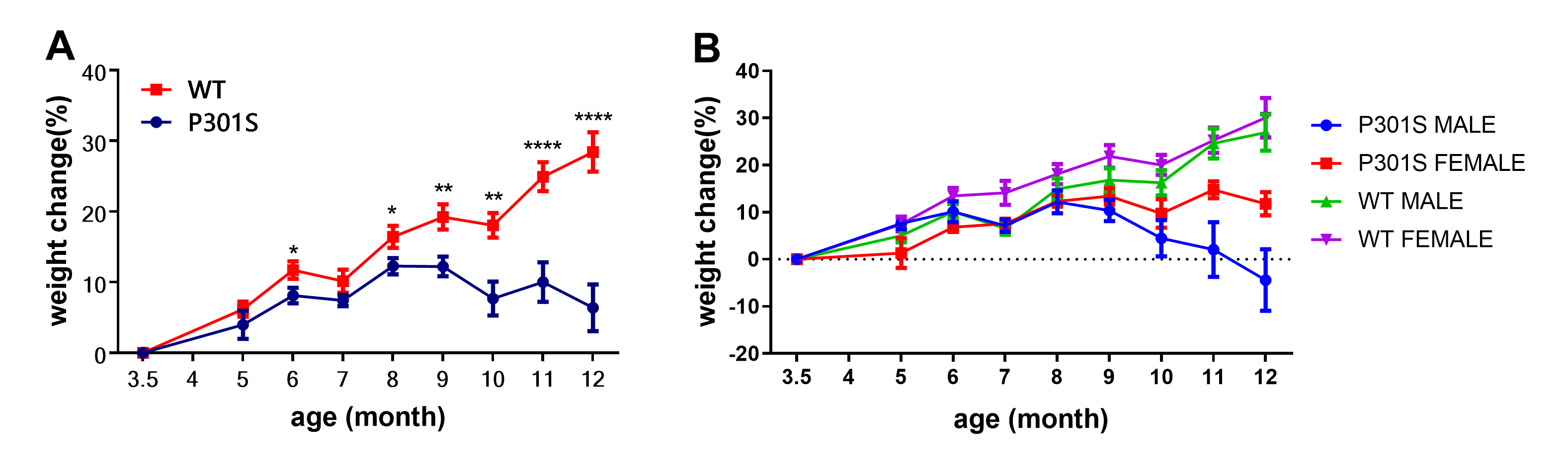

Supplement: Supplementary file 1 — Additional file 1: Figure S1. Weight changes of mice. Figure S2. HE staining of lung. Figure S3. HE staining of spleen. Figure S4. HE staining of liver. Figure S5. HE staining of heart. Figure S6. HE staining of kidney. Figure S7. Composite phenotype scoring system test. Figure S8. Percent time in each quadrant in the MWM over 5 days. Figure S9. Latency and number of target platform crossings of four age groups of mice. Figure S10. Open field test. Figure S11. Nest building test. Figure S12. Concentrations of inflammatory cytokines and chemokines in RAB fraction of mouse brain homogenates. Figure S13. Concentration of inflammatory cytokines and chemokines in mouse plasma. [file 12974_2020_1749_MOESM1_ESM.zip › Additional file 1-figure S1.tif]

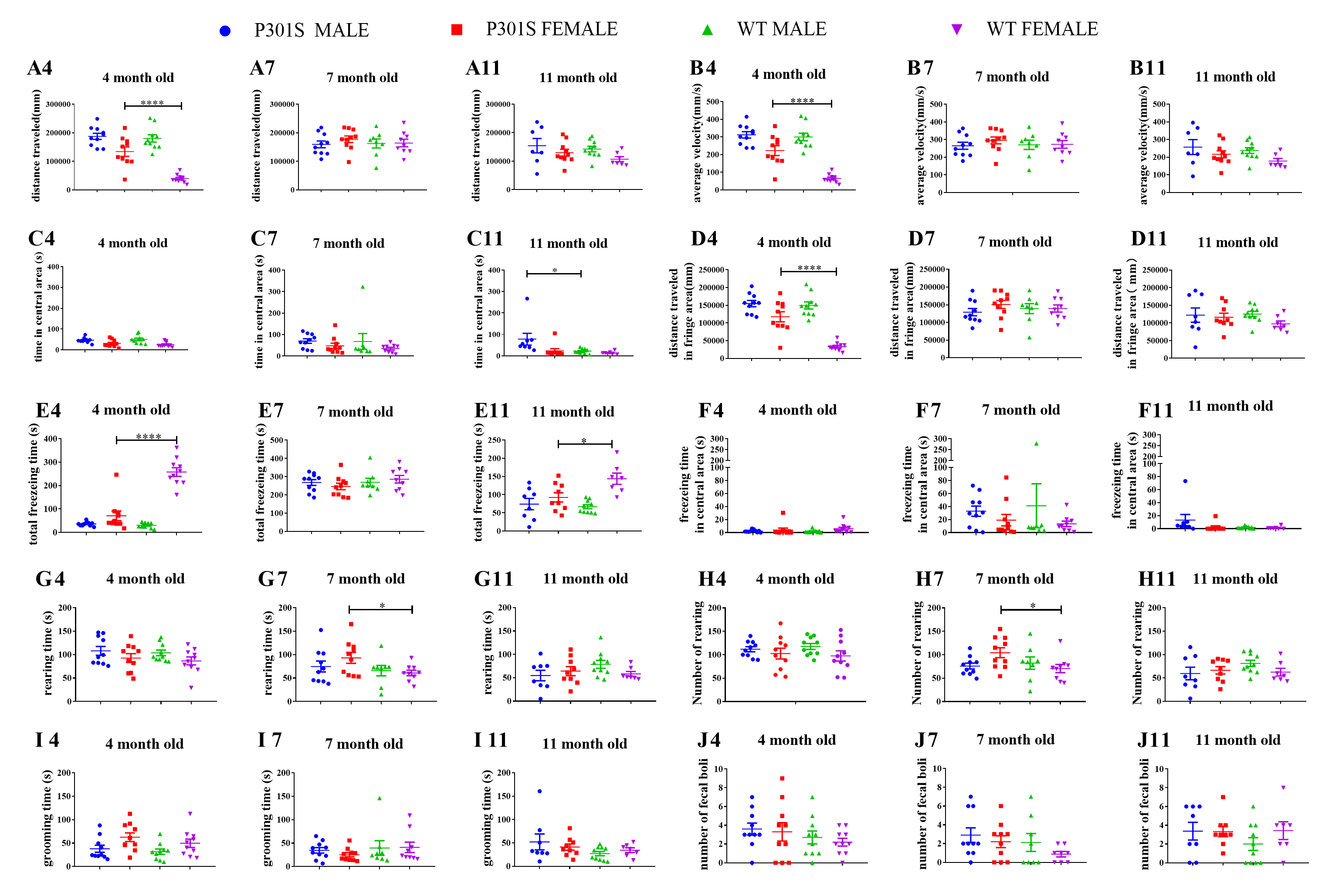

Supplement: Supplementary file 1 — Additional file 1: Figure S1. Weight changes of mice. Figure S2. HE staining of lung. Figure S3. HE staining of spleen. Figure S4. HE staining of liver. Figure S5. HE staining of heart. Figure S6. HE staining of kidney. Figure S7. Composite phenotype scoring system test. Figure S8. Percent time in each quadrant in the MWM over 5 days. Figure S9. Latency and number of target platform crossings of four age groups of mice. Figure S10. Open field test. Figure S11. Nest building test. Figure S12. Concentrations of inflammatory cytokines and chemokines in RAB fraction of mouse brain homogenates. Figure S13. Concentration of inflammatory cytokines and chemokines in mouse plasma. [file 12974_2020_1749_MOESM1_ESM.zip › Additional file 1-figure S10.tif]

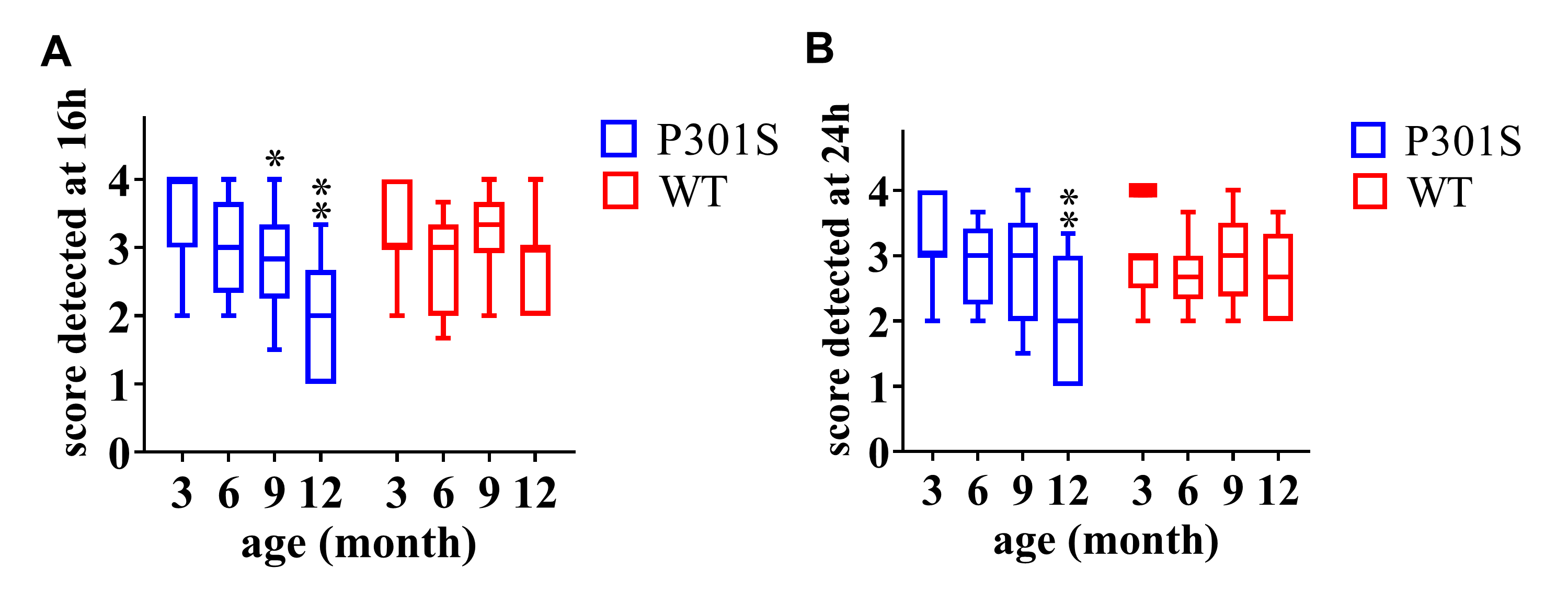

Supplement: Supplementary file 1 — Additional file 1: Figure S1. Weight changes of mice. Figure S2. HE staining of lung. Figure S3. HE staining of spleen. Figure S4. HE staining of liver. Figure S5. HE staining of heart. Figure S6. HE staining of kidney. Figure S7. Composite phenotype scoring system test. Figure S8. Percent time in each quadrant in the MWM over 5 days. Figure S9. Latency and number of target platform crossings of four age groups of mice. Figure S10. Open field test. Figure S11. Nest building test. Figure S12. Concentrations of inflammatory cytokines and chemokines in RAB fraction of mouse brain homogenates. Figure S13. Concentration of inflammatory cytokines and chemokines in mouse plasma. [file 12974_2020_1749_MOESM1_ESM.zip › Additional file 1-figure S11.tif]

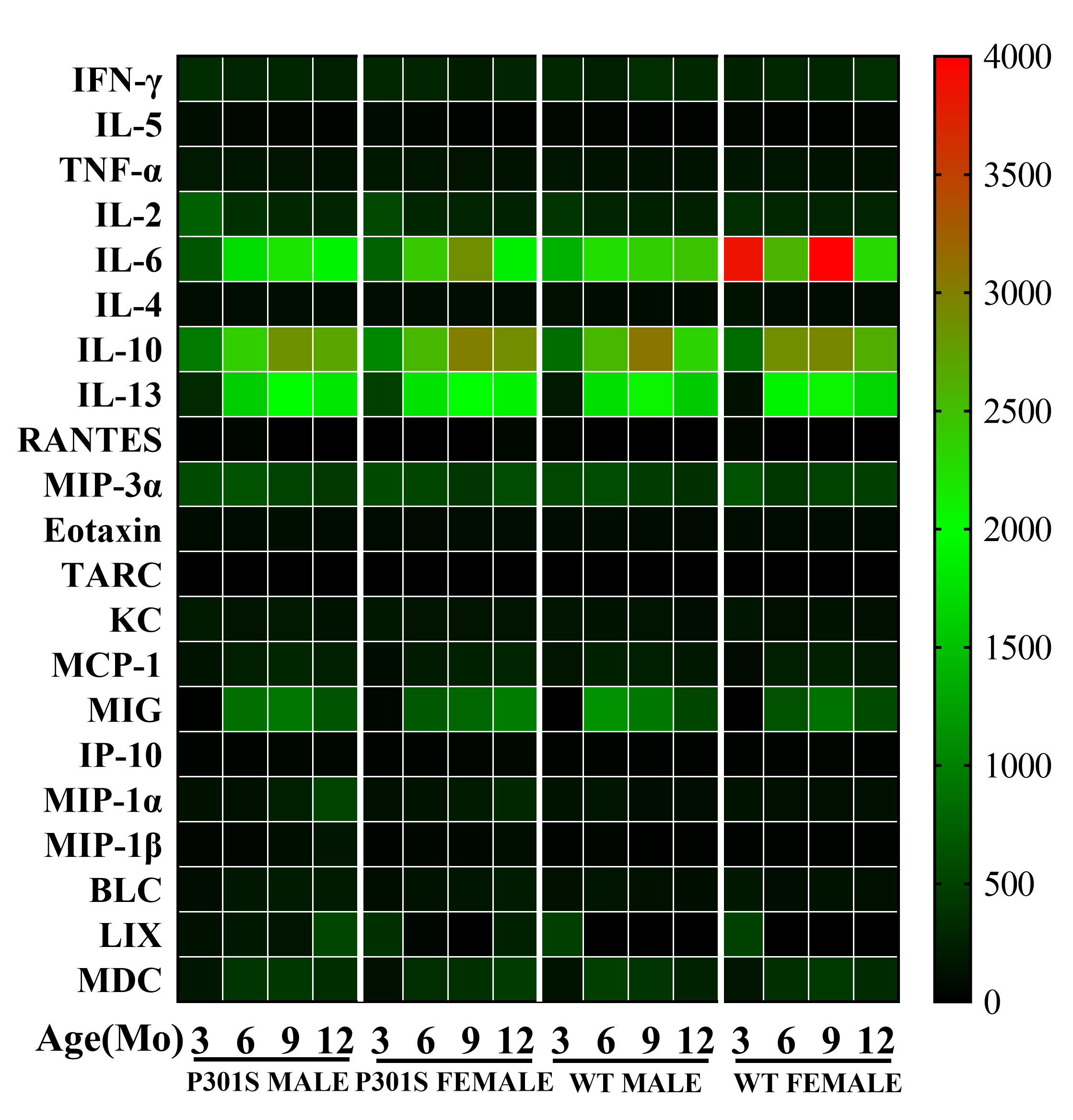

Supplement: Supplementary file 1 — Additional file 1: Figure S1. Weight changes of mice. Figure S2. HE staining of lung. Figure S3. HE staining of spleen. Figure S4. HE staining of liver. Figure S5. HE staining of heart. Figure S6. HE staining of kidney. Figure S7. Composite phenotype scoring system test. Figure S8. Percent time in each quadrant in the MWM over 5 days. Figure S9. Latency and number of target platform crossings of four age groups of mice. Figure S10. Open field test. Figure S11. Nest building test. Figure S12. Concentrations of inflammatory cytokines and chemokines in RAB fraction of mouse brain homogenates. Figure S13. Concentration of inflammatory cytokines and chemokines in mouse plasma. [file 12974_2020_1749_MOESM1_ESM.zip › Additional file 1-figure S12.tif]

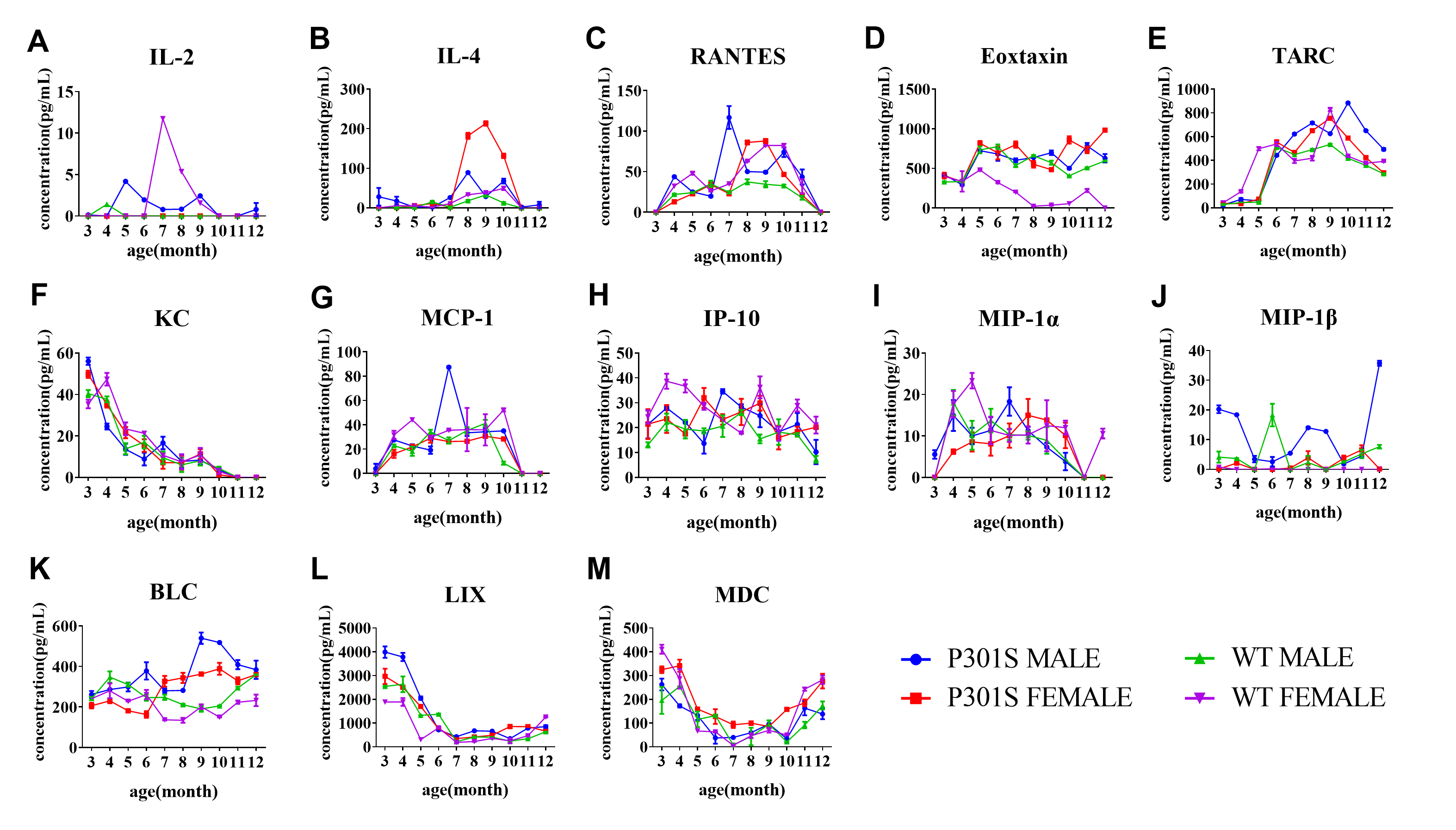

Supplement: Supplementary file 1 — Additional file 1: Figure S1. Weight changes of mice. Figure S2. HE staining of lung. Figure S3. HE staining of spleen. Figure S4. HE staining of liver. Figure S5. HE staining of heart. Figure S6. HE staining of kidney. Figure S7. Composite phenotype scoring system test. Figure S8. Percent time in each quadrant in the MWM over 5 days. Figure S9. Latency and number of target platform crossings of four age groups of mice. Figure S10. Open field test. Figure S11. Nest building test. Figure S12. Concentrations of inflammatory cytokines and chemokines in RAB fraction of mouse brain homogenates. Figure S13. Concentration of inflammatory cytokines and chemokines in mouse plasma. [file 12974_2020_1749_MOESM1_ESM.zip › Additional file 1-figure S13.tif]

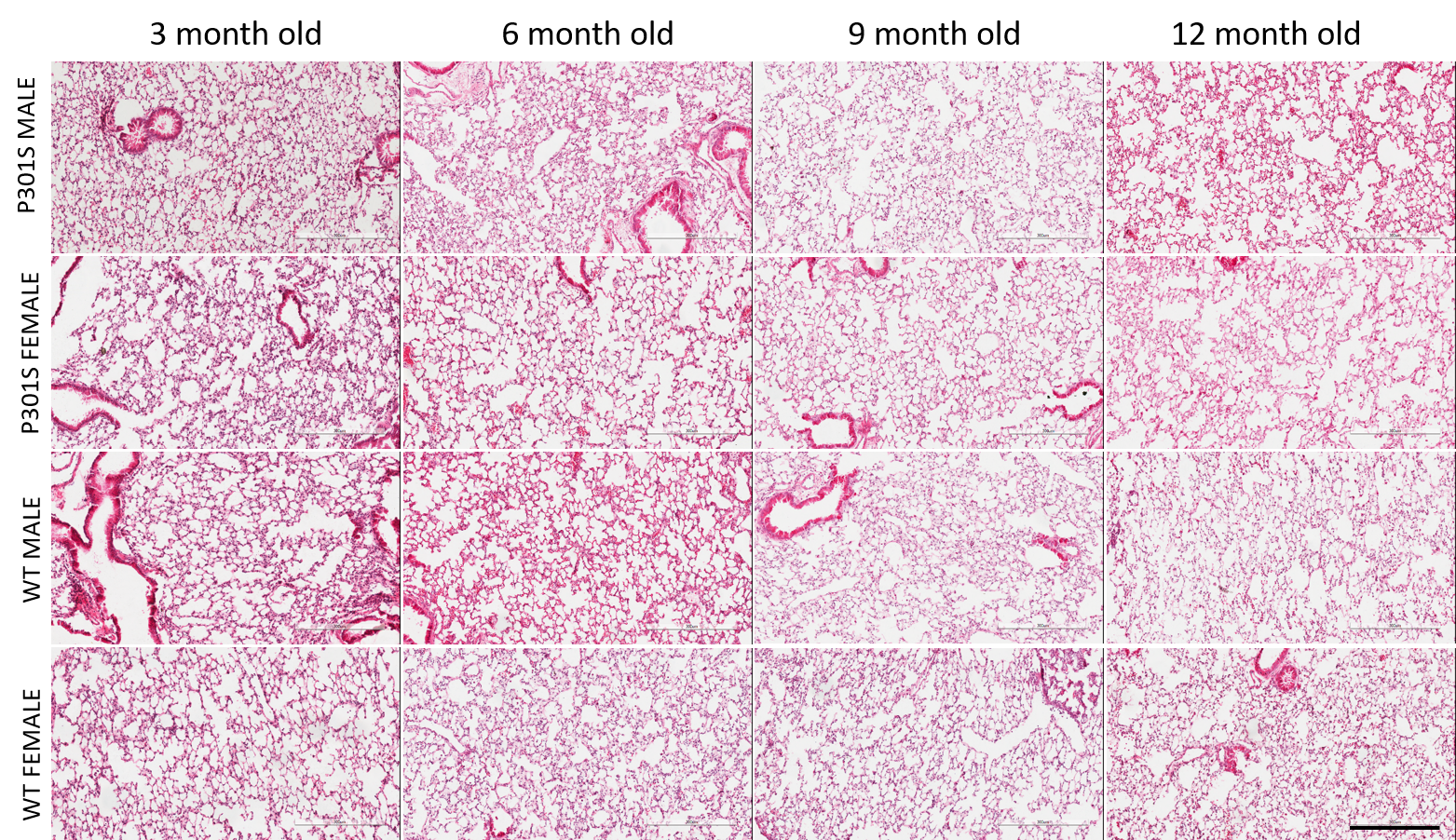

Supplement: Supplementary file 1 — Additional file 1: Figure S1. Weight changes of mice. Figure S2. HE staining of lung. Figure S3. HE staining of spleen. Figure S4. HE staining of liver. Figure S5. HE staining of heart. Figure S6. HE staining of kidney. Figure S7. Composite phenotype scoring system test. Figure S8. Percent time in each quadrant in the MWM over 5 days. Figure S9. Latency and number of target platform crossings of four age groups of mice. Figure S10. Open field test. Figure S11. Nest building test. Figure S12. Concentrations of inflammatory cytokines and chemokines in RAB fraction of mouse brain homogenates. Figure S13. Concentration of inflammatory cytokines and chemokines in mouse plasma. [file 12974_2020_1749_MOESM1_ESM.zip › Additional file 1-figure S2.tif]

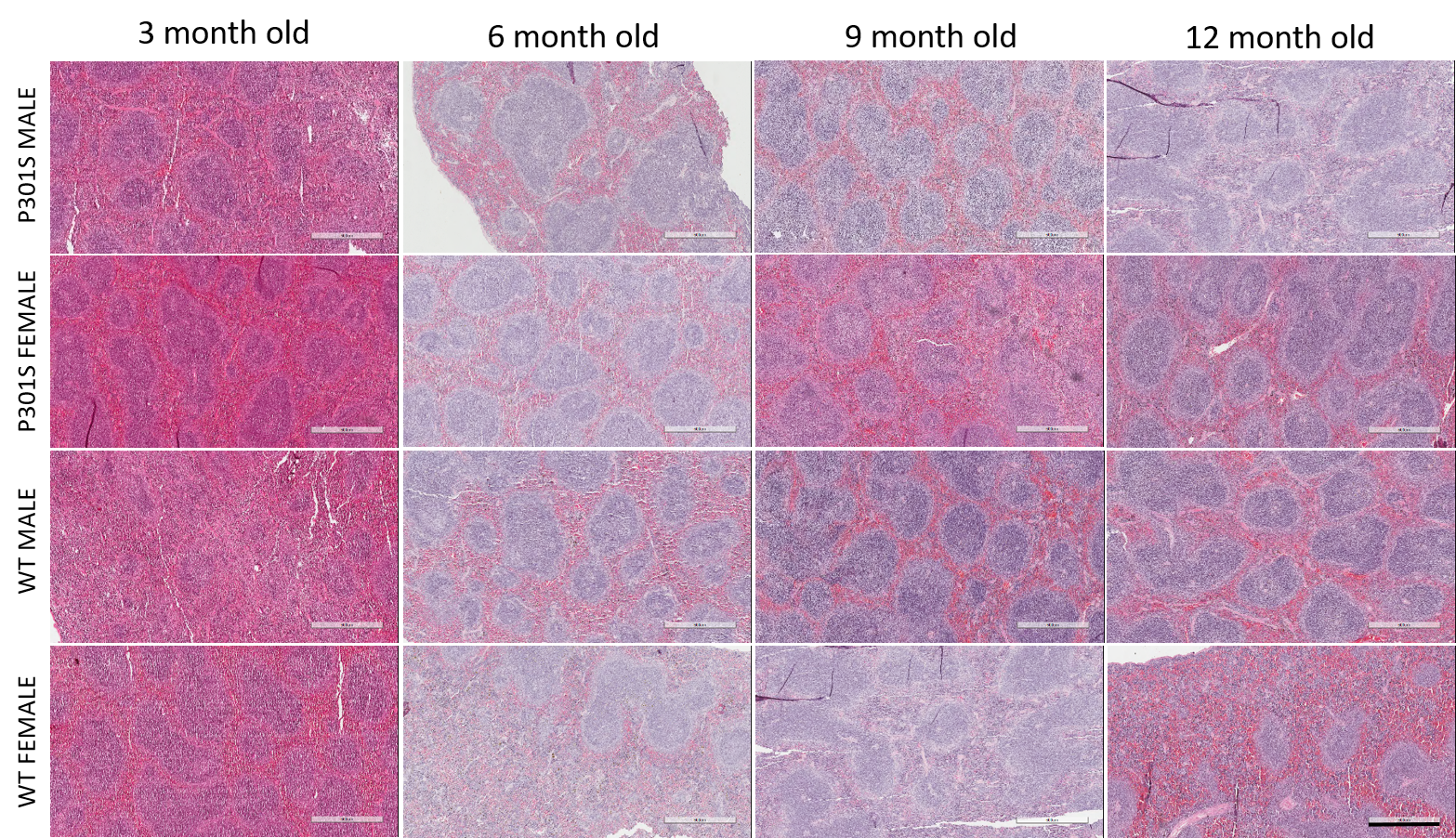

Supplement: Supplementary file 1 — Additional file 1: Figure S1. Weight changes of mice. Figure S2. HE staining of lung. Figure S3. HE staining of spleen. Figure S4. HE staining of liver. Figure S5. HE staining of heart. Figure S6. HE staining of kidney. Figure S7. Composite phenotype scoring system test. Figure S8. Percent time in each quadrant in the MWM over 5 days. Figure S9. Latency and number of target platform crossings of four age groups of mice. Figure S10. Open field test. Figure S11. Nest building test. Figure S12. Concentrations of inflammatory cytokines and chemokines in RAB fraction of mouse brain homogenates. Figure S13. Concentration of inflammatory cytokines and chemokines in mouse plasma. [file 12974_2020_1749_MOESM1_ESM.zip › Additional file 1-figure S3.tif]

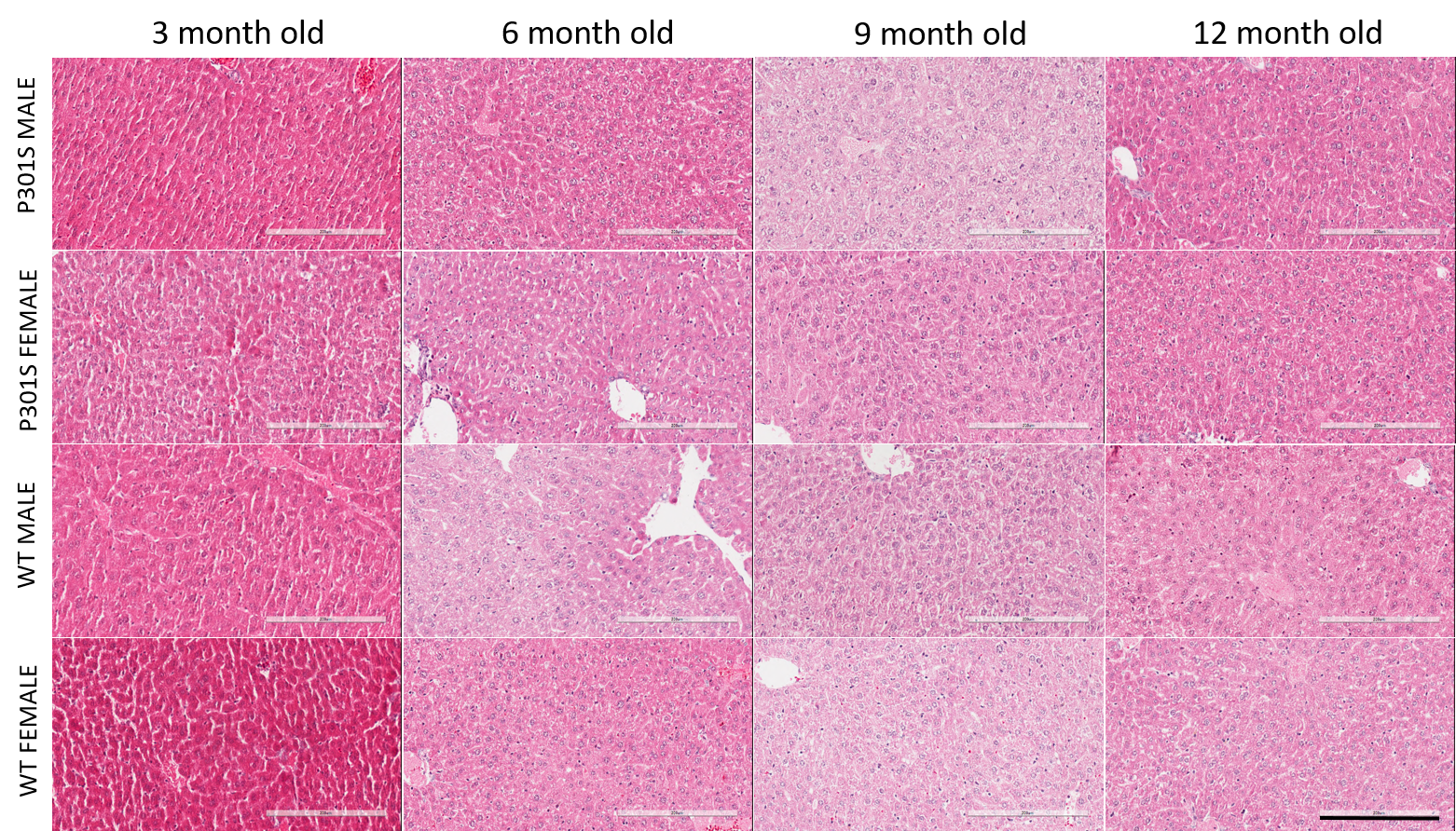

Supplement: Supplementary file 1 — Additional file 1: Figure S1. Weight changes of mice. Figure S2. HE staining of lung. Figure S3. HE staining of spleen. Figure S4. HE staining of liver. Figure S5. HE staining of heart. Figure S6. HE staining of kidney. Figure S7. Composite phenotype scoring system test. Figure S8. Percent time in each quadrant in the MWM over 5 days. Figure S9. Latency and number of target platform crossings of four age groups of mice. Figure S10. Open field test. Figure S11. Nest building test. Figure S12. Concentrations of inflammatory cytokines and chemokines in RAB fraction of mouse brain homogenates. Figure S13. Concentration of inflammatory cytokines and chemokines in mouse plasma. [file 12974_2020_1749_MOESM1_ESM.zip › Additional file 1-figure S4.tif]

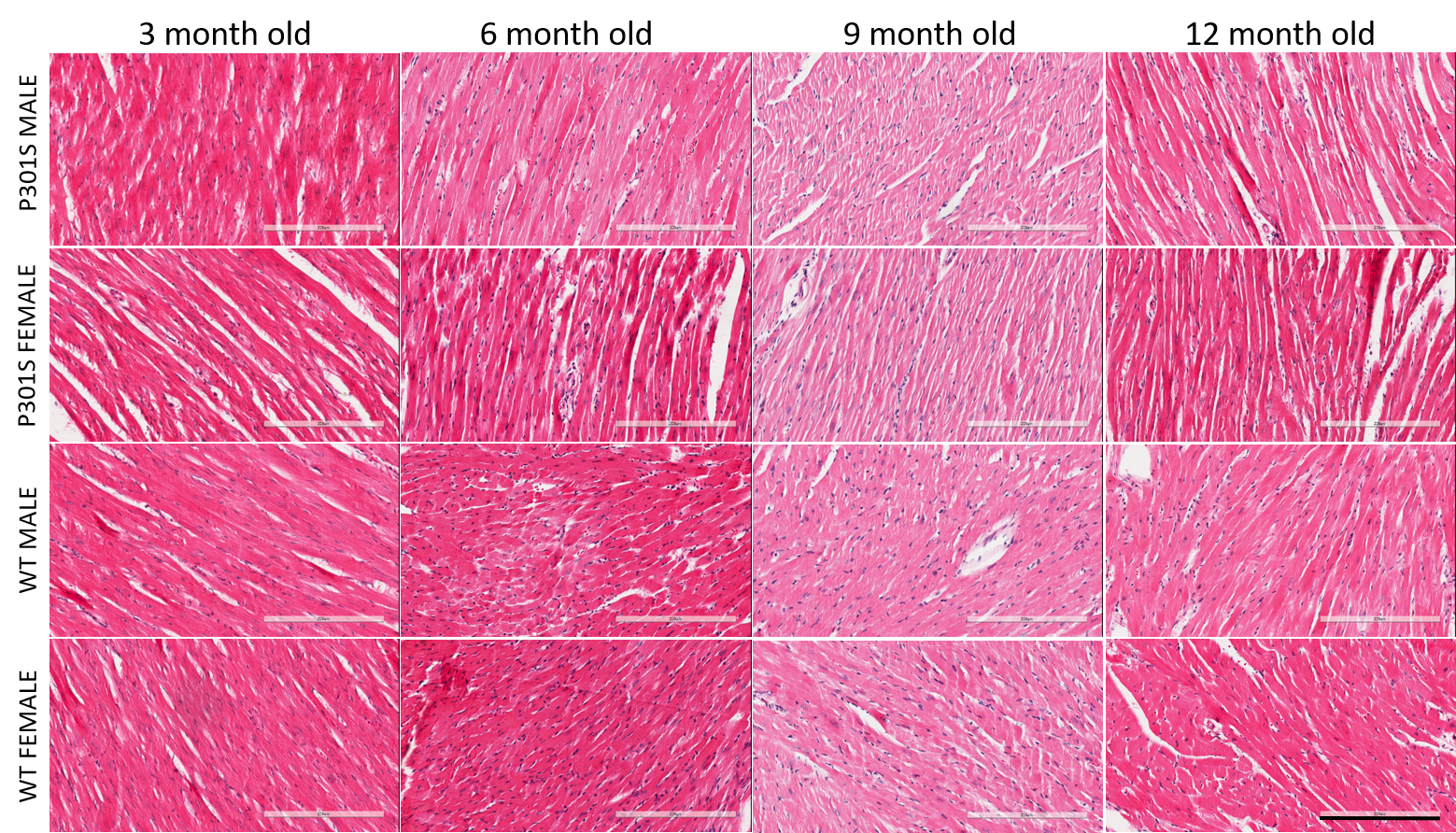

Supplement: Supplementary file 1 — Additional file 1: Figure S1. Weight changes of mice. Figure S2. HE staining of lung. Figure S3. HE staining of spleen. Figure S4. HE staining of liver. Figure S5. HE staining of heart. Figure S6. HE staining of kidney. Figure S7. Composite phenotype scoring system test. Figure S8. Percent time in each quadrant in the MWM over 5 days. Figure S9. Latency and number of target platform crossings of four age groups of mice. Figure S10. Open field test. Figure S11. Nest building test. Figure S12. Concentrations of inflammatory cytokines and chemokines in RAB fraction of mouse brain homogenates. Figure S13. Concentration of inflammatory cytokines and chemokines in mouse plasma. [file 12974_2020_1749_MOESM1_ESM.zip › Additional file 1-figure S5.tif]

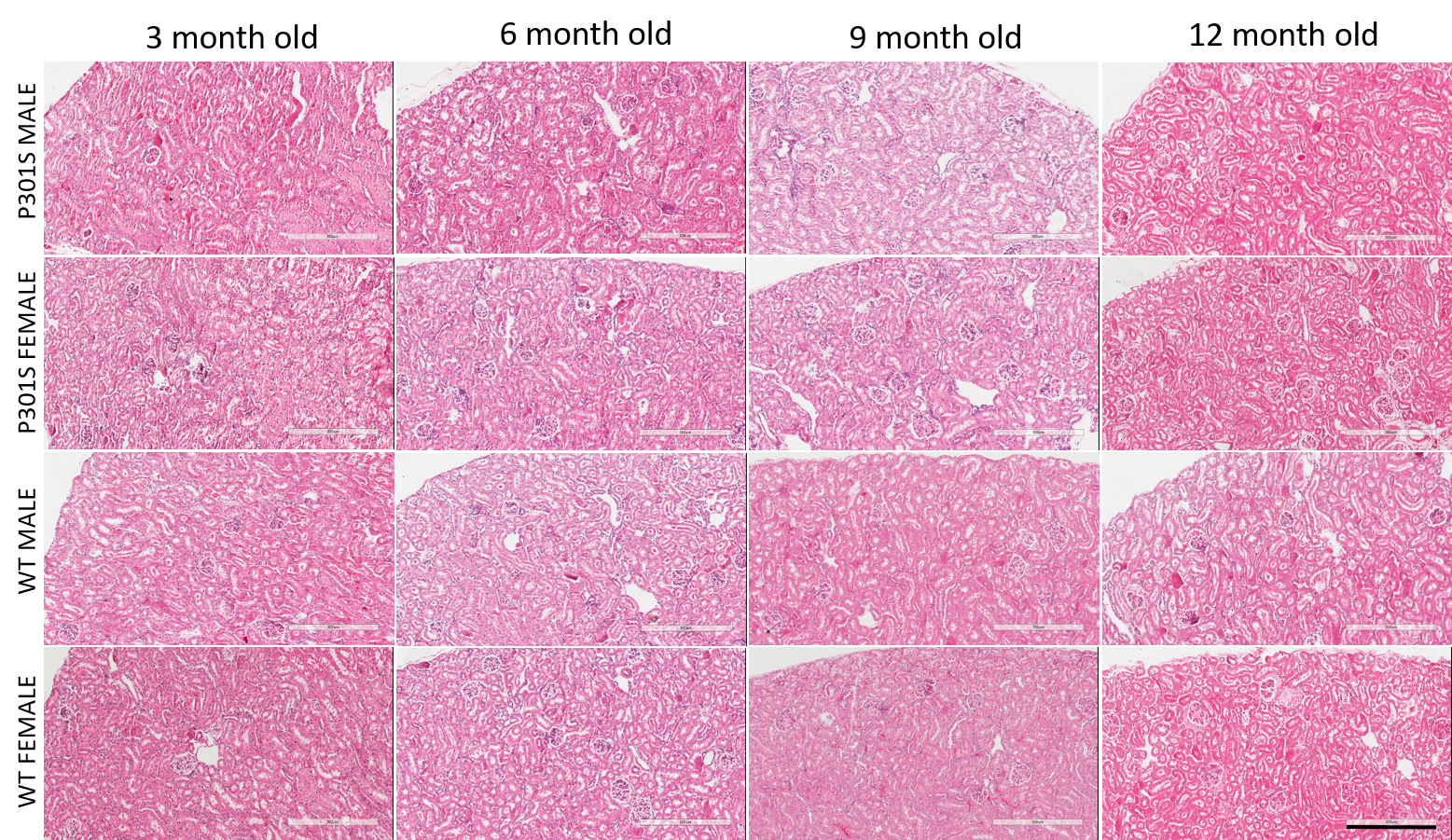

Supplement: Supplementary file 1 — Additional file 1: Figure S1. Weight changes of mice. Figure S2. HE staining of lung. Figure S3. HE staining of spleen. Figure S4. HE staining of liver. Figure S5. HE staining of heart. Figure S6. HE staining of kidney. Figure S7. Composite phenotype scoring system test. Figure S8. Percent time in each quadrant in the MWM over 5 days. Figure S9. Latency and number of target platform crossings of four age groups of mice. Figure S10. Open field test. Figure S11. Nest building test. Figure S12. Concentrations of inflammatory cytokines and chemokines in RAB fraction of mouse brain homogenates. Figure S13. Concentration of inflammatory cytokines and chemokines in mouse plasma. [file 12974_2020_1749_MOESM1_ESM.zip › Additional file 1-figure S6.tif]

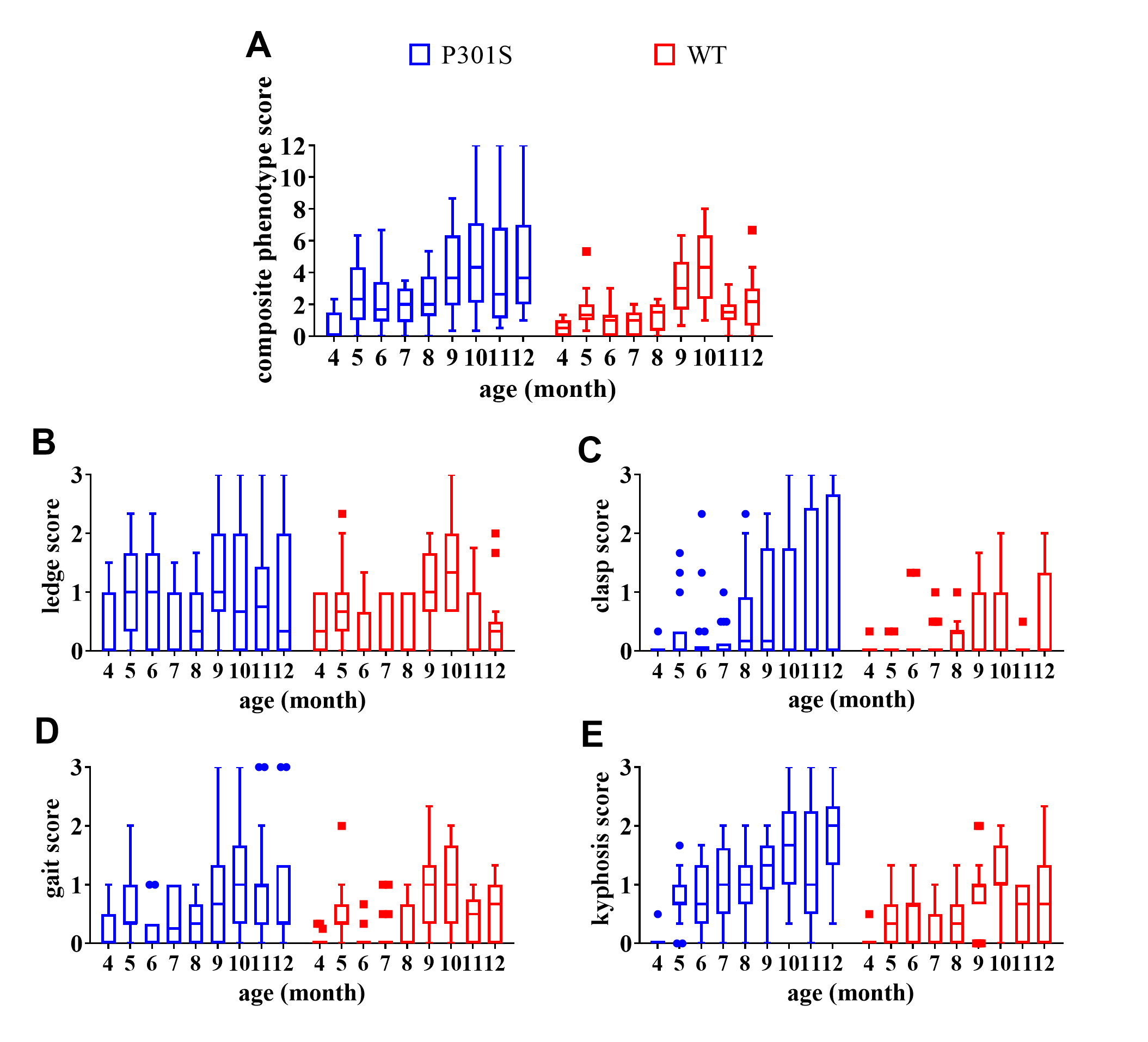

Supplement: Supplementary file 1 — Additional file 1: Figure S1. Weight changes of mice. Figure S2. HE staining of lung. Figure S3. HE staining of spleen. Figure S4. HE staining of liver. Figure S5. HE staining of heart. Figure S6. HE staining of kidney. Figure S7. Composite phenotype scoring system test. Figure S8. Percent time in each quadrant in the MWM over 5 days. Figure S9. Latency and number of target platform crossings of four age groups of mice. Figure S10. Open field test. Figure S11. Nest building test. Figure S12. Concentrations of inflammatory cytokines and chemokines in RAB fraction of mouse brain homogenates. Figure S13. Concentration of inflammatory cytokines and chemokines in mouse plasma. [file 12974_2020_1749_MOESM1_ESM.zip › Additional file 1-figure S7.tif]

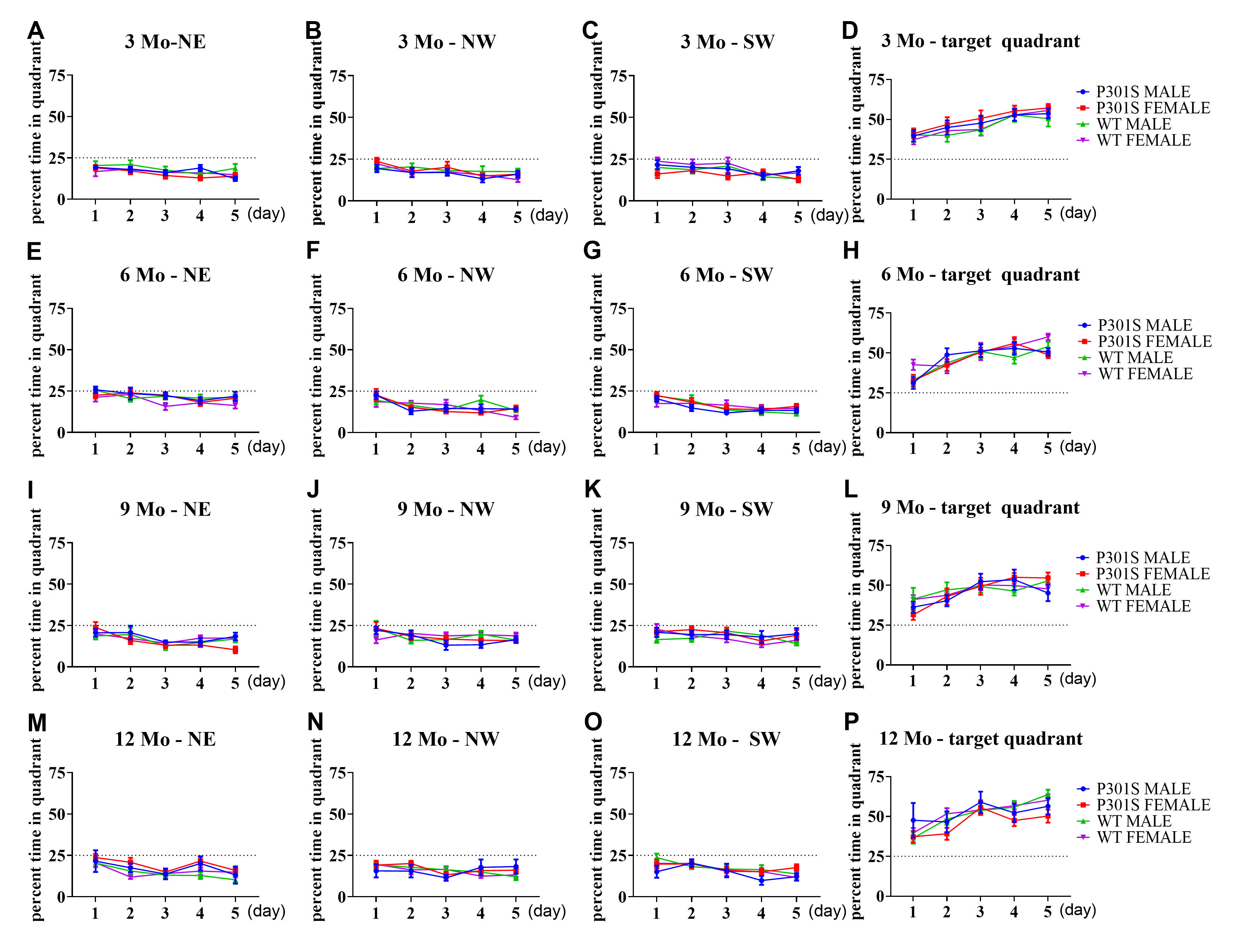

Supplement: Supplementary file 1 — Additional file 1: Figure S1. Weight changes of mice. Figure S2. HE staining of lung. Figure S3. HE staining of spleen. Figure S4. HE staining of liver. Figure S5. HE staining of heart. Figure S6. HE staining of kidney. Figure S7. Composite phenotype scoring system test. Figure S8. Percent time in each quadrant in the MWM over 5 days. Figure S9. Latency and number of target platform crossings of four age groups of mice. Figure S10. Open field test. Figure S11. Nest building test. Figure S12. Concentrations of inflammatory cytokines and chemokines in RAB fraction of mouse brain homogenates. Figure S13. Concentration of inflammatory cytokines and chemokines in mouse plasma. [file 12974_2020_1749_MOESM1_ESM.zip › Additional file 1-figure S8.tif]

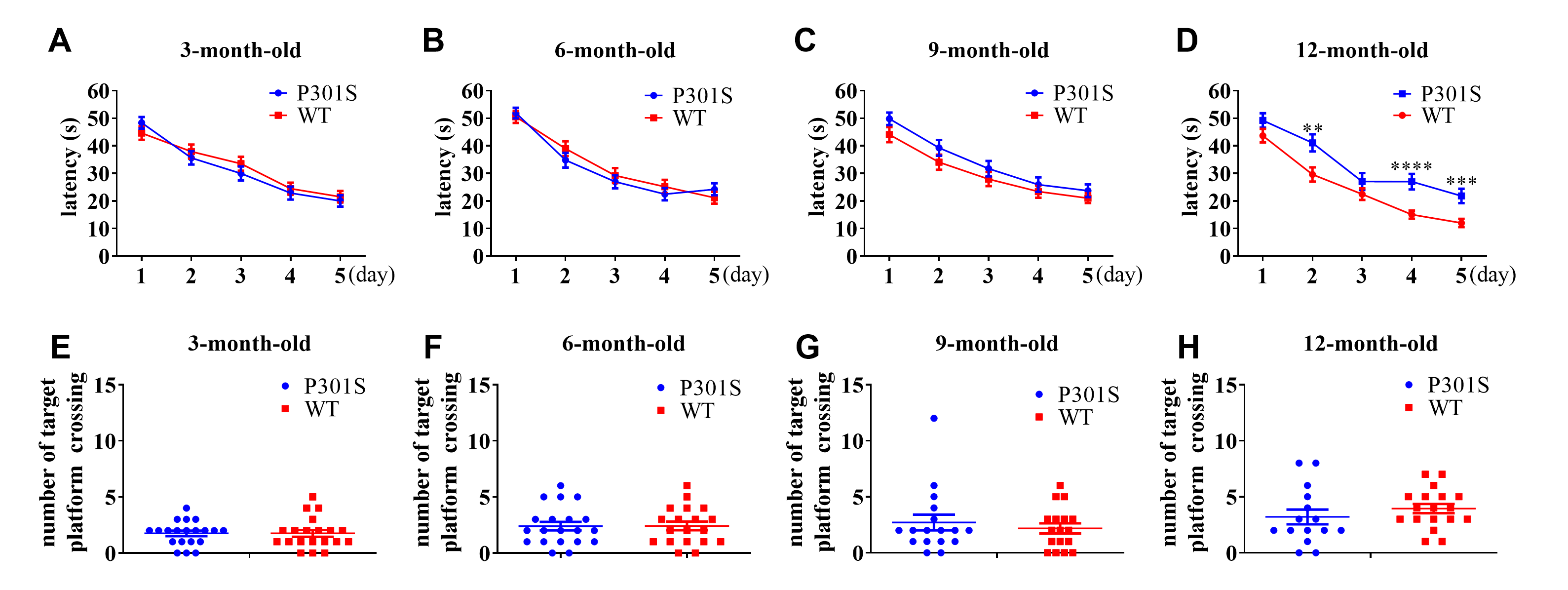

Supplement: Supplementary file 1 — Additional file 1: Figure S1. Weight changes of mice. Figure S2. HE staining of lung. Figure S3. HE staining of spleen. Figure S4. HE staining of liver. Figure S5. HE staining of heart. Figure S6. HE staining of kidney. Figure S7. Composite phenotype scoring system test. Figure S8. Percent time in each quadrant in the MWM over 5 days. Figure S9. Latency and number of target platform crossings of four age groups of mice. Figure S10. Open field test. Figure S11. Nest building test. Figure S12. Concentrations of inflammatory cytokines and chemokines in RAB fraction of mouse brain homogenates. Figure S13. Concentration of inflammatory cytokines and chemokines in mouse plasma. [file 12974_2020_1749_MOESM1_ESM.zip › Additional file 1-figure S9.tif]
