# Supplementary material for: The behavioural and neuropathologic sexual dimorphism and absence of MIP-3α in tau P301S mouse model of Alzheimer’s disease
Source: J Neuroinflammation. 2020 Feb 24;17:72. doi: 10.1186/s12974-020-01749-w (PMC7041244; doi:10.1186/s12974-020-01749-w)
Supplement: Supplementary file 2 — Additional file 2:Table S1. The mount of living mice and the events in each time point. Table S2. The data of P301S mice and WT mice in behavioral tests. Table S3. Behavioral tests for male and female P301S Tg mice and sex-matched WT littermates. Table S4. The latency and number of target platform crossings of P301S mice and WT mice in the MWM test. Table S5. The latency and number of target platform crossings of male and female P301S Tg mice and sex-matched WT littermates in MWM test. [file 12974_2020_1749_MOESM2_ESM.zip › Additional file 2-Table S1.docx]

Table S1. The mount of living mice and the events in each time point

| Alive mice  (events) | P301S male | P301S female | WT male | WT female |
| --- | --- | --- | --- | --- |
| 0-month-old | 39 | 40 | 40 | 40 |
| 1-month-old | 39 | 40 | 40 | 40 |
| 2-month-old | 39 | 40 | 40 | 40 |
| 3-month-old | 39  (**10 mice sacrificed**) | 40  (**10 mice sacrificed**) | 40  (**10 mice sacrificed**) | 40  (**10 mice sacrificed**) |
| 4-month-old | 28  (1 mouse died at the 146^th^ day) | 30 | 30 | 30 |
| 5-month-old | 27  (1 mouse died at the 169^th^ day) | 30 | 30 | 30 |
| 6-month-old | 27  (**8 mice sacrificed**) | 30  (**10 mice sacrificed**) | 30  (**10 mice sacrificed**) | 30  (**10 mice sacrificed**) |
| 7-month-old | 19 | 20 | 20 | 20 |
| 8-month-old | 19 | 20 | 20 | 20 |
| 9-month-old | 19  (**9 mice sacrificed**) | 20  (**10 mice sacrificed**) | 20  (**10 mice sacrificed**) | 20  (**10 mice sacrificed**) |
| 10-month-old | 10  (1 mouse died at the 320^th^ day) | 10  (1 mouse died at the 320^th^ day) | 10 | 10 |
| 11-month-old | 9  (1 mouse died at the 336^th^ day;  1 mouse died at the 360^th^ day;  2 mice died at the 364^th^ day) | 9 | 10 | 10 |
| 12-month-old | 5  (**5 mice sacrificed**) | 9  (**9 mice sacrificed**) | 10  (**10 mice sacrificed**) | 10  (**10 mice sacrificed**) |

Blood sample were collected from all the alive mice at each detected time point, mice were sacrificed for histological analysis and western blot of brain homogenate. Morris Water Maze were undergone before mice were sacrificed. Those mice survived to 10-month-old were served for monitoring the behavioral changes from the age of 3-12 month old, including body weight, grip force test, accelerating rotarod test, stride length, ledge test, hindlimb clasping, gait grade and kyphosis grade.
