# Supplementary material for: The behavioural and neuropathologic sexual dimorphism and absence of MIP-3α in tau P301S mouse model of Alzheimer’s disease
Source: J Neuroinflammation. 2020 Feb 24;17:72. doi: 10.1186/s12974-020-01749-w (PMC7041244; doi:10.1186/s12974-020-01749-w)
Supplement: Supplementary file 2 — Additional file 2:Table S1. The mount of living mice and the events in each time point. Table S2. The data of P301S mice and WT mice in behavioral tests. Table S3. Behavioral tests for male and female P301S Tg mice and sex-matched WT littermates. Table S4. The latency and number of target platform crossings of P301S mice and WT mice in the MWM test. Table S5. The latency and number of target platform crossings of male and female P301S Tg mice and sex-matched WT littermates in MWM test. [file 12974_2020_1749_MOESM2_ESM.zip › Additional file 2-Table S3.docx]

| Age(month) |  | 3.5 | 4 | 5 | 6 | 7 | 8 | 9 | 10 | 11 | 12 |
| --- | --- | --- | --- | --- | --- | --- | --- | --- | --- | --- | --- |
| **Weight(g)** | **WT male** | **27.38±0.52** |  | **28.75±0.66** | **30.15±0.79** | **29.17±0.65** | **31.45±0.84** | **31.99±0.98** | **31.77±0.73** | **34.07±0.95** | **34.69±1.07** |
|  | **P301S male** | **24.51±0.9****** |  | **26.13±1.04**** | **26.14±0.99***** | **25.49±1.11***** | **26.62±0.99***** | **26.18±0.9****** | **24.78±1.13****** | **24.25±1.17****** | **21.96±1.54****** |
|  | **WT female** | **21.47±0.39** |  | **23.06±0.38** | **24.33±0.35** | **24.47±0.54** | **25.34±0.52** | **26.18±0.71** | **25.77±0.6** | **26.91±0.76** | **27.92±1.03** |
|  | **P301S female** | **20.62±0.39** |  | **20.86±0.68*** | **22±0.29*** | **22.17±0.42*** | **23.15±0.42*** | **23.38±0.54*** | **22.63±0.75*** | **23.7±0.49**** | **23.08±0.59**** |
|  |  |  |  |  |  |  |  |  |  |  |  |
| **Weight gain ratio** | **WT male** | **0** |  | **5.04±1.43%** | **10.11±1.71%** | **6.57±1.35%** | **14.92±2.27%** | **16.83±2.5%** | **16.27±2.67%** | **24.6±3.17%** | **26.95±3.85%** |
|  | **P301S male** | **0** |  | **7.62±1.18%** | **10.11±2.23%** | **7.14±1.32%** | **12.18±2.43%** | **10.38±2.24%** | **4.47±3.87%*** | **2.08±5.79%****** | **-4.41±6.51%****** |
|  | **WT female** | **0** |  | **7.48±1.58%** | **13.47±1.69%** | **14.12±2.56%** | **18.1±2.14%** | **21.9±2.4%** | **20.03±2.13%** | **25.3±2.69%** | **30.06±4.2%** |
|  | **P301S female** | **0** |  | **1.33±3.17%** | **6.85±1.03%**** | **7.6±1.05%*** | **12.34±1.18%** | **13.41±1.74%*** | **9.74±3.02%*** | **14.78±1.79%*** | **11.79±2.48%**** |
|  |  |  |  |  |  |  |  |  |  |  |  |
| grip strength test(g) | WT male | 253.68±11.69 |  | 255.64±8.39 | 266.47±10.35 | -- | 261.05±4.93 | 263.76±7.04 | 186.93±8.08 | 227.9±11.17 | 231.38±10.8 |
|  | P301S male | 257.92±19.65 |  | 238.66±13.4 | 255.28±13.96 | -- | 249.19±8.09 | 252.24±9.77 | 208.68±26.12* | 204.83±23.12 | 144.14±45.4** |
|  | WT female | 252.61±13.48 |  | 258.64±10.02 | 271.46±16.03 | -- | 265.05±10.79 | 268.25±13.08 | 218.41±10.87 | 205.83±16.01 | 194.69±7.24 |
|  | P301S female | 277.8±13.77 |  | 241.58±9.29 | 268.68±5.85 | -- | 255.13±6.23 | 261.91±5.58 | 216.68±17.71 | 203.17±8.47 | 205.18±9.67 |
|  |  |  |  |  |  |  |  |  |  |  |  |
| Latency of accelerating rotarod test(s) | WT male | 413±67.27 |  | 152.3±25.72 | 164.4±50.4 | 124.1±13.73 | 95.5±8.6 | 86.6±11.39 | 95.4±10.53 | 92.1±9.69 | 80.6±11.27 |
|  | P301S male | 486.67±59.13 |  | 312.88±75.08 | 225.14±70.71 | 215.86±72.74 | 133.29±36.87 | 242.43±76.88 | 163.71±72.25 | 90.5±43.09 | 133.4±116.89 |
|  | WT female | 448.89±76.49 |  | 174.89±54.3 | 203±45.2 | 166.56±32.48 | 150.33±24.95 | 87.44±10.23 | 89.44±10.81 | 80.78±7.8 | 75.67±13.94 |
|  | P301S female | 311.73±55.29 |  | 208.7±40.54 | 207.45±41.88 | 170.91±33.32 | 182.18±27.6 | 209.73±56.23* | 146.45±49.27 | 165.2±44.51 | 136±37.18 |
|  |  |  |  |  |  |  |  |  |  |  |  |
| stride length(cm) | WT male | 5.91±0.12 |  | 5.06±0.14 | 5.17±0.14 | 5.18±0.18 | 5.55±0.17 | 5.36±0.16 | 5.22±0.32 | 4.91±0.29 | 5.21±0.3 |
|  | P301S male | 5.17±0.33 |  | 5.35±0.29 | 5.65±0.22 | 5.32±0.32 | 5.23±0.27 | 5.49±0.2 | 4.53±0.49 | 4.27±0.48 | 2.75±1.13* |
|  | WT female | 5.35±0.33 |  | 5.66±0.24 | 5.7±0.25 | 5.6±0.23 | 5.8±0.29 | 5.24±0.36 | 4.9±0.24 | 4.54±0.23 | 5.35±0.24 |
|  | P301S female | 5.2±0.25 |  | 5.45±0.23 | 5.21±0.23 | 5.58±0.2 | 5.72±0.34 | 5.74±0.16 | 4.94±0.58 | 5.84±0.24* | 5.52±0.31 |
|  |  |  |  |  |  |  |  |  |  |  |  |
| Total score of Composite Phenotype Scoring System | **WT male** |  | **0.62±0.17** | **1.97±0.43** | **1.3±0.26** | **0.65±0.18** | **1.32±0.31** | **3.67±0.54** | **4.8±0.71** | **1.44±0.23** | **2.73±0.51** |
|  | **P301S male** |  | **0.94±0.26*** | **4±0.68*** | **3.76±0.61****** | **2±0.48*** | **3.81±0.42**** | **6.48±0.61*** | **7.05±1.14** | **8.1±1.32****** | **8.73±1.58***** |
|  | WT female |  | 0.4±0.13 | 1.52±0.29 | 0.56±0.23 | 1±0.28 | 1.22±0.28 | 2.63±0.65 | 3.48±0.59 | 1.42±0.38 | 1.37±0.51 |
|  | P301S female |  | 0.48±0.27 | 1.76±0.36 | 1.09±0.21 | 1.95±0.37 | 1.48±0.27 | 2.67±0.47 | 3.52±0.99 | 1.75±0.27**** | 2.53±0.36 |
|  |  |  |  |  |  |  |  |  |  |  |  |
| Ledge score | WT male |  | 0.56±0.14 | 1±0.24 | 0.43±0.15 | 0.45±0.16 | 0.5±0.17 | 1.17±0.19 | 1.73±0.25 | 0.33±0.13 | 0.5±0.23 |
|  | P301S male |  | 0.65±0.19 | 1.46±0.25 | 1.76±0.19**** | 0.5±0.24 | 0.86±0.24 | 1.86±0.34 | 1.52±0.38 | 1.75±0.35**** | 1.93±0.61*** |
|  | WT female |  | 0.28±0.15 | 0.41±0.07 | 0.07±0.07 | 0.22±0.15 | 0.26±0.14 | 0.96±0.24 | 1.26±0.22 | 0.53±0.24 | 0.26±0.09 |
|  | P301S female |  | 0.27±0.14 | 0.67±0.16 | 0.45±0.16 | 0.27±0.16 | 0.3±0.12 | 0.97±0.24 | 0.82±0.35 | 0.33±0.17 | 0.43±0.22 |
|  |  |  |  |  |  |  |  |  |  |  |  |
| Clasp score | **WT male** |  | **0.03±0.03** | **0.07±0.04** | **0.27±0.18** | **0.1±0.07** | **0.28±0.1** | **0.6±0.25** | **0.87±0.24** | **0.05±0.05** | **0.67±0.28** |
|  | **P301S male** |  | **0±0** | **0.58±0.23**** | **0.57±0.35** | **0.21±0.15*** | **1.19±0.36****** | **1.86±0.18***** | **0.97±0.35** | **2.18±0.51****** | **2.4±0.44****** |
|  | WT female |  | 0±0 | 0±0 | 0±0 | 0.17±0.12 | 0.15±0.06 | 0.11±0.11 | 0.3±0.2 | 0±0 | 0.15±0.15 |
|  | P301S female |  | 0.03±0.03 | 0.03±0.03 | 0.03±0.03 | 0.09±0.06 | 0.15±0.07 | 0.15±0.12 | 0.45±0.31 | 0.2±0.15 | 0.23±0.16 |
|  |  |  |  |  |  |  |  |  |  |  |  |
| Gait score | WT male |  | 0.03±0.03 | 0.57±0.19 | 0.03±0.03 | 0±0 | 0.17±0.07 | 1±0.2 | 1±0.27 | 0.35±0.12 | 0.63±0.1 |
|  | P301S male |  | 0.24±0.1* | 1±0.2 | 0.43±0.16 | 0.57±0.2* | 0.52±0.14 | 1.24±0.37 | 1.57±0.34* | 1.83±0.4**** | 1.87±0.48*** |
|  | WT female |  | 0.06±0.04 | 0.56±0.1 | 0.07±0.07 | 0.33±0.14 | 0.37±0.13 | 0.74±0.16 | 0.81±0.18 | 0.39±0.14 | 0.41±0.16 |
|  | P301S female |  | 0.18±0.12 | 0.42±0.17 | 0±0 | 0.36±0.14 | 0.3±0.1 | 0.45±0.14 | 0.88±0.28 | 0.48±0.13 | 0.37±0.12 |
|  |  |  |  |  |  |  |  |  |  |  |  |
| Kyphosis score | **WT male** |  | **0±0** | **0.33±0.12** | **0.57±0.16** | **0.1±0.07** | **0.37±0.12** | **0.9±0.11** | **1.2±0.16** | **0.72±0.13** | **0.93±0.19** |
|  | **P301S male** |  | **0.06±0.06** | **0.96±0.15**** | **1±0.18** | **0.71±0.21**** | **1.24±0.2**** | **1.52±0.2*** | **2.08±0.2**** | **2.33±0.26****** | **2.53±0.2***** |
|  | WT female |  | 0.06±0.06 | 0.56±0.15 | 0.41±0.14 | 0.28±0.15 | 0.44±0.14 | 0.81±0.26 | 1.11±0.19 | 0.5±0.14 | 0.56±0.27 |
|  | P301S female |  | 0±0 | 0.64±0.13 | 0.61±0.15 | 1.23±0.23** | 0.8±0.13 | 1.09±0.17 | 1.36±0.2 | 0.75±0.13 | 1.5±0.21* |
|  |  |  |  |  |  |  |  |  |  |  |  |
| Nesting score(16h) | WT male | 3.3±0.21 |  |  | 2.83±0.21 |  |  | 2.96±0.21 |  |  | 2.93±0.19 |
|  | P301S male | 3.33±0.24 |  |  | 2.9±0.24 |  |  | 2.62±0.18 |  |  | 1.2±0.2**** |
|  | WT female | 3.33±0.29 |  |  | 2.74±0.21 |  |  | 3.41±0.22 |  |  | 2.56±0.18 |
|  | P301S female | 3.64±0.15 |  |  | 2.97±0.22 |  |  | 2.77±0.25 |  |  | 2.5±0.16 |
|  |  |  |  |  |  |  |  |  |  |  |  |
| Nesting score(24h) | **WT male** | **2.75±0.2** |  |  | **2.87±0.12** |  |  | **3.06±0.23** |  |  | **2.73±0.15** |
|  | **P301S male** | **3.28±0.15** |  |  | **2.86±0.2** |  |  | **2.07±0.17**** |  |  | **1±0****** |
|  | WT female | 3.17±0.24 |  |  | 2.52±0.2 |  |  | 2.83±0.22 |  |  | 2.67±0.25 |
|  | P301S female | 3.14±0.24 |  |  | 2.88±0.21 |  |  | 3.15±0.18 |  |  | 2.47±0.24 |

Statistical analysis between same gender group mice in same time point. Data are presented P301S mean ± S.E.M. statistical significance was calculated by ANOVA. *p<0.05, **p<0.01, ***p<0.001, ****p<0.0001.WT, wild type; P301S, P301S mouse. --The hiatus of 7-month-old grip force test data was because of mechanical defects.

Table S3. Behavioral tests for male and female P301S Tg mice and sex-matched WT littermates.
