# Supplementary material for: The behavioural and neuropathologic sexual dimorphism and absence of MIP-3α in tau P301S mouse model of Alzheimer’s disease
Source: J Neuroinflammation. 2020 Feb 24;17:72. doi: 10.1186/s12974-020-01749-w (PMC7041244; doi:10.1186/s12974-020-01749-w)
Supplement: Supplementary file 2 — Additional file 2:Table S1. The mount of living mice and the events in each time point. Table S2. The data of P301S mice and WT mice in behavioral tests. Table S3. Behavioral tests for male and female P301S Tg mice and sex-matched WT littermates. Table S4. The latency and number of target platform crossings of P301S mice and WT mice in the MWM test. Table S5. The latency and number of target platform crossings of male and female P301S Tg mice and sex-matched WT littermates in MWM test. [file 12974_2020_1749_MOESM2_ESM.zip › Additional file 2-Table S5.docx]

Table S5. The latency and number of target platform crossings of male and female P301S Tg mice and sex-matched WT littermates in MWM test.

| Group | | Latency during training test | | | | | Number of target platform crossings |
| --- | --- | --- | --- | --- | --- | --- | --- |
|  |  | Day 1 | Day 2 | Day 3 | Day 4 | Day 5 |  |
| 3-month-old | WT male | 41.88±3.67 | 38.04±3.61 | 34.3±3.68 | 23.81±2.8 | 21.34±2.9 | 1.2±0.29 |
|  | P301S male | 49.04±2.69 | 36.88±3.55 | 32.68±3.76 | 26.8±3.67 | 21.27±2.95 | 2±0.37 |
|  | WT female | 47.34±3.26 | 37.8±3.61 | 32.72±3.53 | 25.17±3.3 | 21.74±3.01 | 2.3±0.5 |
|  | P301S female | 47.73±3.26 | 34.41±3.35 | 27.31±3.43 | 18.95±2.8 | 18.77±2.87 | 1.5±0.31 |
|  |  |  |  |  |  |  |  |
| 6-month-old | WT male | 52.06±2.82 | 38.51±3.69 | 32.1±3.82 | 27.56±3.47 | 21.19±2.96 | 2.1±0.48 |
|  | P301S male | 54.18±2.32 | 30.5±3.73 | 24.07±3.41 | 20.35±3.03 | 26.1±3.25 | 2±0.39 |
|  | WT female | 48.78±3.43 | 39.49±3.83 | 26.01±3.79 | 22.56±3.52 | 21.18±3.17 | 2.78±0.62 |
|  | P301S female | 49.62±3.08 | 39.12±3.78 | 29.83±3.34 | 24.62±3.32 | 22.4±2.9 | 2.8±0.66 |
|  |  |  |  |  |  |  |  |
| 9-month-old | WT male | 45.28±3.75 | 28.76±3.7 | 26.32±3.44 | 26.47±3.46 | 22.62±2.98 | 1.75±0.59 |
|  | P301S male | 48.57±3.47 | 39.93±4.22 | 31.82±4.13 | 27.98±4.07 | 29.74±3.73 | 1.75±0.7 |
|  | WT female | 45.56±4.01 | 40.36±4.01 | 30.13±3.93 | 19.6±2.92 | 20.22±2.21 | 2.56±0.69 |
|  | P301S female | 50.91±3.05 | 38.69±3.96 | 31.66±3.84 | 24.12±3.43 | 18.42±2.54 | 3.56±1.13 |
|  |  |  |  |  |  |  |  |
| 12-month-old | WT male | 44.81±3.31 | 30.22±3.65 | 22.55±3.09 | 15.26±2.22 | 10.99±1.75 | 3.4±0.54 |
|  | P301S male | 40.04±5.93 | 32.81±6.4 | 23.32±5.99 | 21.9±4.3 | 14.65±3.67 | 3.4±1.4 |
|  | WT female | 42.27±3.76 | 28.85±3.61 | 22.38±2.83 | 14.8±1.84 | 13.32±2.56 | 4.63±0.6 |
|  | P301S female | 52.89±2.57* | 44.37±3.42** | 28.56±3.54 | 29.04±3.52**** | 24.72±3.25*** | 3.1±0.75 |

Data are presented as mean ± S.E.M. statistical significance was calculated by ANOVA. *p<0.05, **p<0.01, ***p<0.001, ****p<0.0001.WT, wild type mice; P301S, P301S Tg mice.
