# Supplementary material for: The behavioural and neuropathologic sexual dimorphism and absence of MIP-3α in tau P301S mouse model of Alzheimer’s disease
Source: J Neuroinflammation. 2020 Feb 24;17:72. doi: 10.1186/s12974-020-01749-w (PMC7041244; doi:10.1186/s12974-020-01749-w)
Supplement: Supplementary file 3 — Additional file 3. The original western blot figures of Fig. 4. [file 12974_2020_1749_MOESM3_ESM.pptx]

## Slide 1
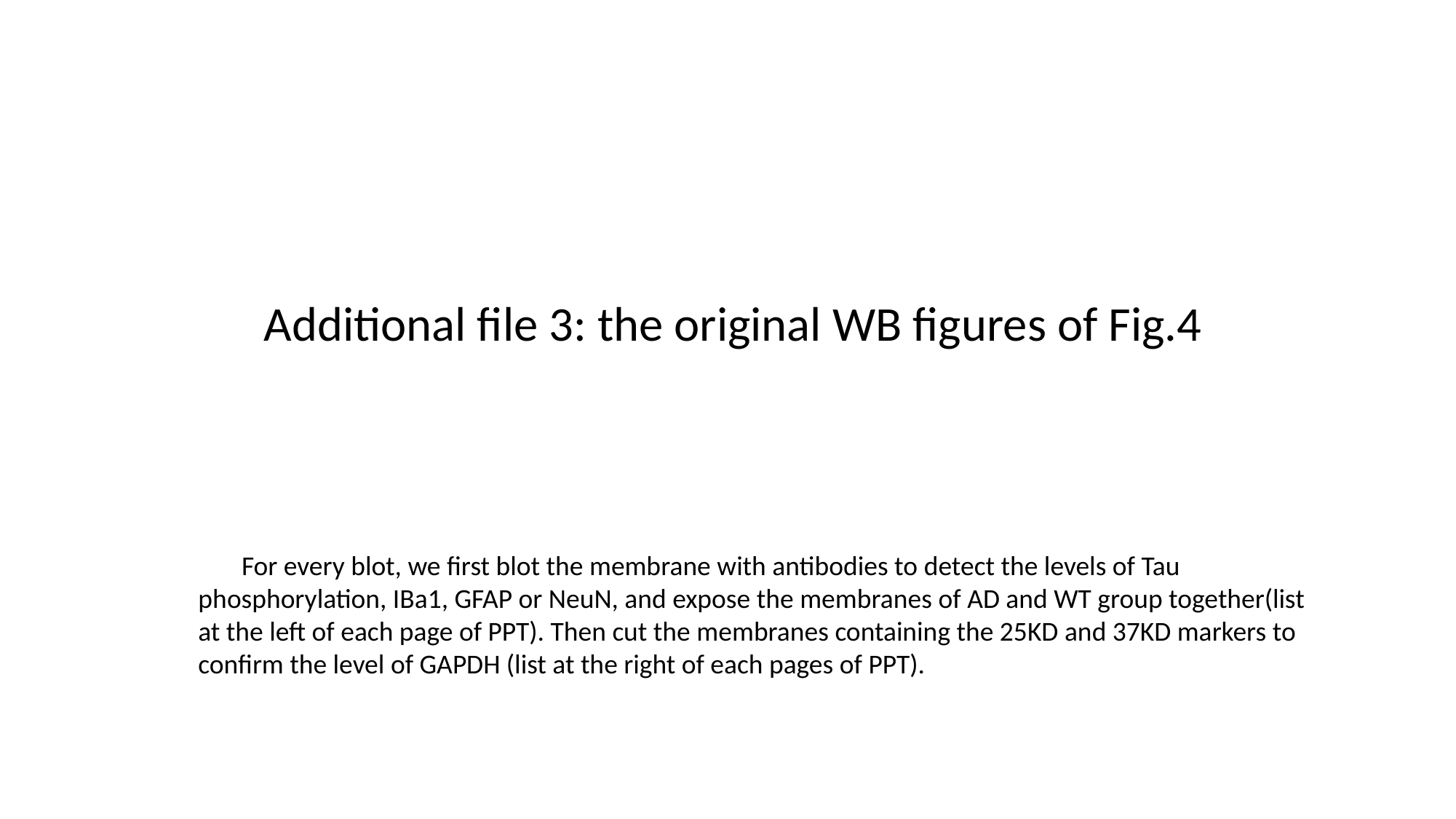

Additional file 3: the original WB figures of Fig.4
 For every blot, we first blot the membrane with antibodies to detect the levels of Tau phosphorylation, IBa1, GFAP or NeuN, and expose the membranes of AD and WT group together(list at the left of each page of PPT). Then cut the membranes containing the 25KD and 37KD markers to confirm the level of GAPDH (list at the right of each pages of PPT).

## Slide 2
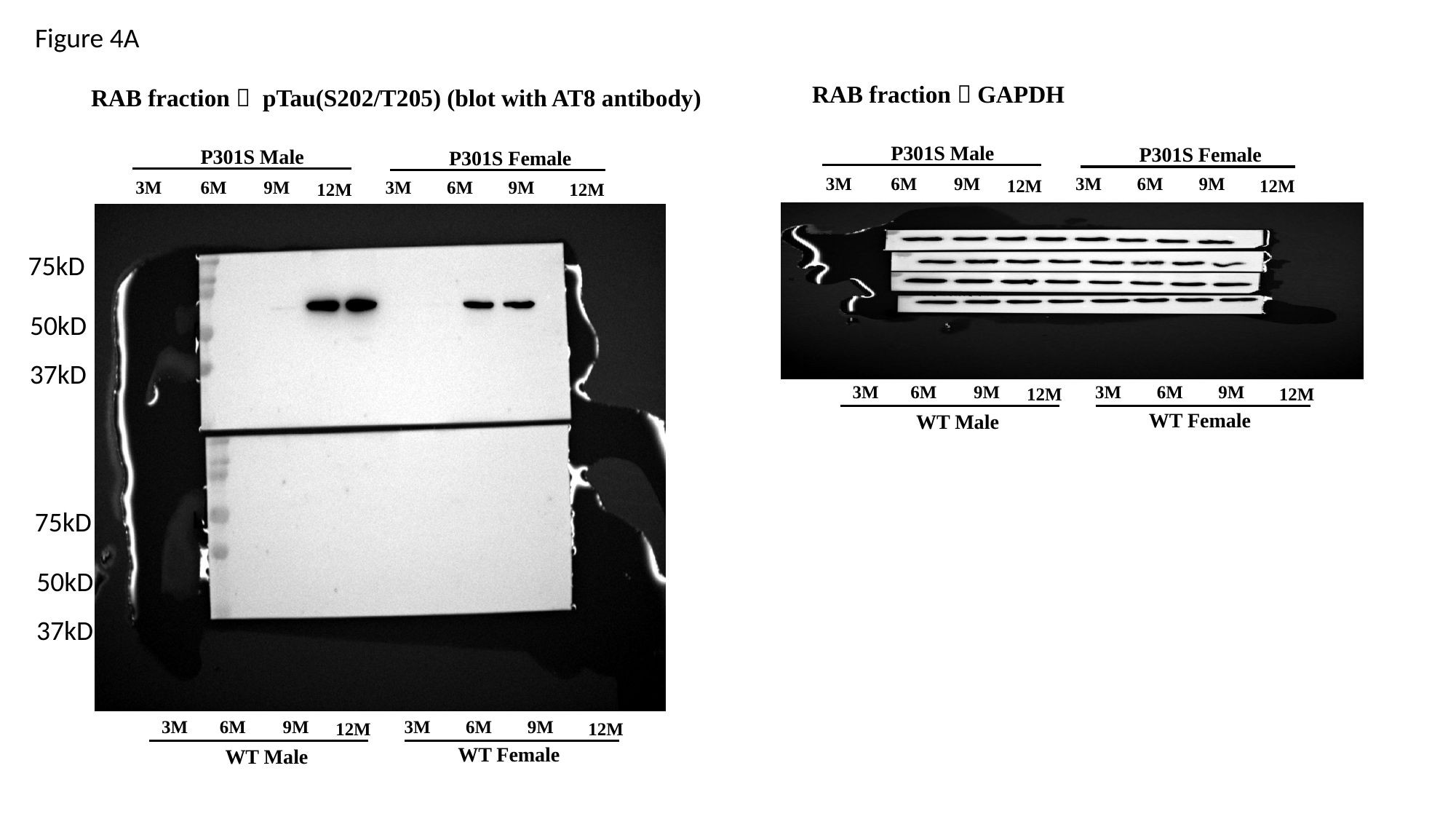

Figure 4A
RAB fraction：GAPDH
RAB fraction： pTau(S202/T205) (blot with AT8 antibody)
P301S Male
P301S Female
3M
6M
9M
12M
3M
6M
9M
12M
3M
6M
9M
12M
3M
6M
9M
12M
WT Female
WT Male
P301S Male
P301S Female
3M
6M
9M
12M
3M
6M
9M
12M
75kD
50kD
37kD
75kD
50kD
37kD
3M
6M
9M
12M
3M
6M
9M
12M
WT Female
WT Male

## Slide 3
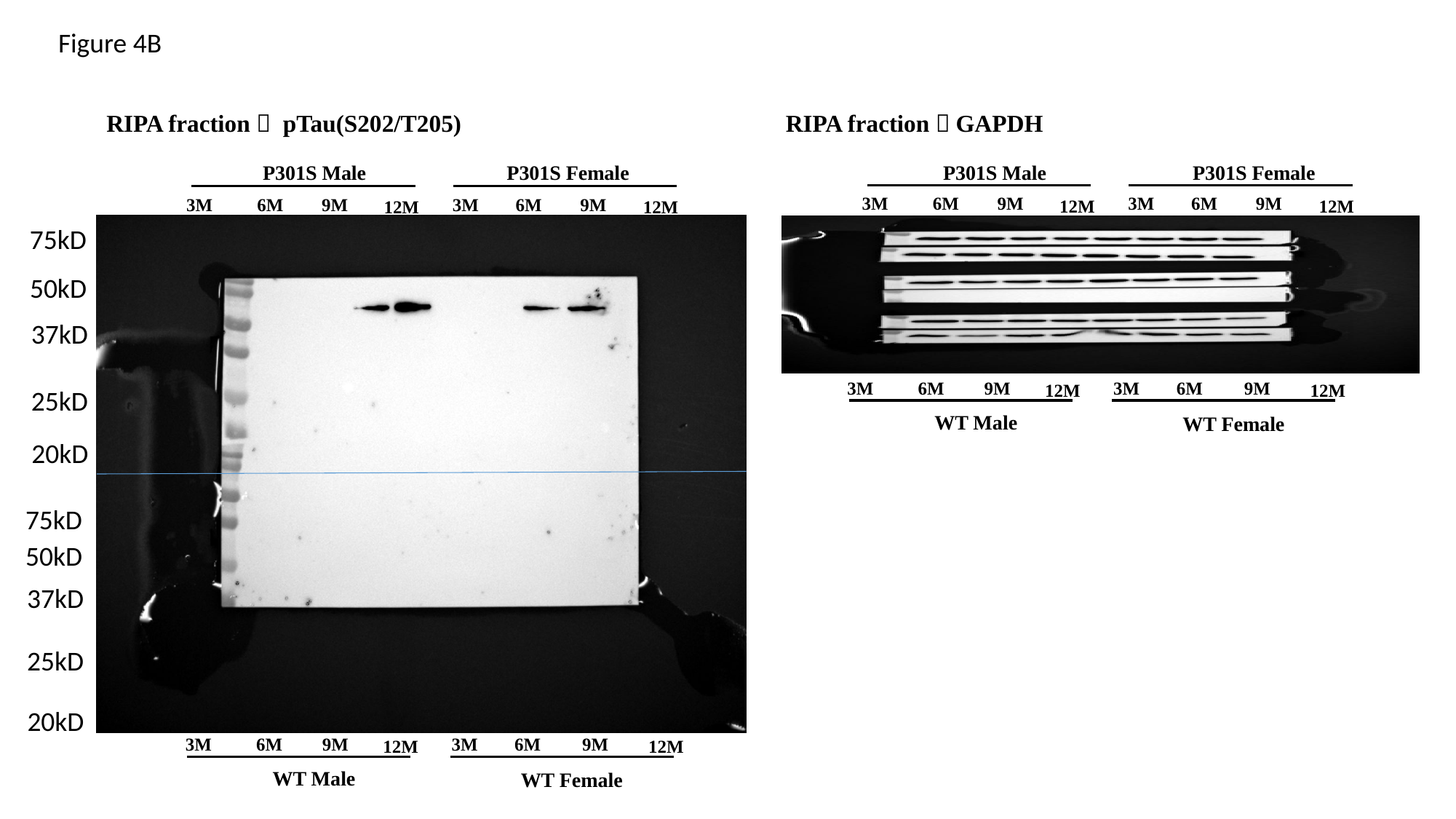

Figure 4B
RIPA fraction： pTau(S202/T205)
RIPA fraction：GAPDH
P301S Male
P301S Female
3M
6M
9M
12M
3M
6M
9M
12M
75kD
50kD
37kD
25kD
20kD
75kD
50kD
37kD
25kD
20kD
3M
6M
9M
12M
3M
6M
9M
12M
WT Male
WT Female
P301S Male
P301S Female
3M
6M
9M
12M
3M
6M
9M
12M
3M
6M
9M
12M
3M
6M
9M
12M
WT Male
WT Female

## Slide 4
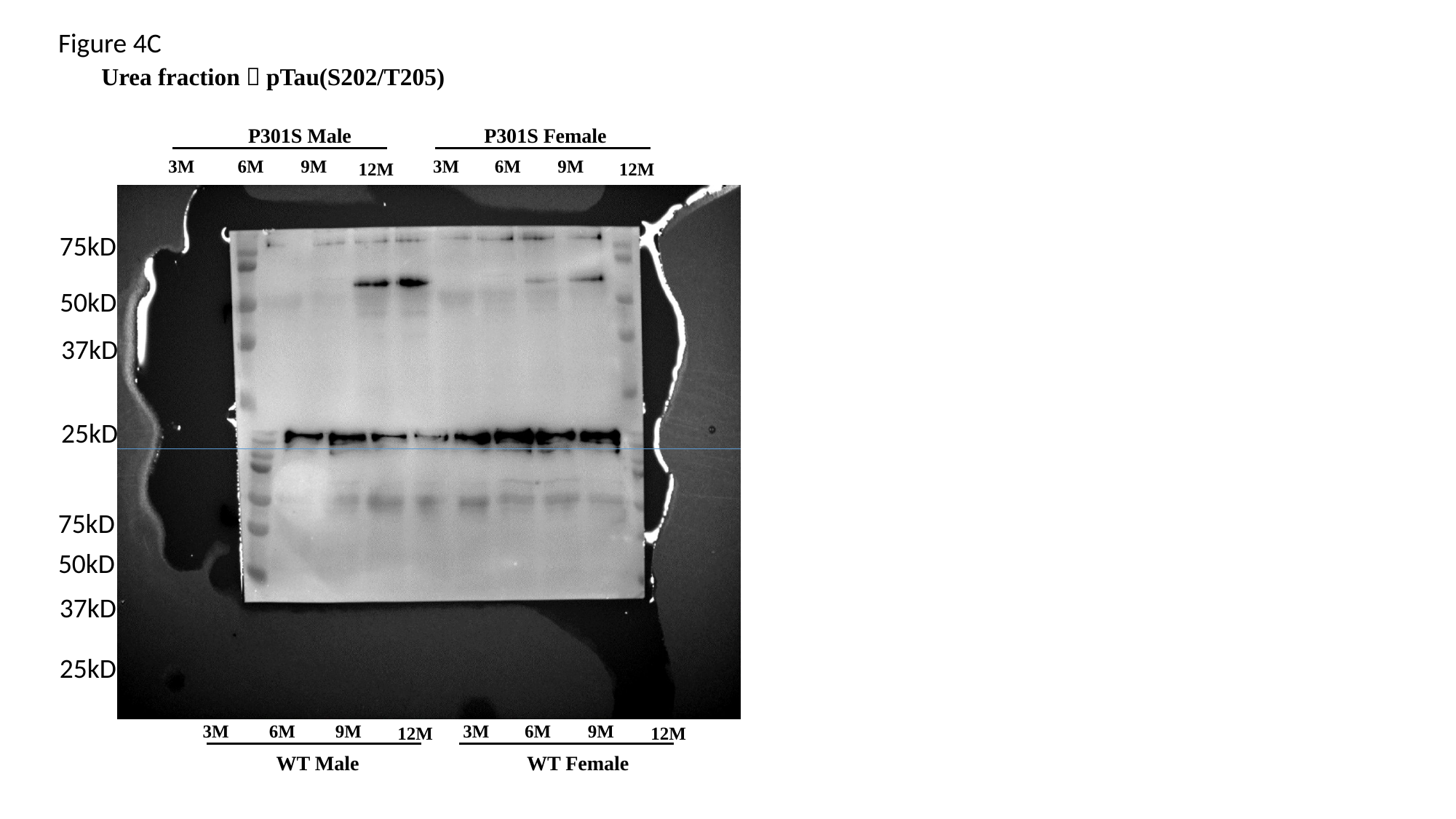

Figure 4C
Urea fraction：pTau(S202/T205)
P301S Male
P301S Female
3M
6M
9M
12M
3M
6M
9M
12M
75kD
50kD
37kD
25kD
75kD
50kD
37kD
25kD
3M
6M
9M
12M
3M
6M
9M
12M
WT Male
WT Female

## Slide 5
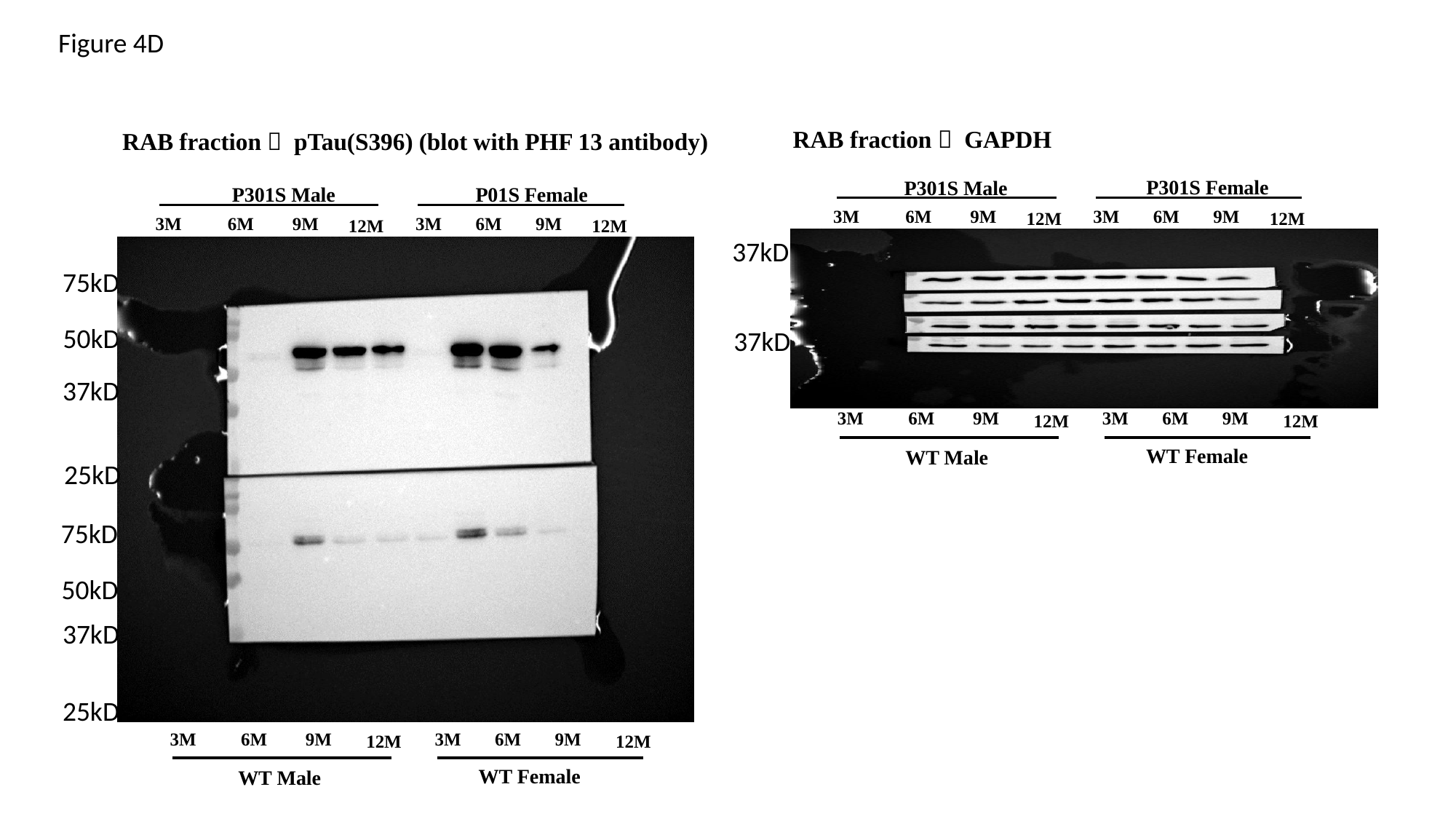

Figure 4D
RAB fraction： GAPDH
RAB fraction： pTau(S396) (blot with PHF 13 antibody)
P301S Female
P301S Male
3M
6M
9M
12M
3M
6M
9M
12M
37kD
37kD
3M
6M
9M
12M
3M
6M
9M
12M
WT Female
WT Male
P01S Female
P301S Male
3M
6M
9M
12M
3M
6M
9M
12M
75kD
50kD
37kD
25kD
75kD
50kD
37kD
25kD
3M
6M
9M
12M
3M
6M
9M
12M
WT Female
WT Male

## Slide 6
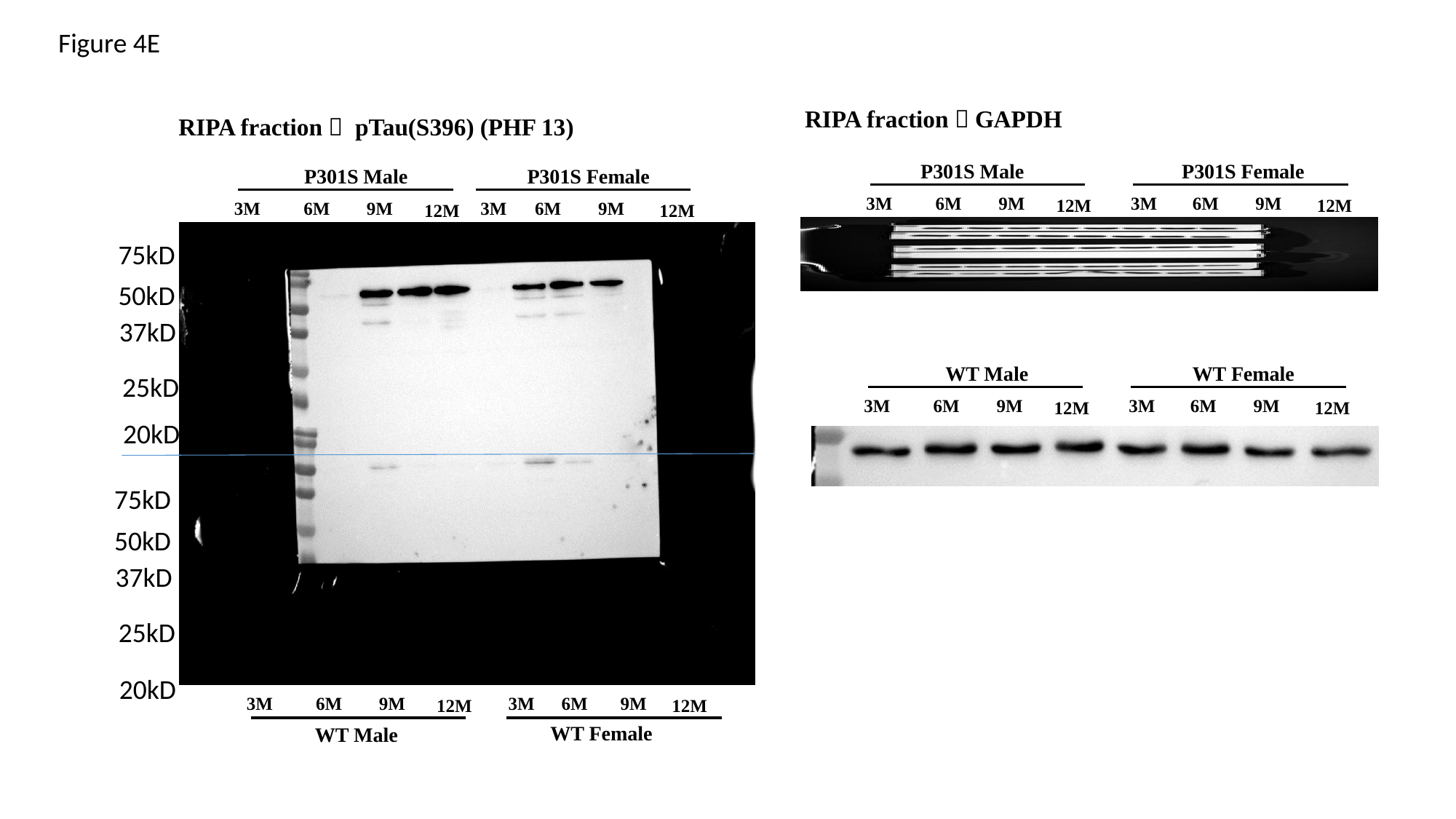

Figure 4E
RIPA fraction：GAPDH
RIPA fraction： pTau(S396) (PHF 13)
P301S Male
P301S Female
3M
6M
9M
12M
3M
6M
9M
12M
P301S Male
P301S Female
3M
6M
9M
12M
3M
6M
9M
12M
75kD
50kD
37kD
25kD
20kD
75kD
50kD
37kD
25kD
20kD
3M
6M
9M
12M
3M
6M
9M
12M
WT Female
WT Male
WT Male
WT Female
3M
6M
9M
12M
3M
6M
9M
12M

## Slide 7
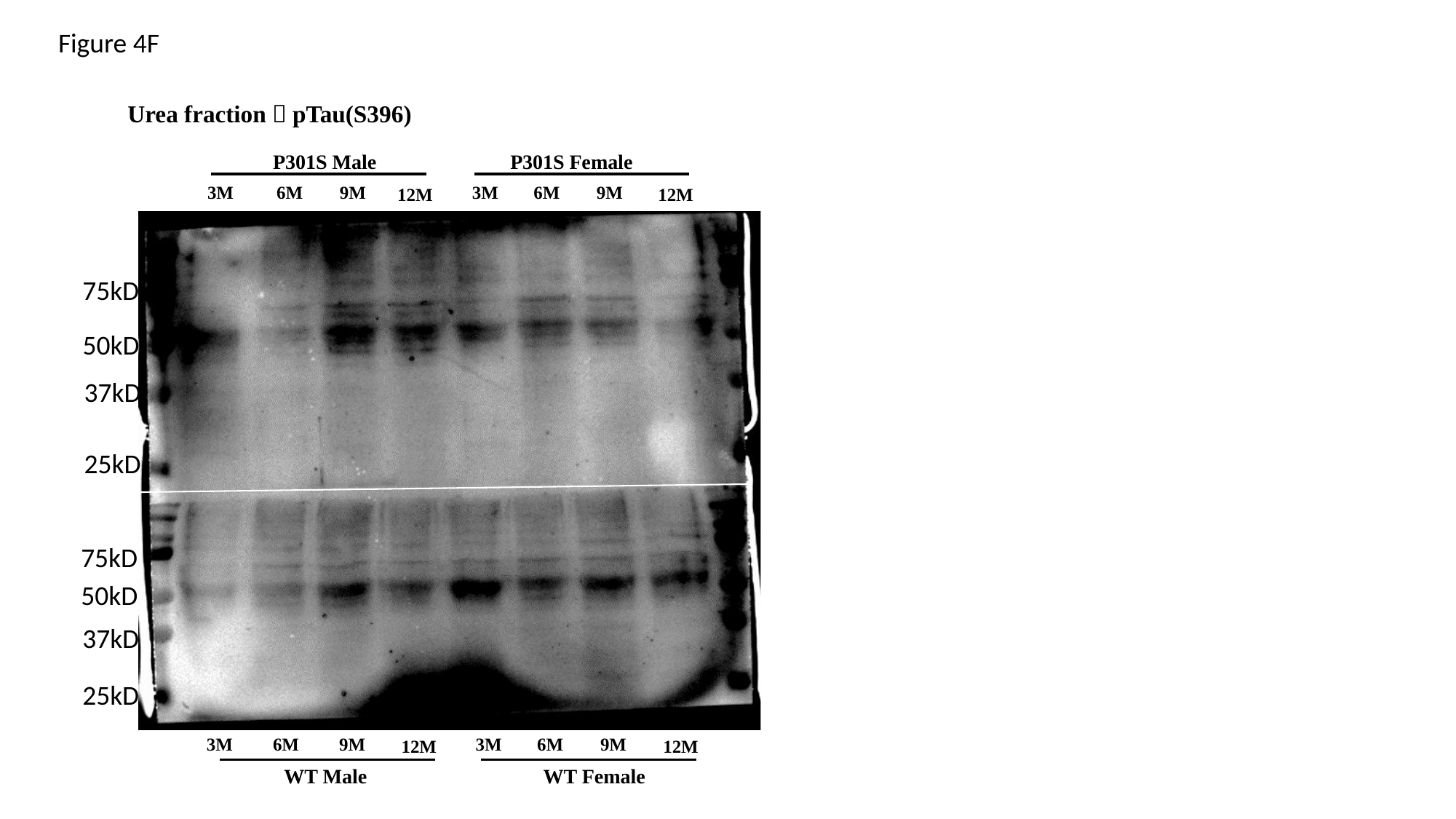

Figure 4F
Urea fraction：pTau(S396)
P301S Male
P301S Female
3M
6M
9M
12M
3M
6M
9M
12M
75kD
50kD
37kD
25kD
75kD
50kD
37kD
25kD
3M
6M
9M
12M
3M
6M
9M
12M
WT Male
WT Female

## Slide 8
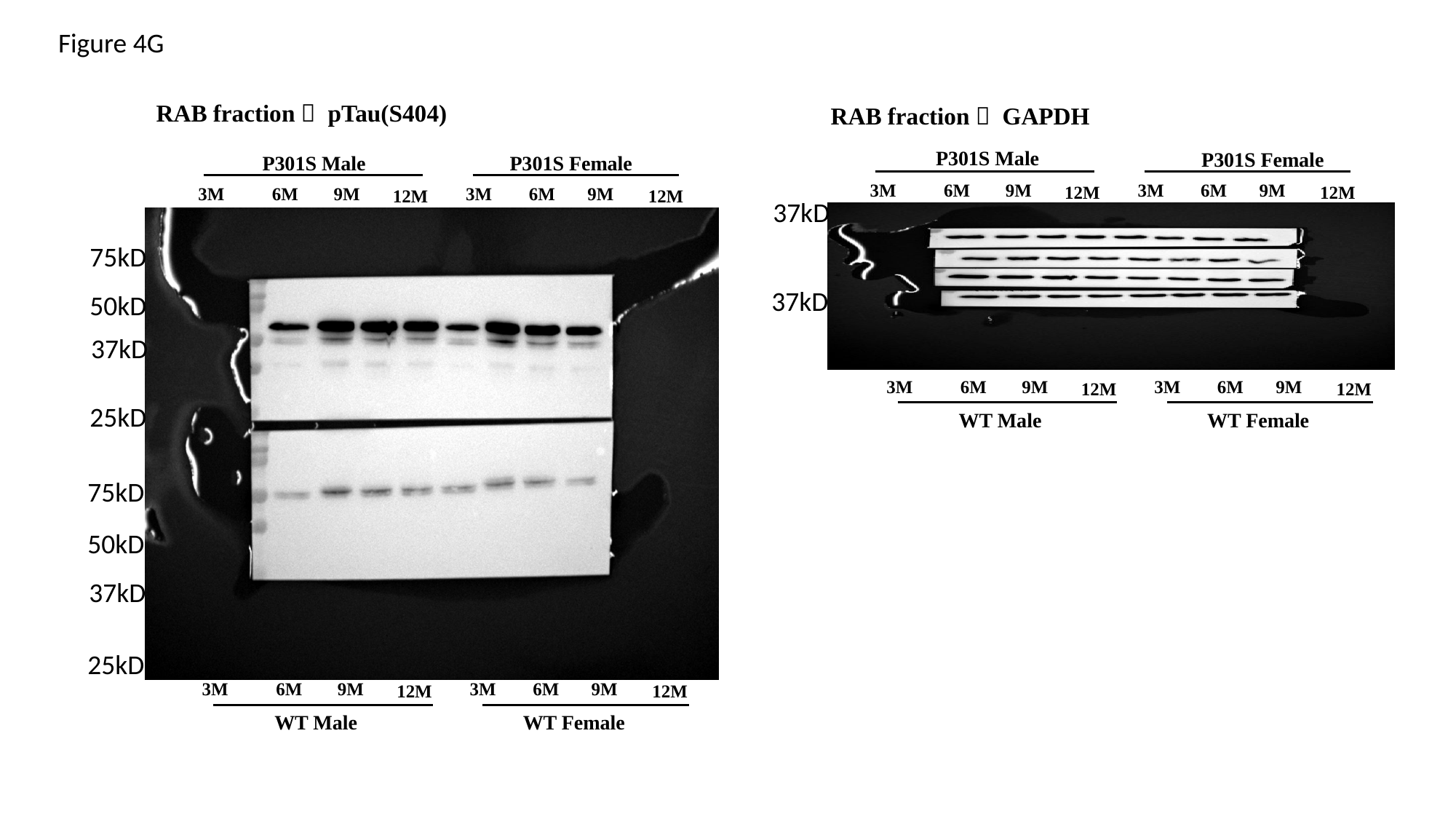

Figure 4G
RAB fraction： pTau(S404)
RAB fraction： GAPDH
P301S Male
P301S Female
3M
6M
9M
12M
3M
6M
9M
12M
37kD
37kD
3M
6M
9M
12M
3M
6M
9M
12M
WT Male
WT Female
P301S Male
P301S Female
3M
6M
9M
12M
3M
6M
9M
12M
75kD
50kD
37kD
25kD
75kD
50kD
37kD
25kD
3M
6M
9M
12M
3M
6M
9M
12M
WT Male
WT Female

## Slide 9
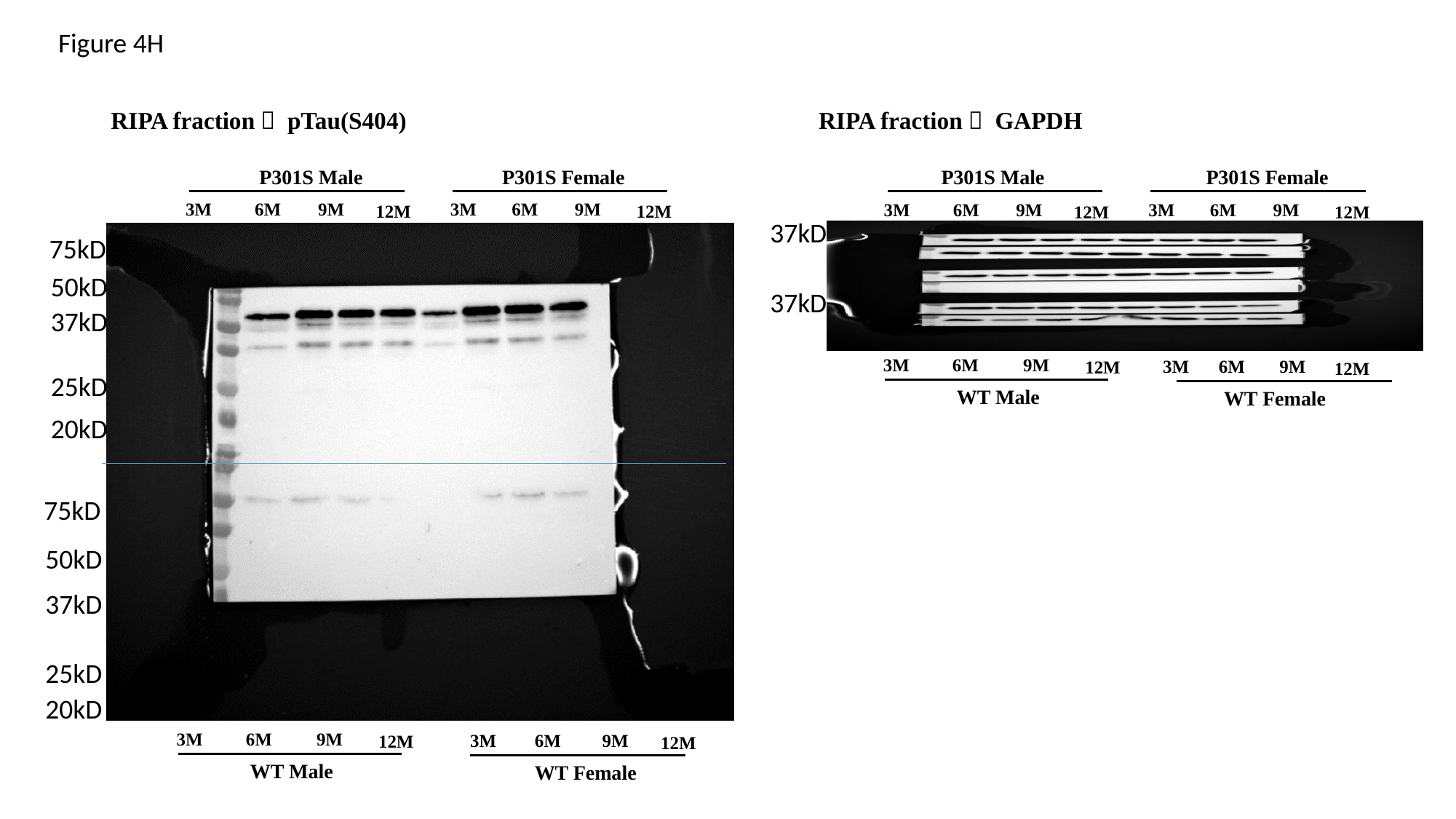

Figure 4H
RIPA fraction： pTau(S404)
RIPA fraction： GAPDH
P301S Male
P301S Female
3M
6M
9M
12M
3M
6M
9M
12M
75kD
50kD
37kD
25kD
20kD
75kD
50kD
37kD
25kD
20kD
3M
6M
9M
12M
3M
6M
9M
12M
WT Male
WT Female
P301S Male
P301S Female
3M
6M
9M
12M
3M
6M
9M
12M
37kD
37kD
3M
6M
9M
12M
3M
6M
9M
12M
WT Male
WT Female

## Slide 10
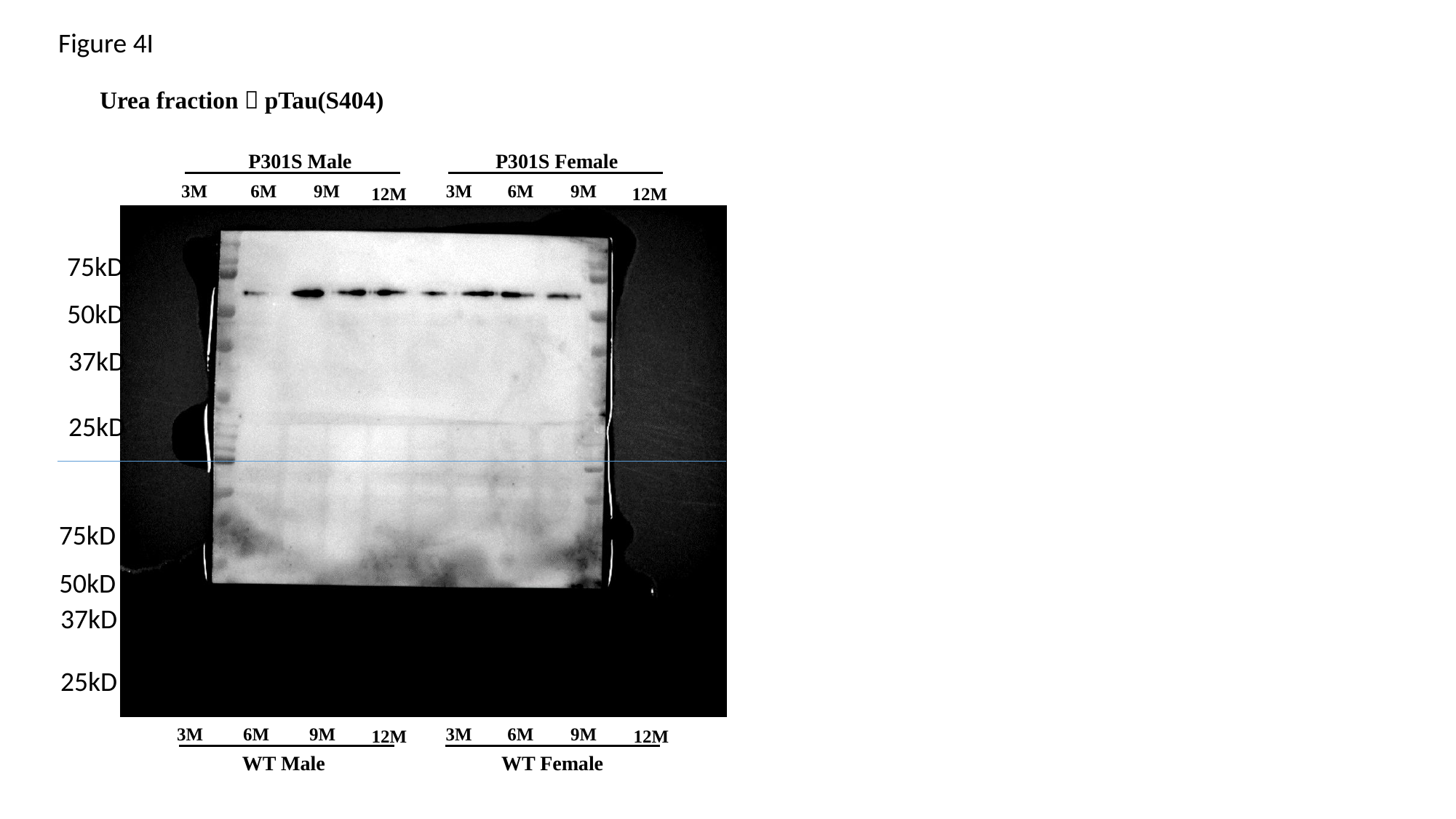

Figure 4I
Urea fraction：pTau(S404)
P301S Male
P301S Female
3M
6M
9M
12M
3M
6M
9M
12M
75kD
50kD
37kD
25kD
75kD
50kD
37kD
25kD
3M
6M
9M
12M
3M
6M
9M
12M
WT Male
WT Female

## Slide 11
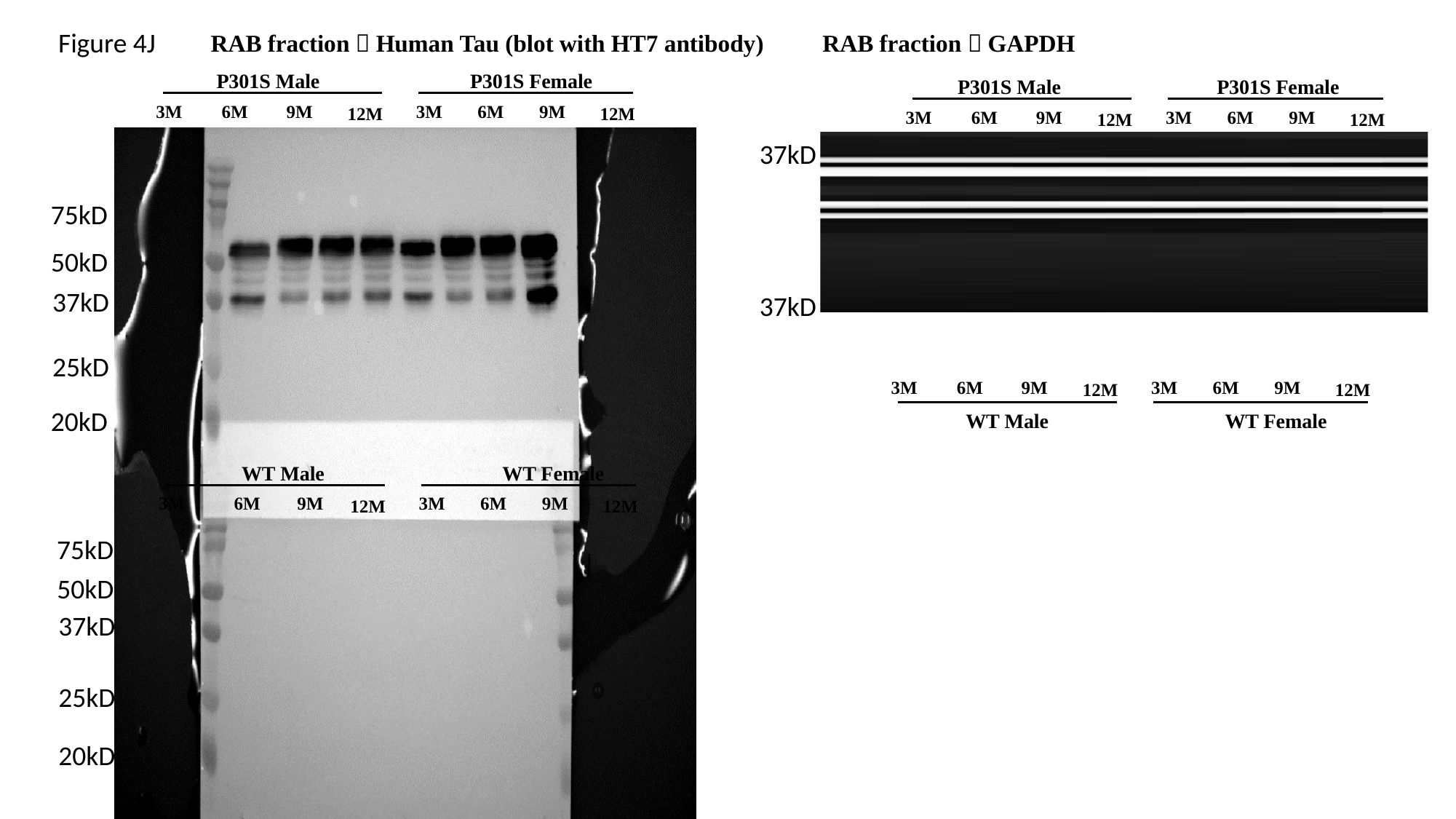

Figure 4J
RAB fraction：Human Tau (blot with HT7 antibody)
RAB fraction：GAPDH
P301S Male
P301S Female
3M
6M
9M
12M
3M
6M
9M
12M
75kD
50kD
37kD
25kD
20kD
WT Male
WT Female
3M
6M
9M
12M
3M
6M
9M
12M
75kD
50kD
37kD
25kD
20kD
P301S Male
P301S Female
3M
6M
9M
12M
3M
6M
9M
12M
37kD
37kD
3M
6M
9M
12M
3M
6M
9M
12M
WT Male
WT Female

## Slide 12
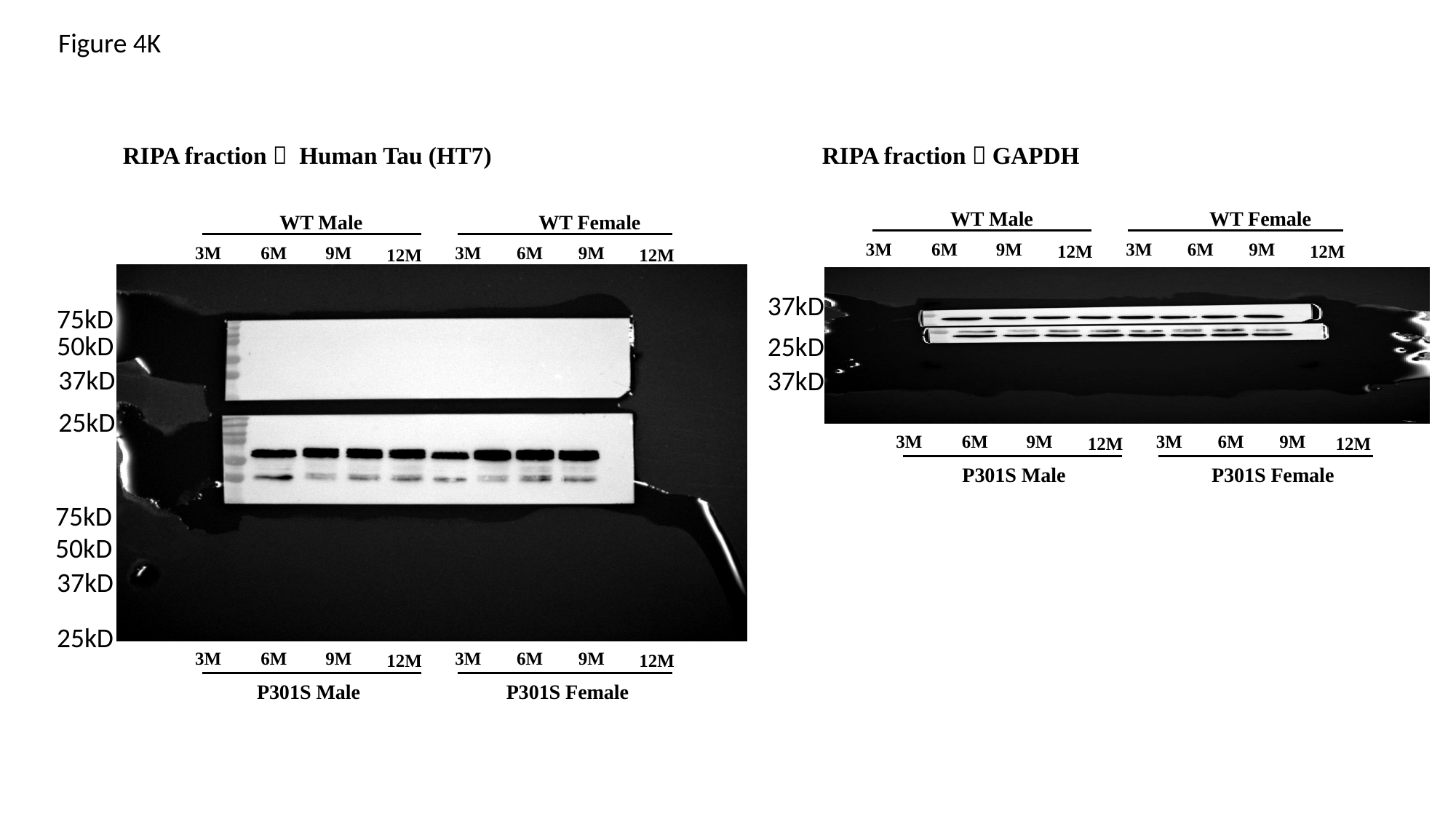

Figure 4K
RIPA fraction： Human Tau (HT7)
RIPA fraction：GAPDH
WT Male
WT Female
3M
6M
9M
12M
3M
6M
9M
12M
37kD
25kD
37kD
3M
6M
9M
12M
3M
6M
9M
12M
P301S Male
P301S Female
WT Male
WT Female
3M
6M
9M
12M
3M
6M
9M
12M
75kD
50kD
37kD
25kD
75kD
50kD
37kD
25kD
3M
6M
9M
12M
3M
6M
9M
12M
P301S Male
P301S Female

## Slide 13
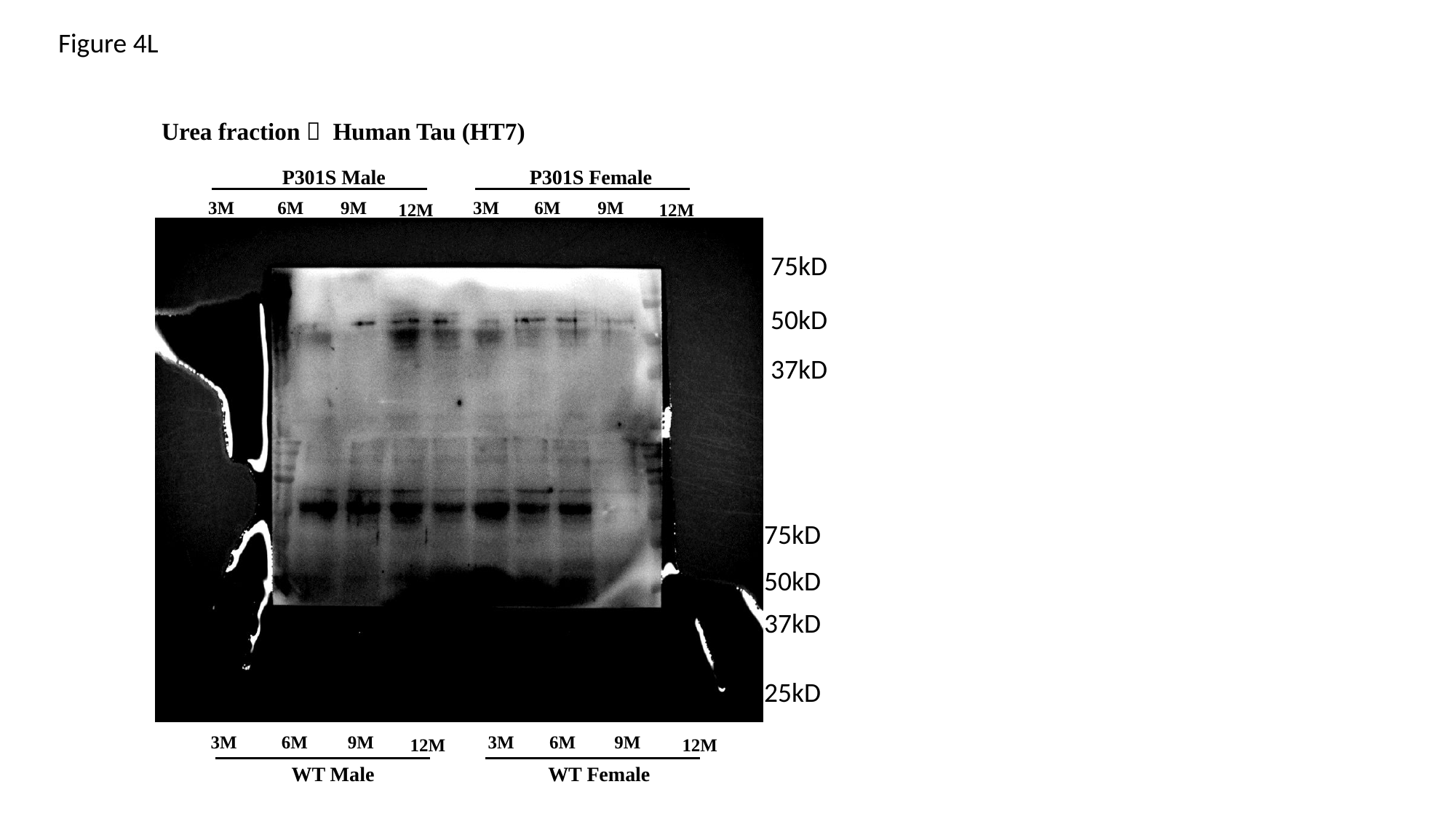

Figure 4L
Urea fraction： Human Tau (HT7)
P301S Male
P301S Female
3M
6M
9M
12M
3M
6M
9M
12M
75kD
50kD
37kD
75kD
50kD
37kD
25kD
3M
6M
9M
12M
3M
6M
9M
12M
WT Male
WT Female

## Slide 14
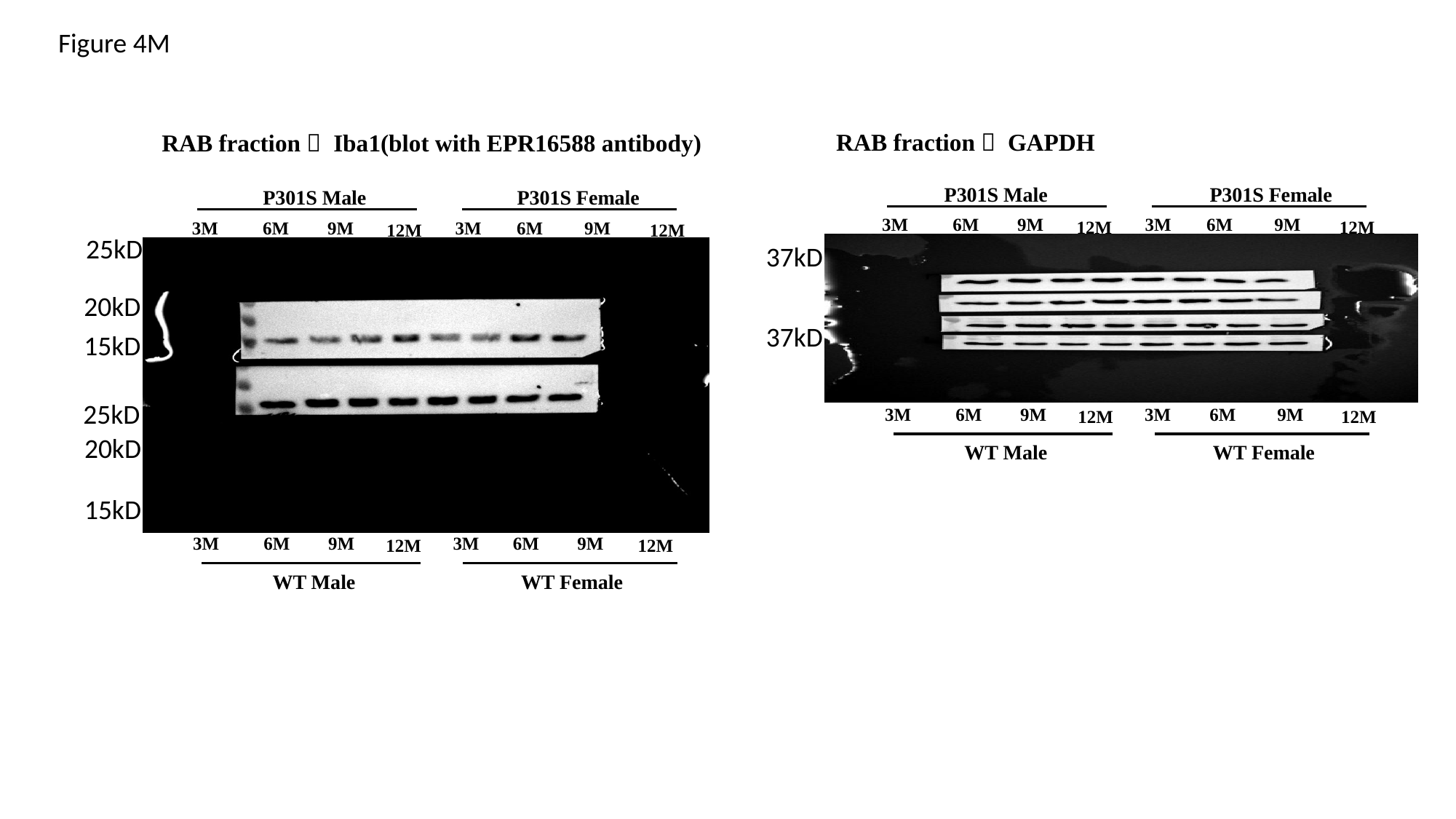

Figure 4M
RAB fraction： GAPDH
RAB fraction： Iba1(blot with EPR16588 antibody)
P301S Male
P301S Female
3M
6M
9M
12M
3M
6M
9M
12M
37kD
37kD
3M
6M
9M
12M
3M
6M
9M
12M
WT Male
WT Female
P301S Male
P301S Female
3M
6M
9M
12M
3M
6M
9M
12M
25kD
20kD
15kD
25kD
20kD
15kD
3M
6M
9M
12M
3M
6M
9M
12M
WT Male
WT Female

## Slide 15
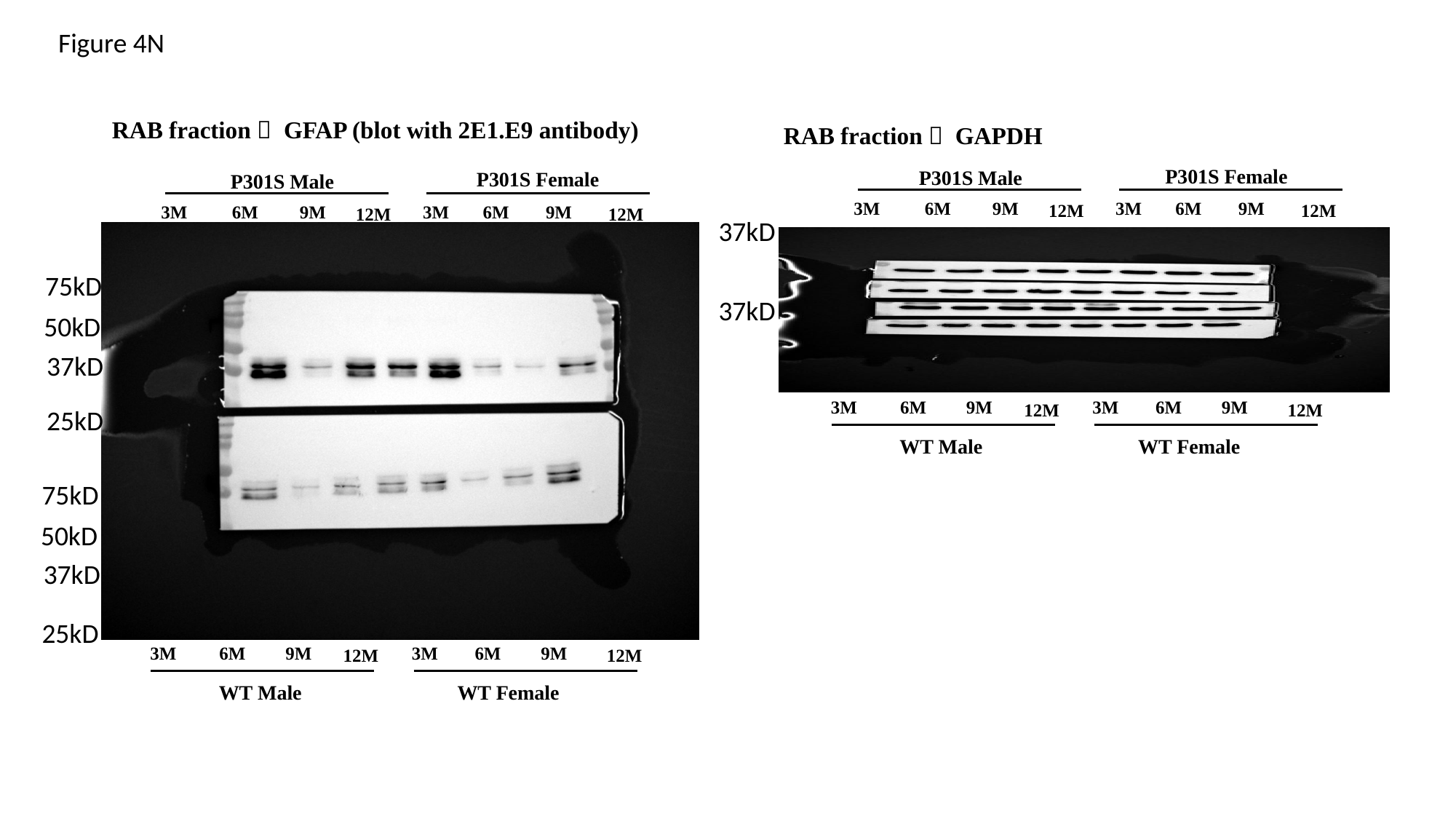

Figure 4N
RAB fraction： GFAP (blot with 2E1.E9 antibody)
RAB fraction： GAPDH
P301S Female
P301S Male
3M
6M
9M
12M
3M
6M
9M
12M
37kD
37kD
3M
6M
9M
12M
3M
6M
9M
12M
WT Male
WT Female
P301S Female
P301S Male
3M
6M
9M
12M
3M
6M
9M
12M
75kD
50kD
37kD
25kD
75kD
50kD
37kD
25kD
3M
6M
9M
12M
3M
6M
9M
12M
WT Male
WT Female

## Slide 16
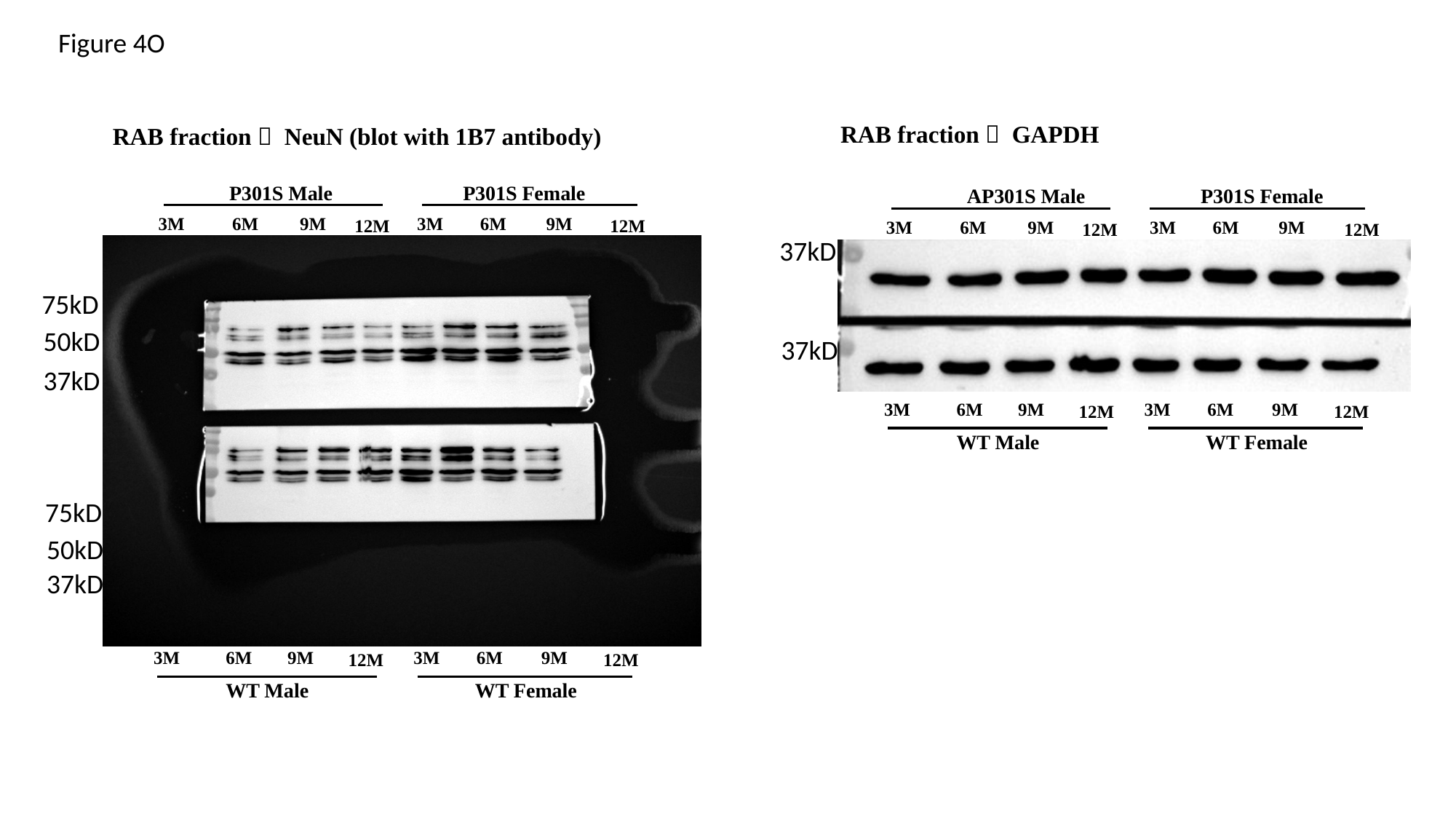

Figure 4O
RAB fraction： GAPDH
RAB fraction： NeuN (blot with 1B7 antibody)
P301S Male
P301S Female
3M
6M
9M
12M
3M
6M
9M
12M
75kD
50kD
37kD
75kD
50kD
37kD
3M
6M
9M
12M
3M
6M
9M
12M
WT Male
WT Female
AP301S Male
P301S Female
3M
6M
9M
12M
3M
6M
9M
12M
37kD
37kD
3M
6M
9M
12M
3M
6M
9M
12M
WT Male
WT Female
